# The effect of USM-IAM-based counselling vs standard counselling on insulin adherence, FBS and HbA1c among patients with uncontrolled type 2 diabetes mellitus (T2DM): a randomised controlled trial

**DOI:** 10.1186/s12902-024-01577-6

**Published:** 2024-07-18

**Authors:** Aida Maziha Zainudin, Aida Hanum Ghulam Rasool, Najib Majdi Yaacob, Rosediani Muhamad, Wan Mohd Izani Wan Mohamed

**Affiliations:** 1 Department of Pharmacology, School of Medical Sciences, Universiti Sains Malaysia, 6150 Kota Bharu, Kelantan Malaysia; 2 Hospital Universiti Sains Malaysia, Kota Bharu, Kelantan Malaysia; 3 Biostatistics and Research Methodology Unit, School of Medical Sciences, Universiti Sains Malaysia, 16150 Kota Bharu, Kelantan Malaysia; 4 Department of Family Medicine, School of Medical Sciences, Universiti Sains Malaysia, 16150 Kota Bharu, Kelantan Malaysia; 5 Department of Internal Medicine, School of Medical Sciences, Universiti Sains Malaysia, 16150 Kota Bharu, Kelantan Malaysia

**Keywords:** Insulin adherence, Insulin adherence module, Diabetes education, Type 2 diabetes mellitus, Diabetes, Universiti Sains Malaysia-insulin adherence module, USM-IAM

## Abstract

**Background:**

Many patients with T2DM on insulin are not optimally controlled despite receiving standard diabetes education counselling. Poor insulin adherence may be a contributing factor. We developed and evaluated a new module [Universiti Sains Malaysia-Insulin Adherence Module (USM-IAM)] on insulin-treated patients with poorly controlled diabetes.

**Methods:**

Eligibility criteria are those diagnosed with T2DM, aged between 18 and 65 years, with HbA1c between 8 and 15% and on insulin therapy for 1 year. Patients were randomly allocated to receive either the USM-IAM-based counselling or the standard counselling (SC) at baseline and the second visit. Patients were instructed to adjust insulin doses based on blood glucose levels. Outcomes were changes in adherence score, FBS and HbA1c levels from baseline to 3 months and baseline to sixth month.

**Results:**

Ninety patients were randomised to each group. The baseline sociodemographic and clinical characteristics were homogenous among groups. Ninety patients were analysed for each group. Adherence score changes between baseline to 3 months were − 8.30 (− 11.47, − 5.14) in USM-IAM-based counselling group (USM-IAM) and − 7.64 (− 10.89, − 4.40) in standard counselling group (SCG), between baseline to sixth month were − 10.21 (− 13.40, − 7.03) in USM-IAM and − 10.79 (− 14.64, − 6.97) in SCG. FBS changes between baseline to 3 months were 1.374 (0.25, 2.50) in USM-IAM and 0.438 (− 0.66, 1.54) in SCG, and between baseline to sixth month were 1.713 (0.473, 2.95) in USM-IAM and 0.998 (− 0.02, 2.01) in SCG. HbA1c changes between baseline to 3 months were 1.374 (0.25, 2.50) in USM-IAM and 0.547 (0.12, 0.98) in SCG, and between baseline to sixth month were 1.03 (0.65, 1.41) in USM-IAM and 0.617 (0.20, 1.03) in SCG. Between-subjects effects for all outcomes were not statistically significant.

**Conclusion:**

Both groups had significant improvements in adherence score and HbA1c with time, with higher improvement in patients receiving the USM-IAM. FBS reductions were significant in the intervention group but not in the control group.

**Trial registration:**

This study protocol is registered with Clicaltrials.gov with ID NCT05125185 dated 17th November 2021.

**Supplementary Information:**

The online version contains supplementary material available at 10.1186/s12902-024-01577-6.

## Background

Type 2 diabetes mellitus (T2DM) is the most common form of diabetes among adults. In 2021, approximately 537 million people globally were reported to have diabetes, with projections estimating an increase to 783 million by 2045 [1]. The prevalence of T2DM among Malaysians has surged over the past two decades, from 6.3% in 1986 to 8.3% in 1996 [2] to 13.4% in 2015 and further climbing to 18.3% in 2019 [3].

Patients diagnosed with diabetes mellitus are frequently prescribed oral glucose-lowering drugs (OGLDs) and/or insulin and/or other injectable agents. This regimen is combined with a healthy diet and increased physical activity to achieve glycaemic control. Both Malaysian and international guidelines recommend the combination of OGLDs with insulin/injectable agents when patients need more efficacious approaches to attain their glycaemic goals [4, 5].

Despite the development of injectables such as dulaglutide and semaglutide, which have proven efficacy in reducing HbA1c and cardiac events [6, 7], insulin continues to be the key treatment for many patients due to its availability and affordability. The majority of patients with diabetes mellitus will ultimately need insulin 8 to 10 years after the diagnosis of diabetes to maintain good glycaemic control [8, 9]. Most of those on single insulin injection will require intensification within 3 years of insulin initiation [10]. Despite the increase in the number of patients using insulin, the percentage of patients who achieve targeted HbA1c levels remains low. In Malaysia, 32.4% of patients achieved the HbA1c target of ≤6.5% [11].

A literature review revealed that adherence to insulin is generally poor among people with diabetes, and the adherence rates are lower than those for oral hypoglycaemic agents [12, 13]. Several factors contribute to nonadherence to insulin, including pain associated with injections [14], fear of hypoglycemia and weight gain [15], interference of injections with daily routines and embarrassment associated with administering insulin in public [16]. In Malaysia, the same factors were identified as barriers to insulin adherence [17], along with other issues such as myths and misconceptions toward insulin [18] and forgetfulness [13].

Reports have emphasised that poor diabetic control among patients treated with insulin primarily results from a lack of understanding of the disease, a lack of knowledge of glycaemic targets, and a lack of knowledge of diabetes self-care, particularly self-insulin dose adjustment [19]. Inadequate knowledge of diabetes leads to low adherence to self-care practices such as a diabetes diet, exercise and self-monitoring of blood glucose (SMBG) [20]. There is a growing body of evidence highlighting the importance of education in improving HbA1c [21–23] and increasing diabetes knowledge [24].

Based on local data, more than 90% of our patients are T2DM and most of these patients have poor diabetes control. Despite 82% of patients having been counselled at least once by diabetic educators, the mean HbA1c of patients in the treatment centre was 10% [25].

In response, we have introduced a new section on nonadherence to insulin therapy, which includes its definition, causes of nonadherence, and suggested solutions to problems with insulin injection in a newly developed module. The introduction of these contents to the USM-IAM may provide more insight to patients about insulin nonadherence, the causes and their solutions. This additional information may lead to better insulin adherence among patients. Thus, in this study we aimed to compare the effects of diabetes counselling: based on this newly developed module (USM-IAM) with that of standard counselling (SC) on insulin adherence, FBS and HbA1c among patients with uncontrolled T2DM in a 6 months’ duration study.

## Methods

### Study design and approval

The study was a single-centre, randomised, parallel, controlled trial with a 6-month follow-up period. It was performed at the Endocrine Clinic of Hospital Universiti Sains Malaysia, a tertiary facility hospital located on the east coast region of Malaysia. The study protocols have been performed in accordance with the Declaration of Helsinki and was approved by the Research Ethics Committee of Universiti Sains Malaysia, with identity number USM/JEPeM/20110605, and registered with ClinicalTrials.gov with ID NCT05125185 dated 17th November 2021. This study was reported in adherence to CONSORT guidelines [26].

Prior to the commencement of the study, the educators who would be involved in providing the counselling were identified. Two educators were allocated to each group. Standardisations for counselling were conducted, instructing educators who would administer standard counselling (SC) to use a specified flip chart prepared based on a manual [27], along with insulin pen models and handouts. In contrast, educators in USM-IAM were trained in counselling using the module.

### Sample size estimation & randomization

The minimum required sample size was calculated by comparing the means of the IAQDM score with a standard deviation of 4.3 [20], difference of 2.0, precision of 0.05, and alpha and power of 80%. The minimum sample was 148, exaggerated to 178 (20% dropout rate). A co-researcher generated the group allocation list through permuted block randomisation [2, 4, 6] [28] with equal allocation (1:1). A research assistant printed the group allocation list and concealed it in 180 envelopes. The researcher opened the envelopes chronologically on the patients’ recruitment day. Recruitment took place from August 2021 through July 2022.

Patients aged between 18 and 65 years, diagnosed with T2DM as defined by International Diabetes Federation (IDF), prescribed insulin for at least 1 year, and had an HbA1c level between 8 and 15 were included in the study. The HbA1c range was selected to focus on educating patients with poor glycaemic control. Patients were excluded if they did not understand the Malay language or were illiterate; had severe diabetes complications such as chronic kidney disease, heart failure, or severe proliferative diabetic retinopathy; experienced recurrent hypoglycemia or hypoglycemia unawareness; or were obese with a body mass index (BMI) ≥ 40 kg/m^2^. Eligible patients were invited to participate, and for those who agreed, additional information was provided before they were asked to sign the study’s consent form.

### Data collection

On the recruitment day, blood was drawn to confirm patient eligibility and establish baseline glycaemic indices. Once eligibility was verified, patients completed forms gathering information on sociodemographic and clinical characteristics. The participants self-reported their adherence to insulin therapy, SMBG, insulin dose, dietary adjustment and problems with insulin therapy using the Insulin Adherence Questionnaire for patients with Diabetes Mellitus (IAQDM) [25]. The Morisky Medication Adherence Scale-8 (MMAS-8) and the Malaysian Medication Adherence Scale (MALMAS) are the most widely used questionnaires for assessing medication adherence in Malaysia [29]. However, these questionnaires were not utilised in this study because they do not evaluate aspects of insulin adherence such as glucose monitoring, insulin dose, dietary adjustment, or problems with injections. Instead, we employed the IAQDM, which has been validated for assessing insulin adherence among the Malay population in our study centre [25]. Nasruddin *at. el.* utilised the questionnaire among 249 patients with T2DM treated with insulin from five health clinics in the Klang district, Selangor, Malaysia [30]. The researcher or her assistant addressed any clarification needed for the questions. Patients were subsequently randomly assigned to one of two groups, standard counselling (SC) or USM-IAM, based on the allocations in the envelopes.

Each counselling session was conducted individually by a diabetic educator. Educators in the SC group (SCG) educated patients using a flip chart and insulin pen models. The patients were informed about blood sugar targets, insulin injection techniques, insulin dose modification and how to recognise as well as manage hypoglycemia events. All patients were given SMBG diaries to record their blood sugar levels and any incidents of hypoglycemia. The participants were instructed and encouraged to intensify their insulin dosage weekly, and a handout containing instructions to optimise their insulin dosage based on their SMBG readings was given. The researcher’s and educator’s phone numbers were provided for any questions or concerns.

In the USM-IAM, educators provided instruction to participants using the USM-IAM module. The development and validation of the module, which integrates information obtained from focus group findings among patients, were performed. The content validity index (CVI) of the 20 items in the newly produced module was 0.92, and the face validity agreement rate ranged from 86 to 97%. The module was distributed as a 40-page, A5-sized booklet containing text, 44 pictures and 12 tables. The USM-IAM approach differed from the SC approach because it explained the relationship between insulin and diabetes, defined nonadherence to insulin, discussed causes of nonadherence, provided measures to overcome nonadherence and covered fasting safely with insulin therapy. Most of the population in this study fasted at least 1 month every year, with some of them fasting more, as encouraged by the religion. Educators guided participants through the module, and a summary of its content is provided in Table 1.

**Table 1:** Summary of USM-IAM module content

Topics	Learning Outcomes	Content
Diabetes and insulin	To give general information to diabetic patients about diabetes and its relationship with insulin	Diabetes definition Types of diabetes The relationship between diabetes and insulin Types of insulin Why do people with diabetes inject different insulin types? Insulin regimes
Nonadherence to insulin treatment and the consequences of nonadherence	Improve the knowledge and understanding of diabetic patients about nonadherence to insulin treatment and its outcome	Definition of nonadherence to insulin treatment The effect of nonadherence to insulin treatment
Causes of nonadherence to insulin treatment and suggested solutions.	Improve the knowledge and understanding of diabetic patients about the causes of nonadherence to insulin injections and how to overcome them.	Insulin side effects Problems with insulin injections Negative attitude towards insulin Expensive cost of monitoring Wrong perception of insulin Myths on insulin
Empowering diabetes self-care.	Improve knowledge and understanding of target sugar control, blood pressure, cholesterol, and ideal body weight. Improve the motivation of diabetic patients to check sugar levels and adjust insulin doses.	How to control diabetes How to do SMBG How to adjust insulin doses based on the sugar level
Fasting safely with insulin treatment	Improve the knowledge of diabetic patients about methods to fast safely despite injecting insulin	When should I check my sugar levels while fasting? How to modify the insulin dose while fasting? When should I break my fast?

Participants were encouraged to ask questions based on their problems and discuss practical solutions with the educator. Participants were given the module and could refer to it freely when needed, with the request not to share it with others until the study’s completion.

**Fig. 1:**
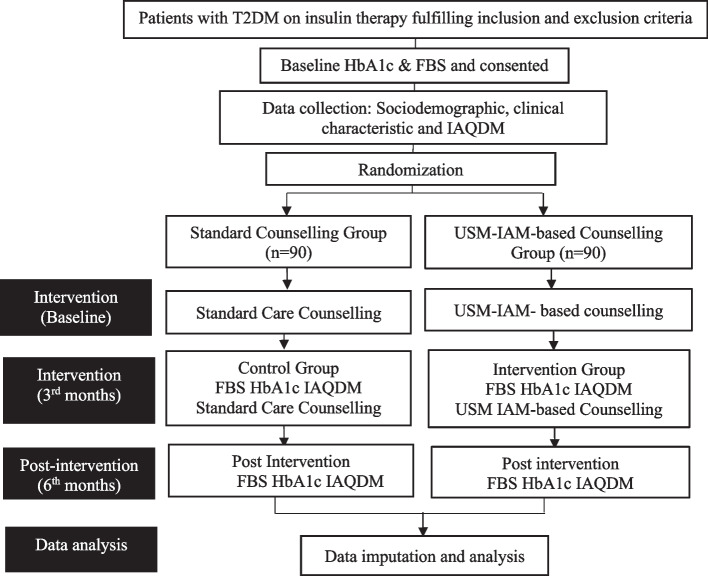
Study design

Like in the SCG, all participants received SMBG diaries with the same instructions. During the second visit (3 months later), blood was drawn, and adherence scores were recorded again. Participants received counselling once more, provided by the same educator. At the final visit (sixth month later), blood was drawn again, and adherence scores were recorded once more. The outcome measures included changes in the adherence score; and FBS and HbA1c levels from baseline to 3 months and from baseline to 6 months. Adherence scores were assessed using the IAQDM, HbA1c levels were measured using the capillary electrophoresis method, and FBS was measured using the glucose oxidase method. Figure 1 illustrates the study design.

### Data analysis

All the data were entered and analysed using the Statistical Package for Social Sciences (SPSS) version 28. A comparison of sociodemographic and clinical characteristics at baseline was made using chi-square tests for categorical data, and paired t-tests were utilised for normally distributed continuous data. As the study aimed to assess the effectiveness of the new education module on patients’ adherence and glycaemic parameters, the missing data were imputed, and intention-to-treat analysis was used [31]. Repeated-measures analysis of variance (RM ANOVA) was applied to compare the mean differences in IAQDM, FBS and HbA1c between the USM-IAM and SCG over time. The results were interpreted by the p- value of the F- test, followed by the estimated marginal means. Changes in adherence scores, FBS, and HbA1c levels were analysed between baseline and 6 months. Subgroup analysis was then performed by stratification based on age groups (0–59 or ≥ 60), gender, education level (up to secondary or tertiary: diploma through PhD), and duration of diabetes (< 10 years, ≥ 10 years) to provide insights into whether these factors impact the results differently. For all analyses, a *p*-value of < 0.05 is considered to be significant.

## Results

One hundred eighty participants were initially enrolled in the study and were randomised equally to both groups. The sociodemographic and clinical characteristics of the participants at baseline are shown in Tables 2, 3, and 4 respectively.

**Table 2:** Baseline sociodemographic data of the study participants

Variables		Frequency, n (%)		*p*-value^a^
	Overall (*n* = 180)	Standard Counselling Group (*n* = 90)	USM-IAM based Counselling Group (*n* = 90)	
Age, years old	56.1 (7.96)^b^	57.08 (7.92)^b^	55.16 (7.93)^b^	0.483^c^
Sex				
Female n (%)	96 (53.3)	46 (25.5)	50 (27.7)	0.550
Male n (%)	84 (46.7)	44 (24.4)	40 (22.2)	
Ethnicity				
Malay	171 (95)	84 (46.7)	87 (48.3)	0.305
Non-Malay	9 (5)	6 (3.3)	3 (1.7)	
Education Level				
Primary & secondary (%)	100	47 (26.1)	53 (29.4)	0.368
Tertiary (%)	(55.5) 80 (44.4)	43 (23.9)	37 (20.6)	
Marital status				
Single/divorced/widowed (%)	17 (9.5)	12 (6.7)	5 (2.8)	0.054
Married (%)	163 (90.5	78 (43.3)	85 (47.2)	
Occupation				
Working	86 (47.8)	40 (22.2)	46 (25.6)	0.423
Non-working	94 (52.2)	50 (27.8)	44 (24.4)	

^a^Chi-square test

^b^Mean (S.D. (Standard Deviation))

^c^Independent T-test

**Table 3:** Baseline comparison of clinical characteristics between the intervention and control groups

Variables	Frequency, n (%)			*p*-value^a^
	Overall (*n* = 180)	Standard Counselling Group (*n* = 90)	USM-IAM-based Counselling Group (*n* = 90)	
Family history of diabetes				
Yes	140 (77.8)	67	73	0.282
No	40 (22.2)	23	17	
Duration of diabetes (years)	13.85 (6.69)	13.73 (7.04)	13.97 (6.36)	0.606
Duration of insulin treatment (years)	7.29 (5.71)	7.04 (5.76)	7.48 (5.67)	0.864
Number of injections per day	2.95 (1.15)	2.93 (1.15)	2.97 (1.16)	0.440
Practicing SMBG				
Yes	142 (78.9)	73 (40.5)	69 (38.3)	0.465
No	38 (21.1)	17 (9.5)	21 (11.7)	
Regular exercise				
Yes	74 (41.1)	41 (22.78)	33 (18.33)	0.289
No	106 (58.9)	49 (27.22)	57 (31.7)	
Presence of comorbidities ^d^				
Hypertension	148 (82.2)	71 (39.4)	77 (42.8)	0.242
Dyslipidemia	152 (84.4)	77 (42.8)	75 (41.7)	0.681
Stroke	9 (5)	5 (2.8)	4 (2.2)	> 0.999 ^c^
Heart Disease	22 (12.2)	12 (6.7)	10 (5.6)	0.649
Gout	14 (7.8)	7 (3.9)	7 (3.9)	> 0.99
Body Mass Index	29.57 (4.82)	29.36 (4.60)	29.78 (5.01)	0.565
Adherence scores (%)	64.56 (11.54)	63.54 (11.33)	65.58 (11.71)	0.990
HbA1c (%)	10.56 (1.60)	10.31 (1.58)	10.81 (1.59)	0.697
FBS (mmol/L)	11.36(4.02)	10.72 (4.09)	12.00 (4.03)	0.563
Systolic blood pressure	144.5 (20.2)	144.04 (20.02)	144.98 (20.52)	0.758
Diastolic blood pressure	80.9 (9.98)	80.49(8.93)	81.3 (10.96)	0.587
Medication				
Insulin + OGLD/s	161 (89.4)	82 (45.6)	79 (43.9)	0.467
Insulin only	19 (10.6)	8 (4.4)	11 (6.11)	
Antihypertensive/s	157 (87.2)	75 (41.7)	82 (45.6)	0.118
Statin	151 (83.9)	73 (40.5)	78 (43.3)	0.311
Fenofibrate	16 (8.9)	7 (3.9)	9 (5)	0.600
Aspirin	11 (6.1)	6 (3.3)	5 (2.8)	0.756
Clopidogrel	18 (10)	9 (5)	9 (5)	> 0.99

^a^Chi-square test

^b^Independent T-test

^c^Fisher’s exact test

^d^multiple answers possible

**Table 4:** Changes in adherence score, FBS and HbA1c

		Standard Counselling Group (*n* = 90)		USM-IAM-based Counselling Group (*n* = 90)		Between subjects’ effect
Outcome	Time	Mean difference (95%CI)	p-value	Mean difference (95%CI)	*p*-value	*p*-value
Adherence score	0–3 months	−7.64 (− 10.89, − 4.40)	< 0.001 ^a^	−8.30 (− 11.47, − 5.14)	< 0.001^a^	0.159
	0–6 months	− 10.79 (− 14.64, − 6.97)	< 0.001 ^a^	−10.21 (− 13.40, − 7.03)	< 0.001^a^	
	3–6 months	−3.14 (− 6.766, 0.48)	0.110	−1.91 (− 5.19, 1.37)	0.480	
Fasting Blood Sugar	0–3 months	0.438 (− 0.66, 1.54)	> 0.095	1.374 (0.25, 2.50)	0.011^a^	0.115
	0–6 months	0.998 (− 0.02, 2.01)	0.056	1.713 (0.473, 2.95)	0.003 ^a^	
	3–6 months	0.561 (− 0.557, 1.68)	0.672	−0.339 (−1.545, 0.88)	> 0.95	
HbA1c	0–3 months	0.547 (0.12, 0.98)	0.008^a^	1.01 (0.67, 1.36)	< 0.01^a^	0.253
	0–6 months	0.617 (0.20, 1.03)	0.001^a^	1.03 (0.65, 1.41)	< 0.01^a^	
	3–6 months	0.070 (−0.32, 0.46)	> 0.95	0.02 (−0.31, 0.35)	> 0.95	

^a^Significant p-value

At baseline, the two groups were homogeneous concerning all the variables. The patients included in our study had a mean age of 56 years. One hundred-twenty-three (69%) of the patients were 54 years old or older. The patients have long-standing diabetes and a mean duration of insulin injection of 7 years. Even though most participants underwent SMBG: performed capillary blood glucose measurements at least once a week, their HbA1c levels were still extremely high. Less than half of the participants exercised regularly: exercising five times a week for 30 minutes [32] despite many being pensioners or housemakers. Many of the participants had comorbidities, e.g., hypertension and hyperlipidaemia. Systolic blood pressure readings were mostly not on achieved targets. Majority of the participants were on metformin-insulin combinations, antihypertensives and statins.

**Fig. 2:**
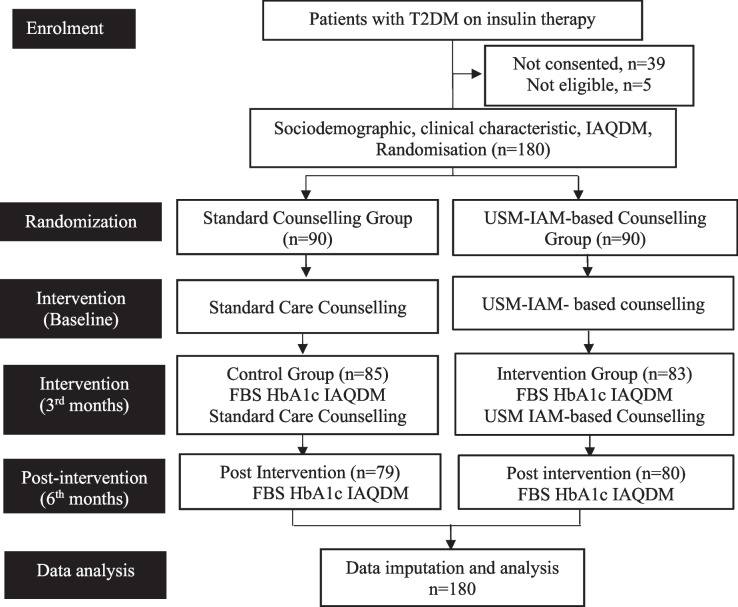
CONSORT diagram of the study participants

During enrolment, 39 patients declined to give consent, and five patients had an HbA1c level out of the inclusion range. During the study period, 21 (11.7%) participants were excluded (10 in the USM-IAM and 11 in the SCG (Fig. 2)).

The reasons for dropout were missed counselling sessions, blood sampling or completion of the adherence score. Complete case (per protocol) analysis was inadequate, and data imputation was needed. Missing data were imputed using the nonparametric missing value imputation [33] via the missForest method [34]. Imputation diagnosis was achieved by comparison of the data distributions by histograms. The imputation process retained the normal distribution shape of the variables.

**Fig. 3:**
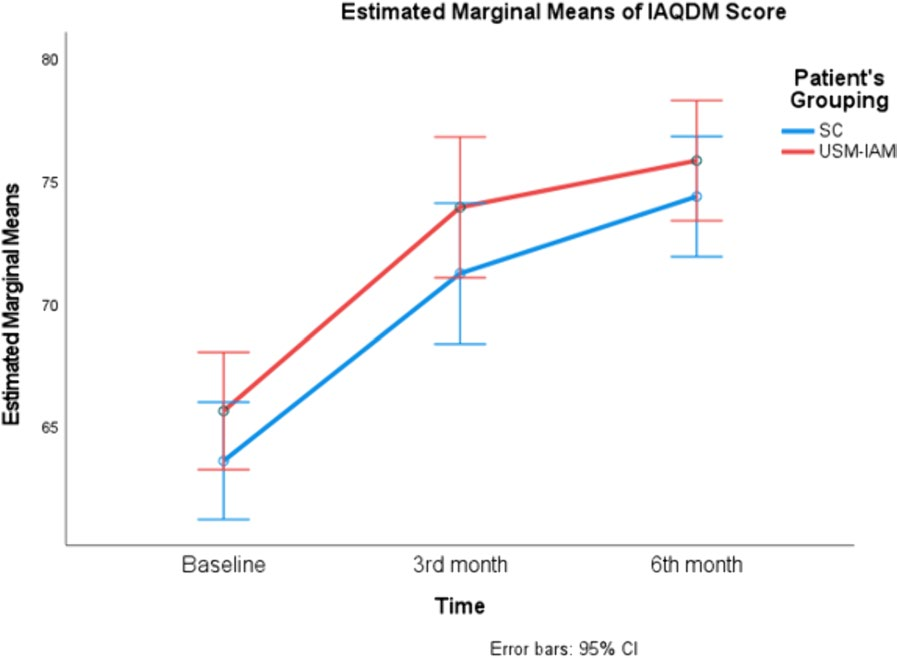
Describes changes in IAQDM score between two groups ( SC and USM-IAM) at baseline, 3 months and 6 months

**Fig. 4:**
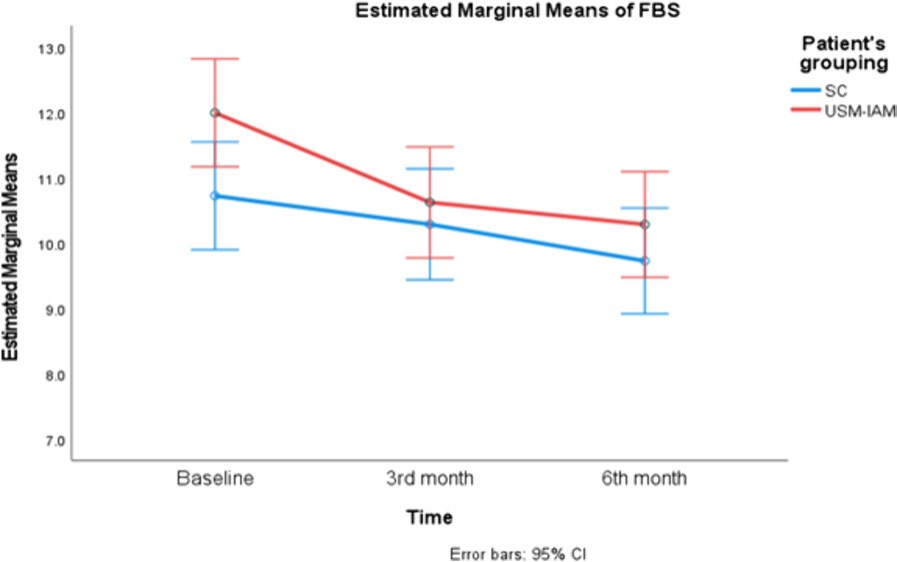
Describes changes in FBS between two groups ( SC and USM-IAM) at baseline, 3 months and 6 months

**Fig. 5:**
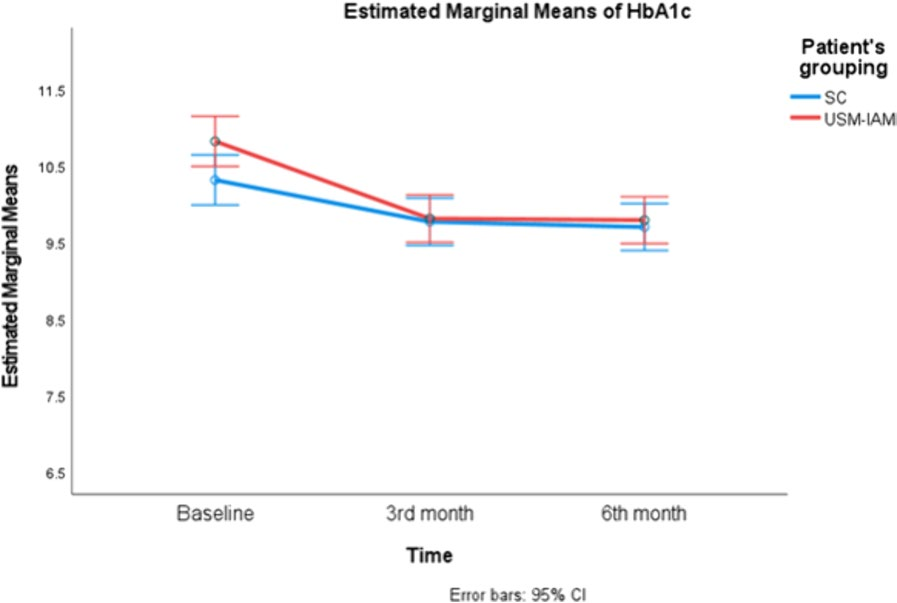
Describes changes in HbA1c between two groups ( SC and USM-IAM) at baseline, 3 months and 6 months

Both groups had significant improvements in adherence scores and HbA1c. There was a significant reduction in FBS in the USM-IAM but not in the SCG, as shown in Table 4. Figures 3, 4, and 5 show profile plots of the adherence score, FBS and HbA1c, respectively. The between-subjects effects for all outcomes were not significantly different, as both groups experienced improvements. Table 5.

**Table 5:** Sub-analysis of adherence score, FBS and HbA1c changes between baseline and 6 months with regard to categorized variables

Outcome	Parameter		Standard Counselling Group			USM-IAM-based Counselling Group			Treatment effect
			n	Mean difference	*p*-value	n	Mean difference	*p*-value	*p*-value
Adherence scores change	Age	< 60	49	− 8.20	**0.003**	52	−10.37	**< 0.001**	0.057
		≥ 60	41	−13.88	**< 0.001**	38	−10.00	**< 0.001**	0.780
	Sex	Male	44	−8.25	**0.003**	40	−14.07	**< 0.001**	0.828
		Female	46	−13.22	**< 0.001**	50	−7.13	**< 0.001**	0.065
	Education level	Secondary	47	−9.342	**< 0.001**	52	−10.66	**< 0.001**	0.555
		Tertiary	43	−12.36	**< 0.001**	38	−9.61	**< 0.001**	0.107
	Diabetes duration	< 10 years	50	−10.86	**< 0.001**	48	−10.31	**< 0.001**	0.813
		> 10 years	40	−10.70	**< 0.001**	42	−10.10	**< 0.001**	**0.035**
FBS change	Age	< 60	49	0.61	0.741	52	2.28	**0.003**	0.090
		≥ 60	41	1.47	0.106	38	0.94	0.721	0.608
	Sex	Male	44	0.81	0.362	40	1.23	0.241	0.094
		Female	46	1.18	0.237	50	2.10	**0.019**	0.593
	Education level	Secondary	47	0.124	> 0.99	52	1.55	0.118	0.372
		Tertiary	43	1.95	0.006	38	1.94	**0.021**	0.199
	Diabetes duration	< 10 years	50	0.67	0.648	48	1.40	0.120	0.193
		> 10 years	40	1.41	0.116	42	2.07	**0.036**	0.374
HbA1c change	Age	< 60	49	0.51	0.057	52	0.95	**< 0.001**	0.068
		≥ 60	41	0.75	0.005	38	1.14	**< 0.001**	0.651
	Sex	Male	44	0.71	0.059	40	0.82	0.005	0.131
		Female	46	0.53	0.057	50	1.21	**< 0.001**	0.925
	Education level	Secondary	47	0.61	**0.020**	52	0.99	**< 0.001**	0.644
		Tertiary	43	0.62	0.080	38	1.09	**< 0.001**	0.273
	Diabetes duration	< 10 years	50	0.78	0.008	48	1.04	**< 0.001**	0.363
		> 10 years	40	0.42	0.455	42	1.02	**< 0.001**	0.492

From the sub-analysis, there was better insulin adherence observed among patients who received the USM-IAM in patients with diabetes for more than 10 years, compared to shorter diabetes duration. Those with a longer duration of diabetes might have more experience with insulin therapy and improved adherence with additional information in the new module. However, due to a small sample size in the sub-group of patients, a more conclusive analysis cannot be made.

## Discussion and conclusion

The primary outcome of this study was the change in adherence score. In our study, increases in adherence scores were observed in both groups. As participants in both groups received counselling and were encouraged to titrate their insulin dosage based on their SMBG, they had to adhere to the prescribed insulin regimen to facilitate dosage adjustment. In addition, educators in the USM-IAM group discussed the relationship between insulin and diabetes, explored the causes of nonadherence and provided measures to overcome nonadherence. By understanding the causes of nonadherence and measures to overcome them, participants in the USM-IAM could promptly respond and demonstrate greater adherence to their insulin therapy, as reflected by the greater increase in adherence score. Other studies assessing the effect of education on adherence also showed improvements as measured by the Diabetes Management Self-Efficacy Scale (DMSES), the Summary of Diabetes Self-Care Activities (SDSCA) [35], and the MMAS-8 [36].

According to a study that utilised similar adherence tools to our study (IAQDM), only 8.4% of their patients had a score ≥ 80, indicating adherence. In this study, we found that 8.9% of participants achieved a score of ≥80 at baseline, a percentage that was almost similar to that in the aforementioned study [8.9% vs. 8.4% [30]]. Since the study by Nasruddin et al. was cross-sectional, post-intervention scores were not reported. In our study, the percentage of adherent patients among the 180 patients improved to 34.7 and 34.2% at 3 months and 6 months, respectively. Unfortunately, the mean adherence score in the study conducted in Klang (another state in Malaysia) was not reported, preventing a direct comparison.

The second outcome of the study was change in FBS. The USM-IAM group exhibited statistically significant reductions in FBS levels between baseline and the third month and between baseline and the sixth month. In contrast, the control group showed nonsignificant reductions. Guo et al. assessed the efficacy of structured education in patients with type 2 diabetes mellitus receiving insulin treatment (OPENING) in China. They also evaluated changes in FBS among their education and control groups. The study revealed a significant reduction in FBS from baseline to the fourth month in both groups [36]. The education group displayed a greater reduction in FBS than the control group (2.84 ± 3.46 vs 2.76 ± 3.59). It is worth noting that patients in the OPENING study, who had higher FBS changes than did those our study [1.71 ± 1.24 mmol/l (intervention) vs. 1.0 ± 1.02 mmol/l (control)], had double OGLDs and had not yet initiated insulin treatment. During the 4-month study, these patients began two daily injections of 30% soluble–70% isophane recombinant insulin, which, combined with education, significantly reduced the FBS levels.

In this study, both groups showed statistically significant reductions in HbA1c between baseline and the third month, and between baseline and the sixth month. The mean HbA1c reductions in the sixth month were 1.03 and 0.67% in the USM-IAM and SCG, respectively. Although greater HbA1c reductions were observed in the USM-IAM, the difference was not significant. Similar patterns were observed in earlier randomised trials, namely, the MEDIAS-2 ICT [37], OPENING [36] and Cani et al. studies [38]. The first two studies involved physician-led interventions, while the latter involved pharmacist-led interventions. Table 6 provides a summary of RCTs of education intervention for T2DM patients treated with insulin. It also provides education related to insulin and diabetes included in the education interventions.

**Table 6:** Summary of RCTs of education interventions for T2DM patients treated with insulin

Author	Duration & Frequency	Baseline inclusion criteria	Provider	Module content	HbA1c reduction	
					Control group	Intervention group
Hermanns et al., 2012	6 months, 10 education sessions.	T2DM, age 18–75 years, on OGLDs for at least 2 years, BMI 20–40, able to read and understand the German language.	Diabetes educators	**ACC (control group)** Insulin, insulin action, injection technique, SMBG technique, carbo and calorie counting, hypoglycemia, exercise, diabetes complication, basal insulin, hypertension, self BP monitoring, healthy eating, salt-restriction, smoking cessation **MEDIAS2-ICT (intervention group)** Cover all the above with the addition of perception of barriers to insulin treatment, attitudes regarding SMBG, physical activities and BP monitoring, insulin adjustment during illness and social aspect of insulin treatment	0.37%	0.63%
Guo et al., 2014	4 months 6 (Control Group), 7 sessions + 7 phone calls	T2DM, age > 18 years, HbA1c > 7.5%, on ≥2 OADs for ≥3 months, able and willing to accept structured education	Trained nurse	**Control Group** Insulin injections, SMBG, hypoglycemia, and self-management. **OPENING PROGRAMME** Taking medication, insulin injection technique, SMBG, healthy diet, physical activity, prevention of hypoglycemia, and prevention of complications	2.08%	2.16%
Cani et al., 2015	6 months 6 sessions for the intervention group	Age ≥ 45, T2DM on prescription insulin, HbA1c > 8%	Pharmacist	**Control Group** No counselling. **Clinical Pharmacy Programme** Pharmacotherapeutic Care Plan: indication, proper dosage, side effects and adequate storage of medication. Pill organisers and written guidance on prescriptions were given. Diabetes education protocol: acute and chronic complications, the importance of lifestyle changes (healthy diet, physical activity, smoking cessation), regular foot inspections, the importance of home blood glucose monitoring and other topics.	0.08%	0.57%

There are notable differences between our study and the previously mentioned studies. In the OPENING study, patients experienced greatest decrease in HbA1c, exceeding 2% for both groups. Patients who did not self-inject throughout the 4-month study were excluded. The initiation of insulin significantly reduced HbA1c levels, complemented by intensive education, including six face-to-face education sessions and three telephone follow-ups.

In Cani et al.’s study, both the control and intervention groups showed HbA1c reductions, with greater reductions observed in the intervention group (0.57% vs 0.08%). Notably, the intervention group received six monthly (six) individual education sessions, while the control group received no education sessions, and was followed up only at the beginning and end of the study. It is important to highlight that Cani et al.’s study differs from ours, as the comparator group in our study received standard counselling.

Participants recruited for our study had been treated with insulin and had HbA1c values exceeding 8%, similar to those in the MEDIAS-2 ICT study. Despite our patients receiving only two education sessions by the diabetic educators compared to the 10 education sessions in the MEDIAS-2 ICT, participants in our study demonstrated better HbA1c reductions than did those in the MEDIAS-2 ICT. Specifically, the intervention group in our study showed a reduction in HbA1c of 1.03% as compared to MEDIAS-2 ICT’s 0.63%, while the control group in our study had a reduction of 0.59% compared to MEDIAS-2 ICT’s 0.37%.

Notably, our study was conducted immediately after the lifting of the movement control order (MCO) in response to the COVID-19 pandemic in Malaysia, during which time specialist clinics started to resume their services back towards pre-pandemic levels. During the pandemic, patients who visited specialist clinics for follow-up only received prescriptions without consulting a doctor, a practice adopted globally to conserve resources and minimise the risk of COVID-19 transmission [39]. These patients face various challenges, such as limited income, reduced access to needles and glucometer strips and restrictions on consultations with doctors and dieticians [40]. When the pandemic was under control and life returned to normal, patients became more motivated to improve their diabetic control.

In our study, participants received individual counselling, which differed from the group counselling practised in the MEDIAS-2 ICT study. Individual counselling allows participants better opportunities to ask questions and clarify doubts. The 10 lessons covered in the interventions of the MEDIAS-2 ICT study might be redundant for patients, and the limited chance of asking questions could have reduced their motivation and made them feel burdened.

In addition to these factors, the module content in our study differed from the counselling given in the other three comparison studies. While our intervention group’s counselling covered all the topics in the three studies, our module also included additional information and discussion on the relationship between insulin and diabetes, the definition of nonadherence, the common causes of nonadherence and their suggested solutions, and how to fast safely with insulin therapy.

In the module of the current study, the section on the relationship between insulin and diabetes explained the physiological role of insulin in the human body. Insulin functions as a key to opening the door for blood glucose to enter body cells. This process reduces blood glucose levels, allowing body cells to utilise glucose as fuel for energy production. Participants were also educated about the different types of insulin, including prandial and basal insulin, and their respective functions. This information is crucial for enhancing participants’ understanding of the importance of insulin usage, which can lead to improved adherence.

The definition of insulin nonadherence in the module included not injecting as scheduled, not optimising the insulin dose, changing the dosage, or altering the frequency of insulin injections without consulting the treating doctor. The causes of nonadherence and their suggested solutions, gathered from focus group discussions, served as guidance for participants on how to address potential problems they faced with insulin treatment. In the section on fasting safely with insulin therapy, participants were informed about potential complications that can occur during fasting and about the timing of blood sugar monitoring and indications that necessitate breaking the fast.

Compared to the other three studies discussed earlier, none of them covered the definition of insulin nonadherence, the causes of insulin nonadherence, and measures to overcome nonadherence. These new topics provided additional valuable information to participants, especially those who received insulin injections as part of their medication regimen. This new information could be used in counselling patients with T2DM to increase their insulin adherence.

In our study, we noticed significant improvements occurring between 0 and 3 months and 0–6 months. However, the changes between 3- and 6 months were not significant for any of the three outcomes. This indicates that only minimal improvements in adherence, FBS, and HbA1c occurred between the third and sixth months. The similarity in education content during the second visit, which mirrored the content at recruitment, limited the changes participants could make within 3 months. Therefore, a different counselling content (dynamic intervention) may be necessary to enhance adherence and achieve further HbA1c reduction.

This study has several limitations, notably, it was conducted at a single centre. Our findings relied on self-reports, introducing the possibility of recall bias and patients responded to please the researchers. These biases can lead to underestimation or overestimation of adherence. However, self-report questionnaires are widely used for measuring adherence due to their cost-effectiveness and time efficiency. Although they offer precise and reasonable estimates of adherence [41, 42], we recommend combining self-administered questionnaires with pharmacological monitoring for a more precise measurement of insulin adherence.

In conclusion, our findings indicate poor adherence to insulin therapy among participants, which improved after undergoing education module intervention. This was evidenced by reductions in HbA1c and FBS levels, along with an increase in the insulin adherence score. The counselling sessions had significant impacts on both groups, with USM-IAM showing a better effect than the standard counselling.

### Supplementary Information

**Supplementary Material 1.**

**Supplementary Material 2.**

**Supplementary Material 3.**

**Supplementary Material 4.**

## Data Availability

The study protocols for this research are accessible via ClinicalTrials.gov, with the ID number NCT05125185. The data that support this study are available from Hospital Universiti Sains Malaysia, but restrictions apply to the availability of these data. Data are, however, available from the authors upon reasonable request and with permission of Hospital Universiti Sains Malaysia.

## Abbreviations

BMI: Body mass index
CVI: Content validity index
DMSES: Diabetes Management Self-Efficacy Scale
FBS: Fasting blood sugar
HbA1c: Glycated hemoglobin
IAQDM: Insulin Adherence Questionnaire for patient with Diabetes Mellitus
IDF: International Diabetes Federation
MMAS-8: Morisky Medication Adherence Scale-8
OGLDs: Oral glucose-lowering drugs
RM ANOVA: Repeated-measures analysis of variance
SC: Standard counselling
SCG: Standard counselling group
SDSCA: Summary of Diabetes Self-Care Activities
SMBG: Self-monitoring of blood sugar
T2DM: Type 2 diabetes mellitus
USM-IAM: USM-IAM-based counselling/ Universiti Sains Malaysia Insulin Adherence Module
