# Supplementary material for: The effect of USM-IAM-based counselling vs standard counselling on insulin adherence, FBS and HbA1c among patients with uncontrolled type 2 diabetes mellitus (T2DM): a randomised controlled trial
Source: BMC Endocr Disord. 2024 Jul 18;24:118. doi: 10.1186/s12902-024-01577-6 (PMC11256455; doi:10.1186/s12902-024-01577-6)
Supplement: Supplementary file 1 — Supplementary Material 1. [file 12902_2024_1577_MOESM1_ESM.pdf]

INSULIN ADHERENCE MODULE FOR PATIENTS WITH TYPE 2 DIABETES

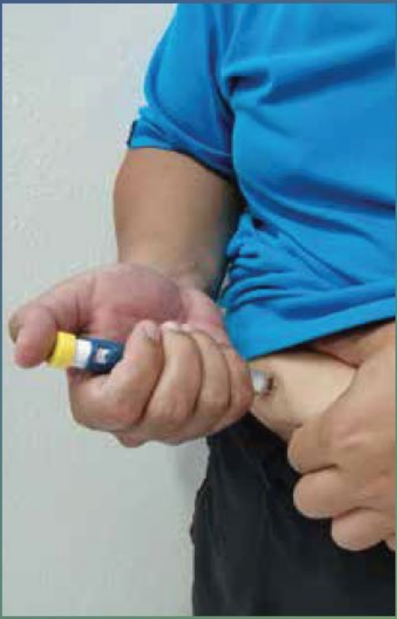

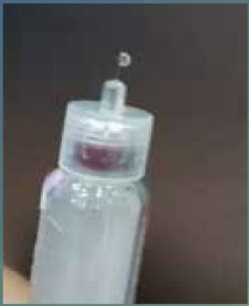

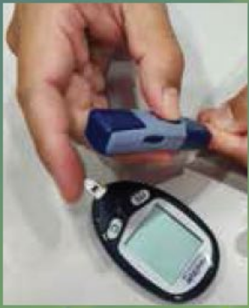

Aida Maziha Zainudin
Wan Mohd Izani Wan Mohamed
Rosediani Muhamad
Aida Hanum Ghulam Rasool
Mohd Zarawi Mat Nor
Najib Majdi Yaacob

© Universiti Sains Malaysia, 2021
All rights reserved.
No part of this document may be reproduced, stored or transmitted in any form or by any means (electronic, mechanical, photocopying, recording, etc.) without the permission of the copyright owner.

Table of Content

Introduction ii

Acknowledgement iii

UNIT 1 Diabetes and Insulin 1

1.1 Diabetes definition 1

1.2 Types of diabetes 1

1.3 What is the relationship between diabetes and insulin? 2

1.4 Types of Insulin 3

1.5 Why do patients with diabetes inject different insulins? 5

1.6 What insulin regimens are available? 5

UNIT 2 Nonadherence to insulin treatment and its consequences 6

2.1 What does nonadherence to insulin treatment mean? 6

2.2 What are the effects of nonadherence to insulin treatment? 6

UNIT 3 Causes of insulin nonadherence and how to overcome it 7

3.1 Insulin adverse effects 7

3.1.1 Hypoglycemia 7

3.1.2 Weight gain 8

3.1.3 Allergy to Insulin 9

3.2 Problems with insulin injection 9

3.2.1 Pain at the injection site 9

3.2.2 Insulin is less effective when injected in abnormal fat cells..9

3.2.3 Bleeding or bruising 10

3.2.4 Embarrassed to inject in public 10

3.3 Negative attitude toward insulin 10

3.3.1 Insulin injection is bothersome .... .10

3.3.2 Insulin injection interferes with my working time 10

3.3.3 Forgot to inject 10

3.3.4 Forgot to bring an insulin pen to the workplace 10
3.3.5 High treatment cost.. 11
3.4 Misperception of insulin 11
3.4.1 Insulin is not effective 11
3.4.2 Sugar levels remain high despite injecting insulin 11
3.4.3 No need to inject insulin while fasting 12
3.4.4 Forbidden to carry insulin when travelling on the airplane 12
3.4.5 Myths about Insulin.. 13
UNIT 4 Empowering diabetes self-care 14
4.1 Be disciplined in controlling diabetes 14
4.2 Do self-monitoring of blood sugar 15
4.3 Modify your insulin dose based on blood sugar level 16
UNIT 5 Fasting safely despite injecting insulin 20
5.1 How to modify insulin dose when fasting? 20
5.2 When do I need to monitor my blood sugar level when fasting?. 21
5.3 When should I break my fast?..21
References 22
APPENDIX A: Types of insulin pens available in the market 23
APPENDIX B: Carbohydrate exchange 24
APPENDIX C: Examples of moderate aerobic exercise 26
APPENDIX D: Areas of the body suitable for injecting insulin....................27
APPENDIX E: Insulin Injection Technique 28
APPENDIX F: Modification of insulin dosage 34
APPENDIX G: Steps to check your blood sugar level 35

Introduction

Objective
The purpose of this module is to improve the understanding, awareness and adherence of patients with diabetes to insulin treatment. This module contains 5 units namely:

- Unit 1: Diabetes and its relation to insulin
- Unit 2: Non-adherence to insulin treatment and its effects
- Unit 3: Causes of non-adherence to insulin treatment and how to overcome it
- Unit 4: Empowering diabetes self-care
- Unit 5: Fasting safely despite injecting insulin

Activities
You are asked to read and understand this module within 50 minutes. The time allocation for the sections is as follows:

- Unit 1: 10 minutes
- Unit 2: 5 minutes
- Unit 3: 15 minutes
- Unit 4: 15 minutes
- Unit 5: 5 minutes

After 50 minutes, you will see the doctor for 10 minutes for a question-and-answer session

Acknowledgement

First of all, I would like to thank all individuals who have guided me, contributed ideas and helped me in completing this module.

A word of appreciation to all the panel of assessors consisting of experts in their respective fields who have evaluated and given comments to improve the content of the module.

Thank you to patients who have rated and commented to make this module easy to read and understand.

Thanks also to the graphic designer, En Khairul Zahari, who edited the pictures, charts, illustrations and module cover.

Appreciation to Universiti Sains Malaysia for providing a short-term research grant (No: 304/PPSP/6315140), which contributed to the production of this module.

Thank you to everyone who was indirectly involved in the production of this module.

UNIT 1 Diabetes and Insulin

Objective: To provide information about diabetes and its relationship with insulin.
Activity: To read and understand Unit 1
Duration: 10 minutes

1.1 Diabetes definition

Diabetes is a condition where the function of the pancreas to produce insulin has been damaged/decreased or body tissues are insensitive to insulin. This causes excessive sugar levels in the blood. Patients may or may not experience symptoms[1]:

- ≥7 mmol/L after 8 to 10 hours of fasting and/or
- ≥ 11.1 mmol/L after two hours of glucose challenge
- HbA1c* ≥ 6.3 %

*HbA1c is the average blood sugar level for 3 months attached to red blood cells

1.2 Types of Diabetes

Generally, diabetes is categorized into three main types.

Type 1	Type 2	Gestational Diabetes
• Insulin is not produced by the pancreas because the pancreatic cells are damaged<br>• Usually occurs in children or adolescents	• Insulin production is not enough<br>• Body cells are not sensitive to insulin<br>• Usually occurs among those who are overweight	• Gestational diabetes that resolves after the mother gives birth<br>• Mothers are at high risk of developing type 2 diabetes if they do not control their diet

Figure 1: Types of diabetes

1.3 What is the relationship between diabetes and insulin?

Insulin functions like a key, unlocking sugar channels on the cell surface to enable sugar entry into the cell to produce energy.

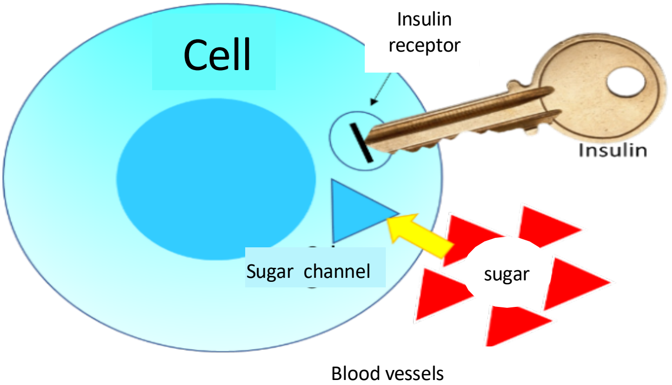

Figure 2: Insulin as a key for sugar to enter cells

Without insulin, sugar cannot enter cells, and causes elevated sugar levels in the blood vessels.

A healthy pancreas consistently releases basal insulin including during sleep to maintain normal blood glucose levels. A normal pancreas also responds by releasing insulin during meals (prandial insulin). Therefore, the sugar level will decrease within 2 hours after eating.

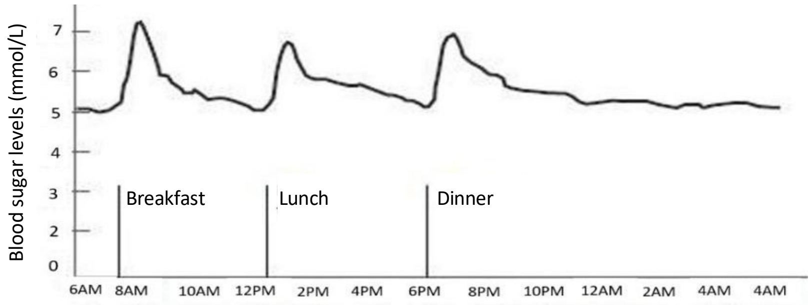

Chart 1: Sugar levels in normal individuals

Whereas, for you, your pancreas no longer produces enough insulin or the body's cells are not sensitive to insulin.

- Lack of **basal** insulin causes your blood sugar levels to be high as soon as you **wake up**.
- Lack of **prandial** insulin causes your blood sugar levels to remain high (red line) **after meals.**

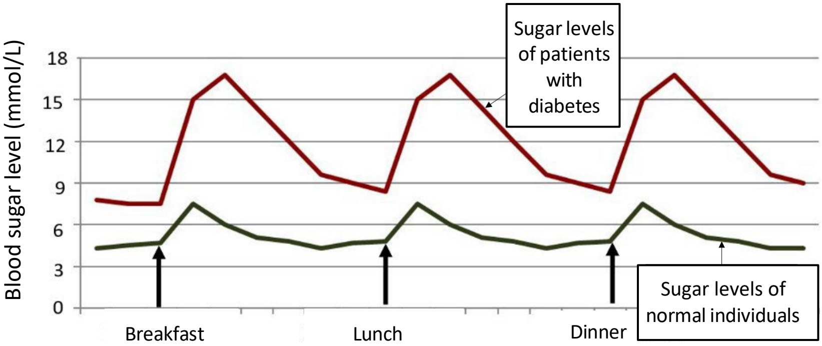

Chart 1: Sugar levels of normal individuals and patients with diabetes

1.4 Types of Insulin

Generally, insulin is divided into three types which are:

- **Basal:** lowers sugar for a long period (16-24 hours)
- **Prandial:** lowers sugar immediately after eating
- **Premixed**: a mixture of basal and prandial

Refer to APPENDIX A for the types of insulin pens available in the market

Once injected into the body, insulin takes a varying amount of time to begin lowering blood sugar. The duration of action also varies according to the type of insulin. Therefore, you need to plan the injection time before meals based on the type of insulin used as summarized in table 1.

Table 1: Insulin types

Insulin types	Subtypes	Insulin types	Onset of action	Duration of action (hours)	Injection time before meals (minutes)
**Basal Insulin**	Moderate	Insulatard® Insugen®-R	1.5 hour	18-23	Before bed/ at the same time every day
		Humulin N® Insugen®-N	1 hour	16-18	
	Long acting	Lantus® (Glargine)	2-4 hours	20-24	
		Levemir® (Determir)	1 hour	17-23	
**Prandial Insulin**	Rapid	Novorapid® (Aspart)	10-20 min	3-5	0-15 / after meal
		Humalog® (Lispro)	0- 15 min	3.5- 4.5	
		Apidra® (Glulisine)	5- 15 min	3.5	
	Fast	Actrapid®	30 min	8	30
		Humulin R®	30 min	6-8	
**Pre-mixed**	Rapid	NovoMix® 30	10-20 min	16-18	0-15
		Humalog Max® 25/75	0-15 min	16-18	
	Fast	Mixtard® 30	30 min	18-23	30
		Humulin® 30/70	30 min	18-23	

Chart 2 shows the start time and duration of insulin action

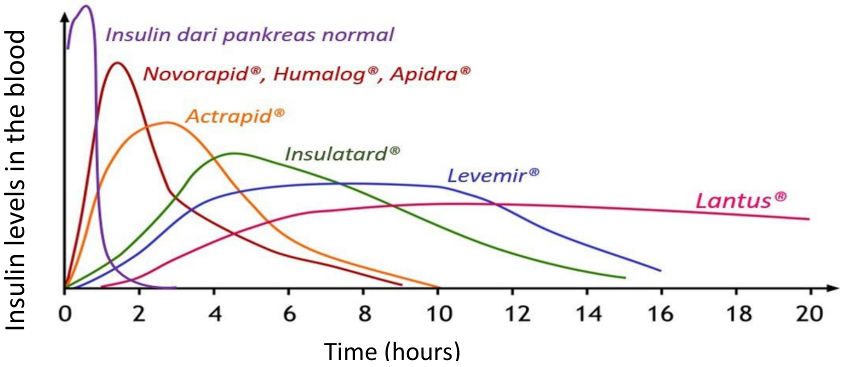

Chart 2: Time of onset and duration of insulin action

1.5 Why do patients with diabetes inject different insulins?

Patients with diabetes require different insulins based on blood sugar levels that are not within the target range. Some patients may only need basal insulin. Some patients require basal and prandial insulin. You should consult a doctor for insulin treatment as you may need a different type of insulin and insulin regimen* than other patients.

*Regimen means the type and number of insulin injections used

1.6 What insulin regimens are available?

- Basal injection only
- Basal injection with 1 prandial injection
- Basal injection with 2 prandial injections
- Basal injection with 3 prandial injections (basal-bolus)
- Pre-mixed injection twice daily
- Pre-mixed injection thrice daily

UNIT 2 Nonadherence to insulin treatment and its consequences

Objective: To improve the knowledge and understanding about nonadherence to insulin treatment and its consequences.
Activity: To read and understand Unit 2.
Duration: 5 minutes.

2.1 What does nonadherence to insulin treatment mean?

Nonadherence to insulin treatment means that you do not take injections, change the number of injections or change the dose of insulin used without consulting or receiving approval from a doctor [2].

2.2 What are the effects of nonadherence to insulin treatment?

When you fail to adhere to insulin injections, the sugar produced from the food you consume cannot enter the cells. Sugar remains in the blood vessels causing elevated blood sugar levels. As a result, you will urinate frequently, increase thirst and lose weight. The excess sugar will damage blood vessels, kidney cells and nerve cells leading to long-term complications such as:

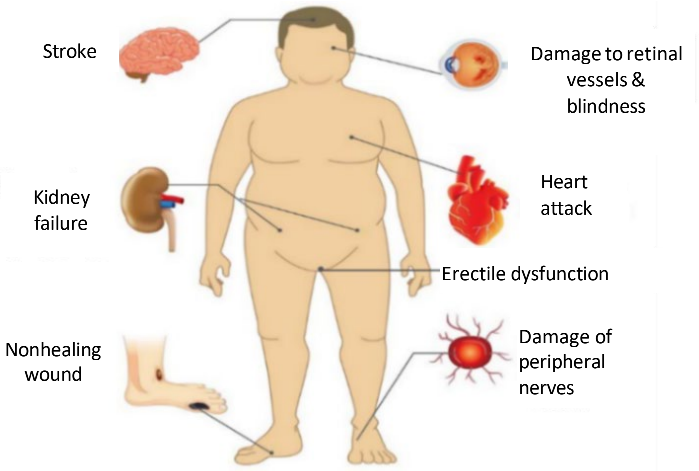

Figure 2: The effects of nonadherence to insulin treatment

UNIT 3 Causes of insulin nonadherence and how to overcome it

Objective: To improve the knowledge and understanding about the causes of nonadherence to insulin injection and how to overcome it.
Activity: To read and understand Unit 3.
Duration: 15 minutes.
There are many reasons why patients with diabetes do not adhere to insulin treatment. Among the causes of nonadherence with insulin treatment are:

3.1 Insulin Adverse effects

3.1.1 Hypoglycaemia

Hypoglycaemia is a condition where the blood sugar level is too low[3] i.e. less than 4.0 mmol/L and the patient may experience symptoms such as hunger, tiredness, palpitations, sweating, dizziness, headache, nausea, shivering, anxiety, irritability, nightmares, fainting/blackout.[4] There are a few patients who have experienced hypoglycaemia and are afraid to continue injection/ reducing the dose of insulin without monitoring their sugar levels.

Table 2: Causes of hypoglycaemia and how to overcome it

Causes of hypoglycaemia	How to overcome it
Eating less than usual<br>OR<br>Injecting prandial insulin and not eating	Eat according to the schedule and eat in the same quantity according to certain mealtimes. Nutritional drinks such as Nutren/ Glucerna can be taken to replace food when time is short/ lack of appetite (fixed carbohydrate content)
Eating late after injecting prandial insulin	Immediately eat at the onset of prandial insulin action.
Excessive insulin dose	Monitor blood sugar levels and reduce

	insulin dose if hypoglycaemia occurs
Excessive exercise	Take 15-30g of carbohydrates if the sugar level is less than 5.6 mmol/L before exercise[5]
Alcohol intake	Stop drinking alcohol

If **hypoglycaemia** occurs, you need to take sugary foods/drinks in a specific proportion based on sugar level[3].

- Sugar level 3.3 -3.9 mmol/L: 3 tablespoons of white sugar or 2 candies or half a cup (120 ml) of sugary fruit juice.
- Sugar level 2.5 – 3.2 mmol/L: 4 tablespoons of white sugar or 3 candies or ¾ cup (180ml) of sugary fruit juice.
- Sugar level <2.5 mmol/L: 6 tablespoons of white sugar or 5 candies or 1 cup (240ml) of sugary fruit juice.
- Unconscious patient: Immediately take the patient to a health clinic/hospital for intravenous sugar treatment.

3.1.2 Weight gain

Insulin injections cause blood sugar levels to drop, causing hunger and eating between meals. So, the patient consumes more calories than the body needs. Another cause of weight gain is due to:

- Good sugar control reduces the sugar loss in the urine. Therefore, more calories are stored in the body.
- Insulin increases lean body mass through its anabolic properties.
- Insulin can cause water and salt to be stored in the body.
- Lack of exercise.

To avoid weight gain:

- Identify the cause of hypoglycaemia and take preventive measures.
- Limit carbohydrate intake to 11-12 servings a day (i.e. 2 servings of fruit, 1 serving of milk, 1 serving of sugar and 7-8 servings of grains). (Refer to APPENDIX B)

- Engage with moderate intensity aerobic exercise for 150 minutes a week. (See APPENDIX C).

3.1.3 Allergy to insulin

Allergy to insulin is rare. After purified insulin was introduced, only 1 in 1000 people developed an allergy. Signs of an allergy are redness and itching at the injection site. If the redness and itching spread to the whole body, or the lips are swollen and it is difficult to breathe, you need to stop the injection and get treatment in the hospital.

3.2 Problems with insulin injection

3.2.1 Pain at the injection site

Table 3: Causes of pain at the injection site and how to overcome pain.

Causes of pain at the injection site	How to overcome it
Needles that are dull from repeated use	Each needle is used only once*
Injecting in the same area repeatedly	Injecting insulin at different sites with proper technique (Refer to Appendix D & E)
Inject into the scarred area	Avoid injecting insulin in scarred areas

*If you have financial constraints, the maximum use of the needle is three (3) times only.

3.2.2 Insulin is less effective when injected into an area with abnormal fat cells

Excessive fat cells (lipohypertrophy) or reduced fat cells (lipoatrophy) under the skin can occur if insulin injection done at the same place repeatedly. This may also cause insulin to be less effective. You need to inject insulin at different places with the proper technique.

3.2.3 Bleeding or bruising

Bleeding/ bruising occurs if you are injected into blood capillaries under the skin. To avoid this, inject insulin with the correct technique and needle size.

3.2.4 Feeling embarrassed to inject in public

The injection area is "aurat"; a prohibited body area to be exposed to other for Muslim female patients. A decent man will also not reveal his body area in front of the audience. You can inject insulin in the vehicle or find a suitable closed place.

3.3 Negative attitude toward insulin

3.3.1 Insulin injection is bothersome

Some patients find injecting insulin troublesome. If you understand that insulin injections are necessary for your health, you will try to make them easy and convenient.

3.3.2 Insulin injection interferes with my working time

Insulin injections can interfere with your work. If insulin interferes with working hours, you should consult your doctor on a suitable appropriate insulin regimen.

3.3.3 Forgot to inject

- Make reminders on your phone, or
- Set an alarm or
- Ask your relative to remind you, AND
- Always remind yourself that you need something before eating to keep you healthy.

3.3.4 Forgot to bring an insulin pen to the workplace

To prevent you from forgetting to bring an insulin pen to work:

- Put the insulin pen in the work bag immediately after the last injection at home, or
- Reserve an insulin pen that is used at work (if you can afford it).

3.3.5 High treatment cost

You need to allocate some money to buy insulin needles, alcohol wipes and blood sugar monitoring strips. Do joint any diabetes support group. Most diabetes support groups sell needles and glucose strips at a discounted price for members. Examples of support groups you can join, and association information are as follows:

Table 4: Diabetes support group

	Support groups name	Address	Phone number
1	Malaysian Diabetes Association USM branch	Klinik Pakar Perubatan, Hospital USM	097673564
2	Malaysian Diabetes Association HRPZII branch	Kompleks Rawatan Harian HRPZ II	09- 745 2000 and ask the operator to connect to the Diabetes Resource Centre
3	Association of diabetes in regional hospitals and health clinics	Please contact the nurse or staff involved with diabetes care at the health facility	

3.4 Misperception of insulin

3.4.1 Insulin is not effective

There are a few patients who think that insulin is not effective in lowering sugar levels. You need to monitor your sugar level and increase the dose of insulin until your sugar level reaches the target.

3.4.2 Sugar levels remain high despite injecting insulin

Table 5: Causes of high blood sugar despite injecting insulin and how to overcome it

Causes why sugar remains high	Ways to overcome
Your body is not sensitive to insulin	Increase physical activity to increase insulin sensitivity

Insufficient insulin dose	Monitor blood sugar levels and increase the dose of insulin until the blood sugar level reaches the target
You are sick/stressed and your body releases hormones that increase sugar levels	Monitor sugar levels during illness and adjust insulin dose based on your sugar levels (Refer to APPENDIX F)
Insulin has been damaged by exposure to extreme heat	Store unopened insulin pens at 2-8°C* and opened pens at 15-27°C. Do not expose the insulin pen to extreme temperatures.
Insulin is deactivated because it has been frozen and thawed again	Do not store insulin pens in the freezer
Past the expiration date	Check the expiration date before injecting insulin

* Store the insulin in a refrigerator near the cooler. Do not store in the refrigerator door because the temperature is not constant.

3.4.3 No need to inject insulin while fasting

Only the insulin injected before lunch does not need to be injected while fasting. Other injections should be continued with appropriate dose modifications (Please refer to Unit 5).

3.4.4 It is forbidden to carry insulin when travelling on the airplane

You can take insulin with you abroad. To ensure that your insulin passes security check at the airport, carry a sufficient supply of insulin for the duration of the visit in its original packaging and obtain a doctor's letter confirming that you are carrying insulin for diabetes treatment.

3.4.5 Myths about Insulin

Table 6: Myths about insulin

Myths	Facts
**Insulin is made from porcine**	Now, no more insulin is made from porcine sources. Human insulin DNA* is injected into bacterial DNA and grown in a fermentation tank. Once the bacteria have grown, the insulin is harvested and purified and made available for medical use[6].
**Insulin causes kidney failure**	Insulin does not damage the kidneys. The main cause of kidney damage is caused by high and persistent sugar levels over a long period.
**When using insulin, my diabetes cannot be treated anymore**	Injecting insulin does not mean that you have reached the worst stage of diabetes and can no longer be treated.<br>When the doctor diagnoses you with diabetes, 50% of your pancreatic cells have been damaged and do not produce insulin7. Pancreatic function will continue to decrease over time. After 5-10 years of diabetes, most patients no longer produce insulin and need to inject insulin to control sugar levels.<br>Many patients are able to reach their target sugar levels by injecting insulin. This can avoid diabetes complications compared to patients who fail to control sugar levels with food and refuse to inject insulin.

*DNA - deoxyribonucleic acid (the main structure in the chromosome that carries genetic information)

UNIT 4 Empowering diabetes self-care

Objective:
1. To increase knowledge and understanding about sugar control targets, blood pressure, cholesterol and ideal body weight.
2. To increase the patient's motivation to monitor sugar levels and modify their insulin doses.

Activity: To read and understand Unit 4.

Duration: 10 minutes.

4.1 Be disciplined in controlling diabetes

You cannot expect a doctor to control your diabetes. You are responsible for taking care of yourself!

If you do not want to spend the end of your life with complications of diabetes such as dialysis, stroke, blindness, disability and so on, you have to do it yourself.

How to do it?

- Be disciplined with your mealtimes
- Choose your food
- Exercise 150 minutes a week
- Self-monitor the sugar level and change the insulin dose until it reaches the target sugar level
- Knows control targets and strives to achieve them

Table 7: Control targets[1]

	Parameters	Targets
A	HbA1c (3-month average sugar)	
	Just diagnosed with diabetes, no complications, long life expectancy, less risk of hypoglycaemia	6.0- 6.5%
	The others	6.6 -7.0 %
	There is heart, kidney/liver complications, short life expectancy, risk of hypoglycaemia	7.1 – 8.0 %

**B**	Blood pressure	<135/75 mmHg
**C**	Cholesterol (in mmol/L units)	HDL-C > 1.0 for male<br>> 1.2 for female<br>LDL-C < 2.6<br>Triglyceride <1.7
	Body weight	If overweight, aim for a 5-10% weight loss within 6 months

4.2 Do self-monitoring of blood sugar

Self-monitoring of blood sugar means measurement of sugar level at home using a "glucometer" (sugar level measuring device) without the presence of a doctor or nurse. It is done to monitor changes in blood sugar levels in response to your diet, physical activity and insulin dosage used. Studies have shown that patients who do it regularly have better control of their blood sugar levels than those who do not[8].

Ideally, you are encouraged to monitor your sugar level each time before injecting insulin. Target sugar levels are as follows:

Table 8: Targets of blood sugar

Fasting	4.4 – 7.0 mmol/L
2 hours post-meal	4.4 – 8.5 mmol/L

Please refer to APPENDIX G for steps for checking blood sugar levels.

4.3 Modify your insulin dose based on blood sugar level

Ideally, you are encouraged to monitor your sugar level each time before injecting insulin.

- If your sugar level exceeds the target three times in a row, you need to increase the dose of insulin by 2 units. Add 2 units ONLY if 3 readings exceed the target.
- If the sugar level is less than the target level or you experience hypoglycaemia, you need to reduce the insulin dose by 2 units.
- Maintain the insulin dose if your sugar level is in the target range.

For example, if you inject 12 units of **basal** insulin before going to bed, you need to monitor the sugar level before breakfast. If the reading exceeds 7.0 mmol/L 3 times, you need to increase the insulin dose to 14 units.

Table 9: Example of insulin dose modification for the ***basal*** *regimen*

Date*	Blood sugar level before breakfast (mmol/L)	Pre-bed insulin dose (unit)
1.6.20		12
2.6.20	7.2	12
3.6.20	7.8	12
4.6.20	8.5	**14**

+2 units

You don't have to check your blood sugar every day. For example, you can do it* ***3 times a week**. If all three readings are above the normal range,* ***increase*** *the dose of insulin* ***every week*** *until the sugar level reaches the target level.*

If you inject **pre-mixed insulin 2 times a day** (before breakfast and before dinner), you need to monitor the blood sugar level twice:

- If the sugar level exceeds 7.0 mmol/L before breakfast 3 times, add 2 units of insulin dose before dinner
- If the sugar level exceeds 7.0 mmol/L before dinner 3 times, add 2 units of insulin dose before breakfast.

Table 10: Example of insulin dose modification for a twice-daily pre-mixed regimen

Date	Blood sugar level pre-breakfast (mmol/L)	Pre-breakfast insulin dose (unit)	Blood sugar level pre-dinner (mmol/L)	Pre-dinner insulin dose (unit)
1.6.20				12
2.6.20	7.2			12
3.6.20	7.8			12
4.6.20	8.5			**14** (+2 units)
5.6.20		12	8.7	
6.6.20		12	7.5	
7.6.20		12	7.2	
8.6.20		**14** (+2 units)		

If you inject bolus basal insulin (4 times a day), you need to monitor your sugar level 4 times a day:

- If the sugar level exceeds 7.0 mmol/L before breakfast 3 times, add 2 units of insulin dose before going to bed.
- If the sugar level exceeds 7.0 mmol/L before lunch 3 times, add 2 units of insulin dose before breakfast.
- If the sugar level exceeds 7.0 mmol/L before dinner 3 times, add 2 units of insulin dose before lunch.
- If the sugar level exceeds 7.0 mmol/L before going to bed 3 times, add 2 units of insulin dose before dinner.

Table 11: Example of insulin dose modification for the basal-bolus regimen

Tarikh	Pre-breakfast sugar level (mmol/L)	Insulin dose pre-breakfast (unit)	Pre-lunch sugar level (mmol/L)	Insulin dose pre-lunch (unit)	Pre-dinner sugar level (mmol/L)	Insulin dose pre-dinner (unit)	Pre bed sugar level (mmol/L)	Insulin dose pre-bed (unit)
1.6.20								12
2.6.20	7.2							12
3.6.20	7.8							12
4.6.20	8.5							**14**
5.6.20		12	8.7					
6.6.20		12	7.5					
7.6.20		12	7.2					
8.6.20		**14**		12	7.2			
9.6.20				12	7.8			
10.6.20				12	8.5			
11.6.20				**14**		12	8.7	
12.6.20						12	7.5	
13.6.20						12	7.2	
13.6.20						**14**		

+2 unit

+2 unit

+2 unit

+2 unit

If you inject fast-acting prandial insulin such as Novorapid®, Humalog® or Apidra®, you can check the sugar level 2 hours after a meal. If the sugar level exceeds 8.5 mmol/L 2 hours after breakfast for 3 readings, add 2 units of insulin dose before breakfast the next day. Then check for 2 hours post-lunch. If the sugar level exceeds 8.5 mmol/L 2 hours after lunch for 3 readings, add 2 units of insulin dose before lunch. The same goes for dinner.

Table 12: Example of insulin dose modification for rapid-acting prandial insulin

Date	Pre-breakfast insulin dose (unit)	Blood sugar level 2 hours post breakfast (mmol/L)	Pre-lunch insulin dose (unit)	Blood sugar level 2 hours post lunch (mmol/L)	Pre-dinner insulin dose (unit)	Blood sugar level 2 hours post-dinner (mmol/L)
1.6.20	12	8.7				
2.6.20	12	8.9				
3.6.20	12	8.6				
4.6.20	**14**		12	8.7		
5.6.20			12	8.9		
6.6.20			12	8.6		
7.6.20			**14**		12	8.9
8.6.20					12	8.5
9.6.20					12	8.5
10.6.20					**14**	

+2 unit

+2 unit

+2 unit

UNIT 5 Fasting safely despite injecting insulin

Objective: To increase the knowledge on how to fast safely
Activity: To read and understand Unit 5.
Duration: 5 minutes.

During fasting, diabetic patients have a high risk of getting[9]:

- Hypoglycaemia (low blood sugar),
- Hyperglycaemia (excess sugar in the blood),
- Dehydration (decreased water in the body),
- ketoacidosis (excess acid in the blood), and
- thrombosis (blood clot in a blood vessel).

All of these risks can be reduced with knowledge, monitoring of fasting sugar levels and changes in insulin dosage.

5.1 How to modify insulin dose when fasting?

If you inject basal insulin at bedtime, inject earlier after iftar. You may need to reduce the dose if hypoglycaemia occurs during fasting.

If you use **pre-mixed insulin twice a day**:

- Breakfast dose is used before breaking the fast.
- Dinner dose is used before suhoor. Reduce the dose before suhoor by 20-50%. For example, if you inject 20 units of insulin, reduce the insulin by 4 to 10 units.

If you use **pre-mixed insulin three times a day:**

- The breakfast dose is used before breaking the fast
- Skip the insulin injection for the afternoon.
- Dinner dose is used before suhoor. Reduce the dose before suhoor by 20-50%.

If you inject **basal-bolus** insulin **(4 times)** a day:

- Before suhoor, inject insulin according to the dose before dinner, reduce by 20-50%. Reduce the dose if hypoglycaemia occurs.
- **Skip** the insulin injection for the afternoon.
- Before breaking the fast, inject insulin according to the dose of insulin before breakfast.
- The basal dose of insulin is taken before going to bed or after breaking the fast.

5.2 When do I need to monitor my blood sugar level when fasting?

You are encouraged to monitor your sugar levels at the following times, especially in the early days of fasting. You can choose to monitor 2 or 3 sugar level readings as below:

- Before suhoor and 2 hours after suhoor
- Before breaking the fast and 2 hours after breaking the fast
- When experiencing any symptoms of hypoglycaemia
- If your sugar level has stabilized, you can reduce the frequency of monitoring your sugar level according to your insulin regimen.

5.3 When should I break my fast?

- Sugar level < 3.3 mmol/L during fasting
- Sugar level <3.9 mmol/L in the first few hours of fasting
- Sugar level > 16.7 mmol/L
- Experiencing symptoms of hypoglycaemia (even in the absence of blood sugar measurement)
- Symptoms of severe dehydration such as confusion or coma

References

1. Ministry of Health Malaysia. *Clinical Practice Guidelines Management of Type 2 Diabetes Mellitus*.; 2015. doi:10.1088/1751-8113/44/8/085201
2. Blackburn DF, Swidrovich J, Lemstra M. Non-adherence in type 2 diabetes: Practical considerations for interpreting the literature. *Patient Prefer Adherence*. 2013; 7:183-189. doi:10.2147/PPA.S30613
3. Zanariah H, Nurain MN, Md M. Practical Guide to Insulin Therapy in Type 2 Diabetes. *Minist Heal Malaysia*. 2011:22-30. https://www.researchgate.net/publication/278406704_Practical_Guide_To_Insulin_Therapy_in_Type_2_Diabetes.
4. diabetes.co.uk. Diabetes and Hypoglycemia. https://www.diabetes.co.uk/Diabetes-and-Hypoglycaemia.html. Published 2019. Accessed May 19, 2020.
5. Blood Sugar and Exercise. https://www.diabetes.org/fitness/get-and-stay-fit/getting-started-safely/blood-glucose-and-exercise. Accessed May 19, 2020.
6. Keen H, Pickup JC, Bilous RW, et al. Human Insulin Produced by Recombinant DNA Technology: Safety and Hypoglycæmic Potency in Healthy Men. *Lancet*. 1980;316(8191):398-401. doi:10.1016/S0140-6736(80)90443-2
7. Fonseca VA. Defining and characterizing the progression of type 2 diabetes. *Diabetes Care*. 2009;32 Suppl 2(suppl_2): S151-6. doi:10.2337/dc09-S301
8. McAndrew L, Schneider SH, Burns E, Leventhal H. Does patient blood glucose monitoring improve diabetes control? A systematic review of the literature. *Diabetes Educ*. 2007;33(6):991-1011. doi:10.1177/0145721707309807
9. Hussein Z, Hallaj Rahmatullah I, Mohamad M, Aziz NA, Yusoff Azmi Merican NS. Practical Guide to Diabetes Management in Ramadan. 2015:10.

APPENDIX A: Types of insulin pens available in the market.

Types of Insulin Pens		Insulin Names
KwikPen®	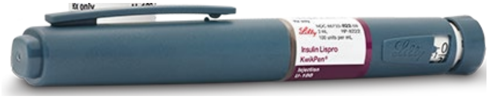	Lispro®
Humalog **HumaPen® (Ergo)**	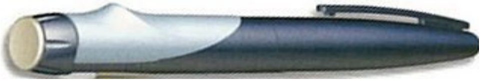	Humalog mix 25<br>Humulin R®<br>Humulin N®<br>Humulin® 30/70<br>Humulin ® 50/50
**Novopen® 3**<br>**Novopen® 4**	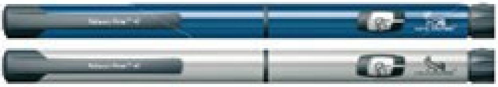	Actrapid®<br>Insulatard®<br>Mixtard® 30/70<br>Novorapid®
**Novolet®**	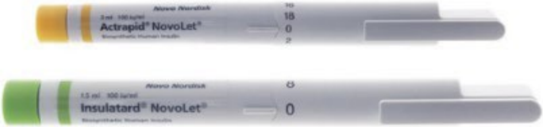	Actrapid®<br>Insulatard®<br>Mixtard® 30/70
**Flexpen®**	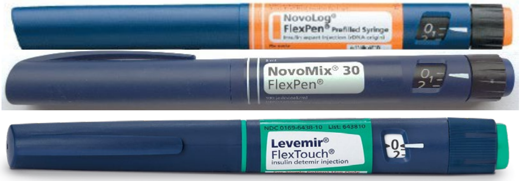	Novorapid®<br>Novomix® 30<br>Levemir®
**SoloSTAR®**	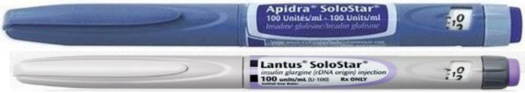	Apidra®<br>Lantus®
	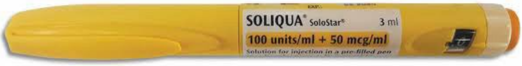	Soliqua®<br>Glargine+<br>lixisenatide

APPENDIX B: Carbohydrate exchange

Limit eating 11-12 servings of carbohydrates in a day, namely:

- 2 fruit exchanges
- 1 sugar exchange
- 1 milk exchange, and
- 7 grain exchanges:

Every picture showed 1 carbohydrate exchange.

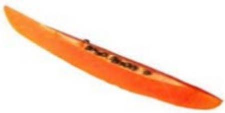

1 slice of papaya/ pineapple/

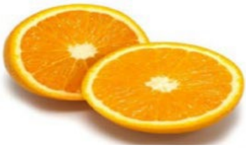

1 orange/pear/kiwi

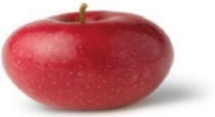

1 apple

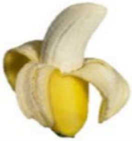

1 small banana

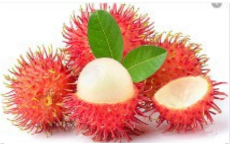

5 rambutans

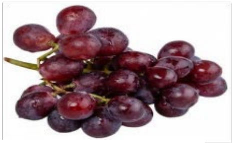

8 grapes

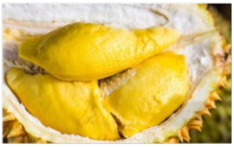

3 cloves of durian

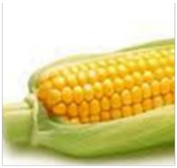

½ cob of corn

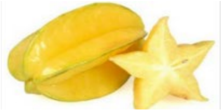

1 starfruit

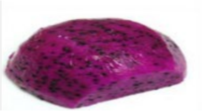

½ dragon fruit

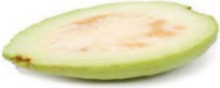

½ guava

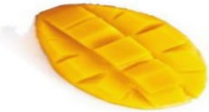

½ mango

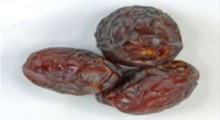

3 dates/ prunes

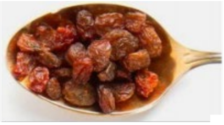

1 tablespoon of raisin

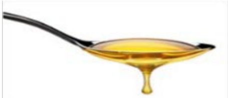

1 tablespoon of honey

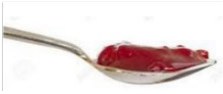

1 tablespoon of jam

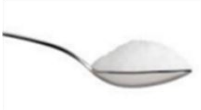

1 teaspoon of sugar

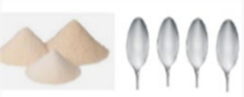

4 tablespoons of milk

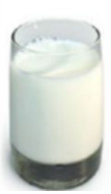

1 glass of milk

Limit grains to 7-8 servings per day

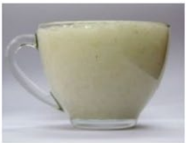
1 cup of congee

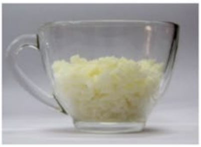
1/2 cup of rice

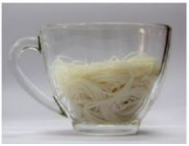
1/2 cup of vermicelli

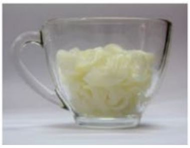
1/2 cup of kuew tiow

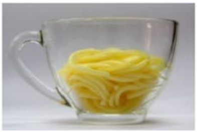
1/2 cup of mee

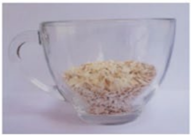
1/2 cup of oat

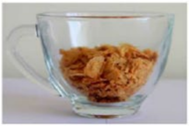
1/2 cup of cereal

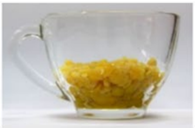
1/2 cup of pasta

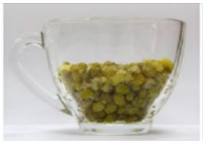
1/2 cup of green beans

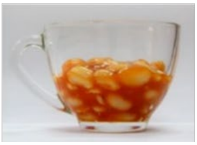
1/2 cup of baked beans

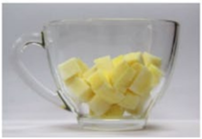
1/2 cup of tapioca

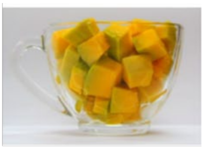
1/2 cup of pumpkin

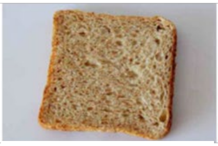
1 slice of bread

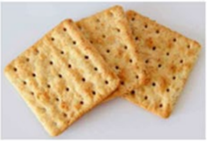
3 pieces of crackers

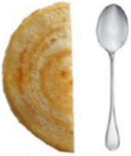
1/2 slice of thosai

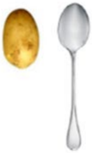
1 small potato

APPENDIX C: Examples of moderate aerobic exercise

You are encouraged to do moderate aerobic exercise for 150 minutes a week.

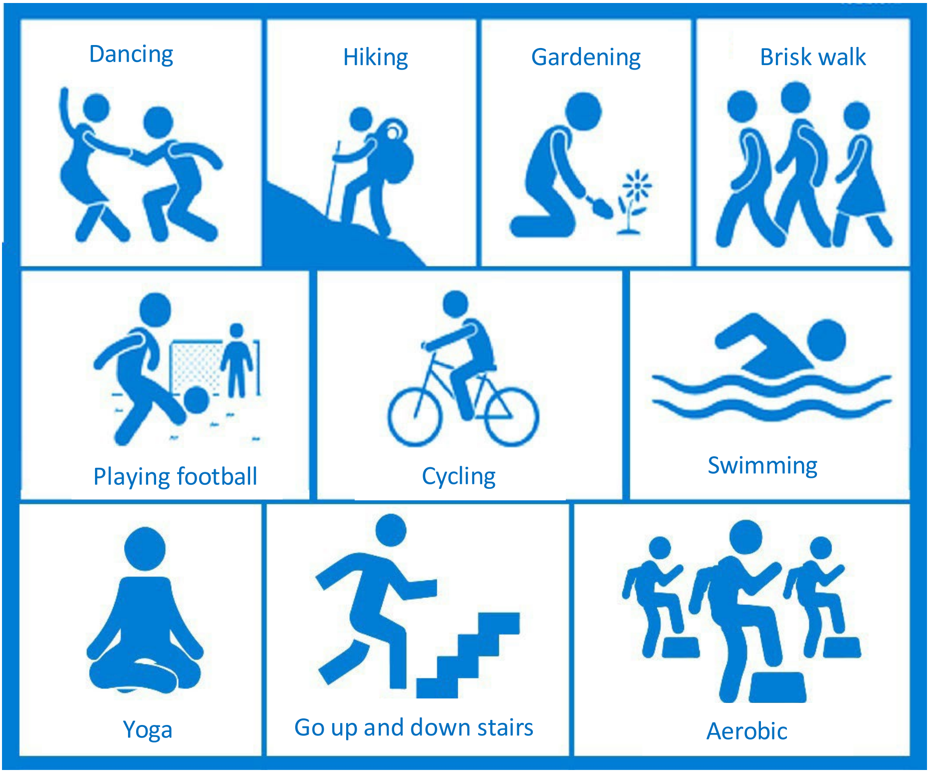

APPENDIX D: Areas of the body suitable for injecting insulin

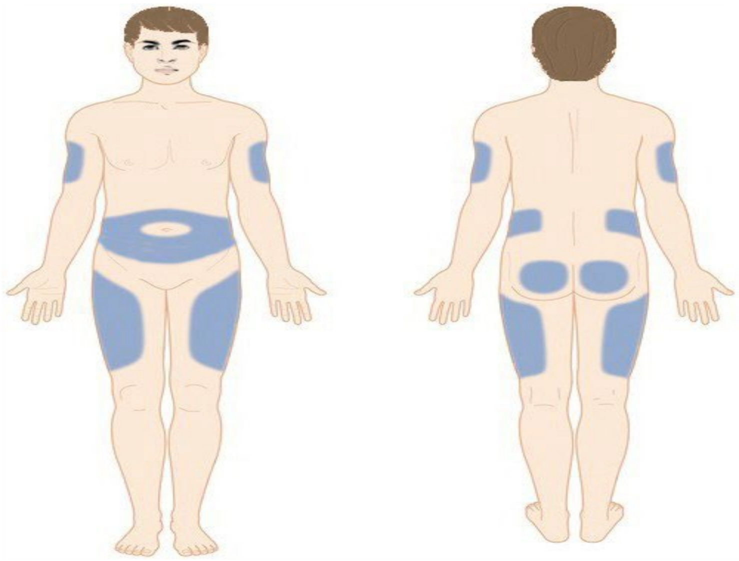

Areas of the body suitable for injection

Inject insulin into the shoulders, thighs, abdomen and buttocks in the entire area alternately to prevent scarring. For example, in the stomach area is like in the picture:

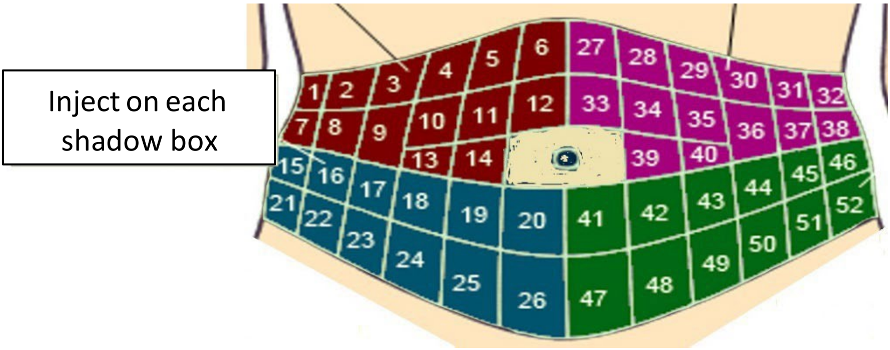

APPENDIX E: Insulin Injection Technique

A: Preparation before injecting insulin

1. Ensure insulin is in good condition.

Check the expiration date of the insulin. Make sure the insulin has not expired.

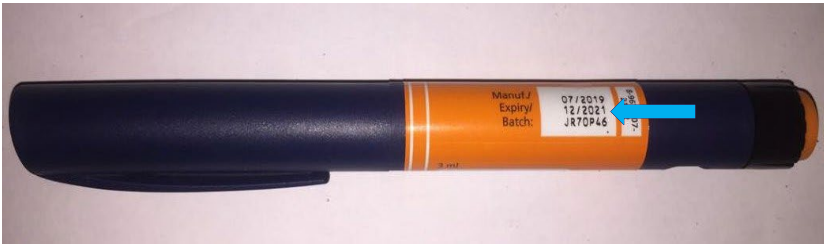

Check the colour of the insulin- clear (prandial) or cloudy (basal/pre-mix). If its colour changes, that means the insulin has been damaged.

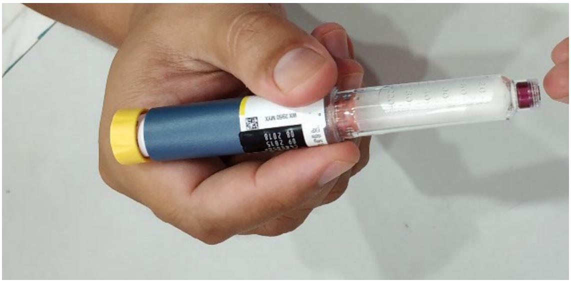

If there is a precipitate, move the insulin pen up and down as in the picture 10 times or roll the insulin pen between two hands for 10 seconds (for newly opened pens)

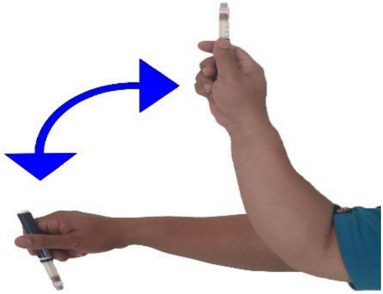

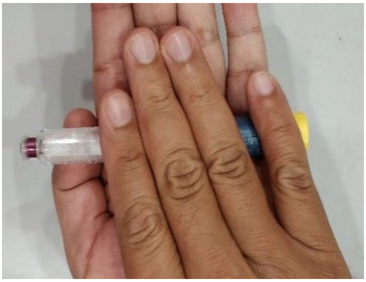

2. Choose a suitable needle
   4/5 mm needle is suitable for all.

3. Install the needle on the insulin pen
   a. Remove the needle wrapping paper.
   b. Attach the needle to the pen by turning the needle clockwise.
   c. Open the outer cover of the needle (transparent).
   d. Open the cap in the needle.

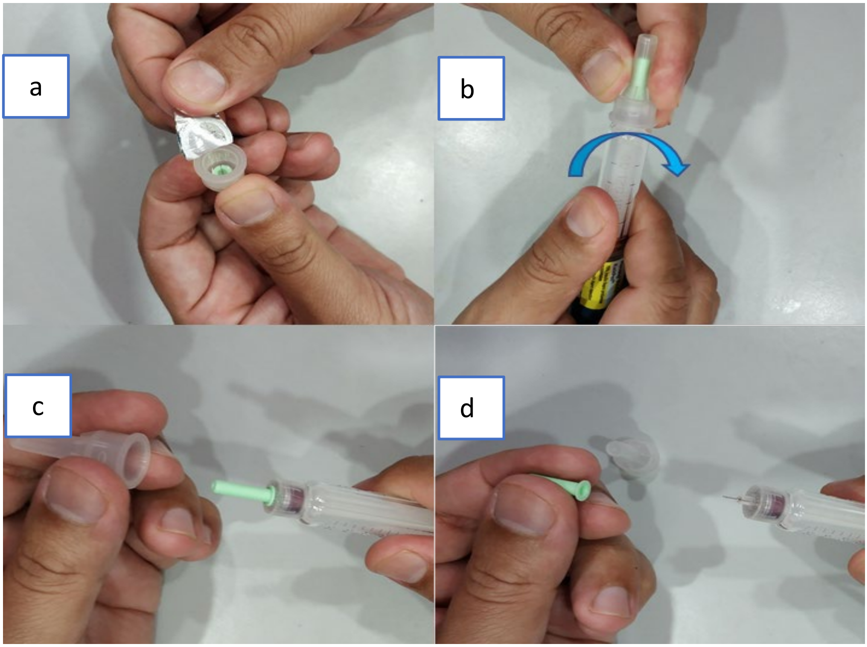

4. Prime the pen.

Turn the dose knob to 1 or 2 unit s

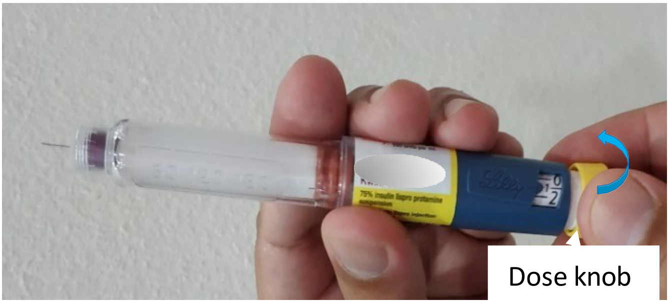

Hold the pen vertically. Press the dose button upwards using your thumb until the dose button shows the number 0.

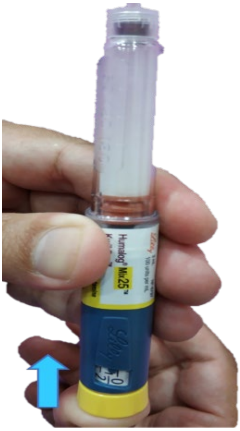

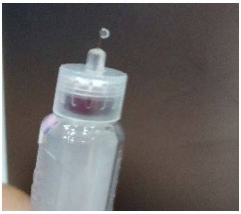

Watch the insulin drip out. (Repeat steps a-c until you can see a drop of insulin coming out)

5. Determine the correct dose of insulin

Turn the dose knob to the desired injection unit. Example in the picture: 12 units

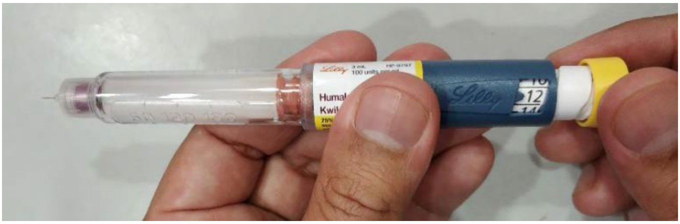

6. Selection and preparation of the injection site

a) Choose the location of the body to be injected as in APPENDIX C. Avoid injecting in the same or scarred place.

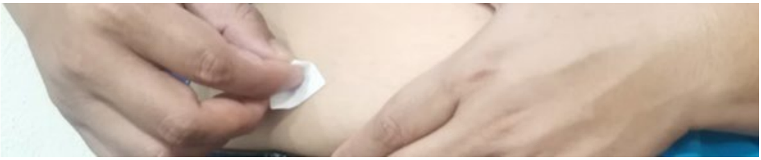

b) While sitting, clean the place to be injected several times with an alcohol swab.

B: Steps of insulin injection

a. Hold the pen with four fingers. Place your thumb on the dose knob.
b. Pinch the part of the skin to be injected.
c. Insert the needle at a 90° angle and release the pinch.
d. Using your thumb, press the dose button until all the insulin goes under the skin (the dose button returns to 0). Wait 10 seconds to prevent insulin from coming out of the injection site. Then, remove the needle.

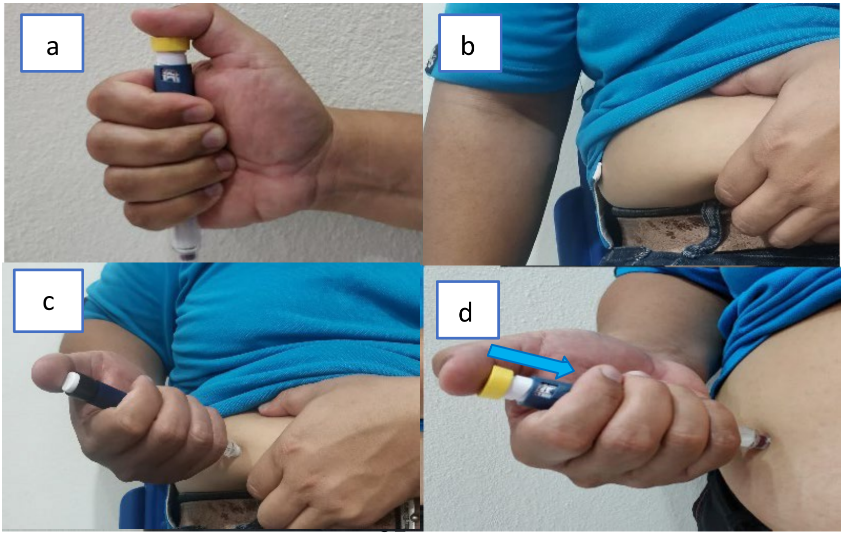

C: Store insulin needles and pens after an injection.

a. Cover the needle with the outer cover of the needle (transparent).
b. Turn the needle counterclockwise until the needle is released from the pen.
c. Cover the pen with the pen cap.
d. Store in a suitable container.

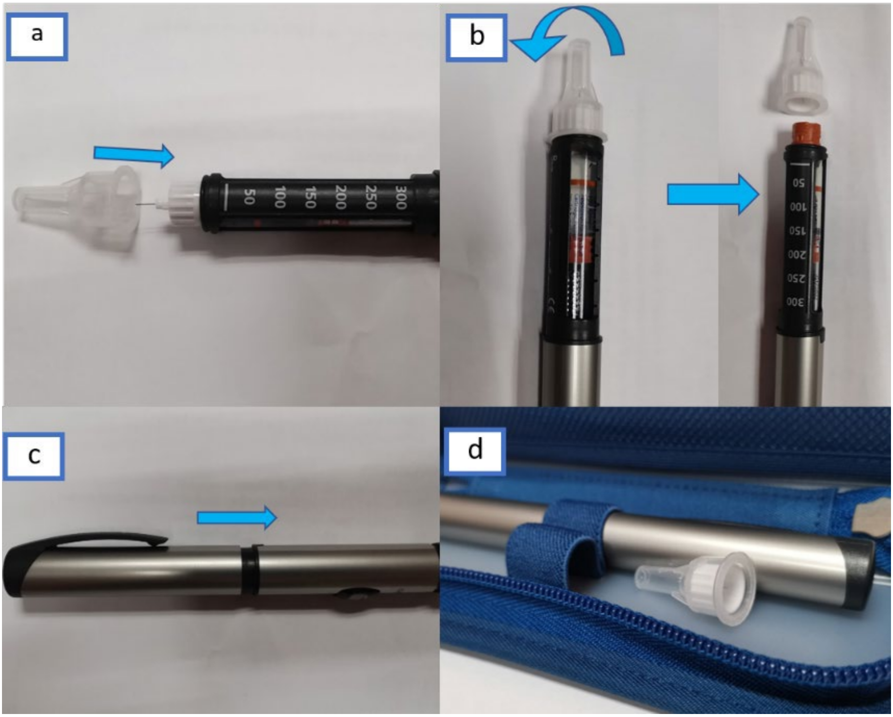

D: How to dispose of insulin needles that have been used three times

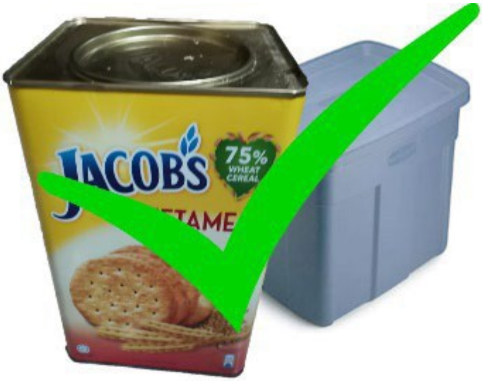

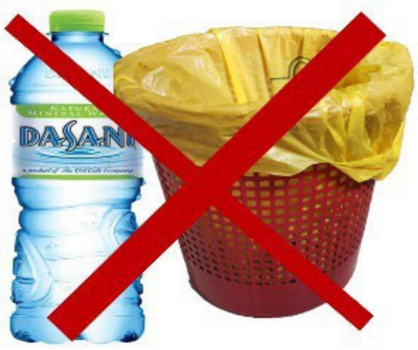

Do not recap the needle with the needle cap. Dispose of the needle in a special closed and safe container.

When the container is full, plant the container.

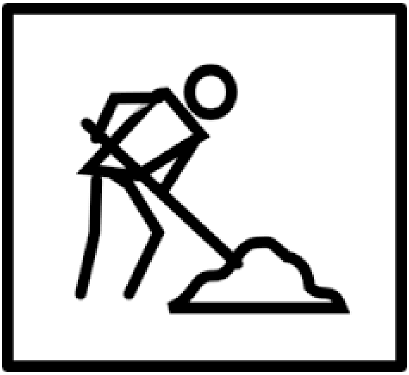

If you don't have a suitable area to plant the container, talk to your nurse/doctor.

APPENDIX F: Modification of insulin dosage during sick day

INSULIN MODIFICATION DURING SICK DAY

When you have a fever or pain, your body will release stress hormones; adrenaline and cortisol. Even if you don't eat, this hormone will cause your blood sugar levels to increase.

You are encouraged to check your sugar level four times a day and modify your insulin dose based on the checked sugar level.

Blood sugar level	Insulin dose modification
< 4 mmol/L	Reduce 4 units from current dose
11.1 - 17 mmol/L	Increase 2 units from current dose
17 - 22 mmol/L	Increase 4 units from current dose
> 22 mmol/L	Increase 6 units from current dose

Get **IMMEDIATE** treatment at the hospital if you have:

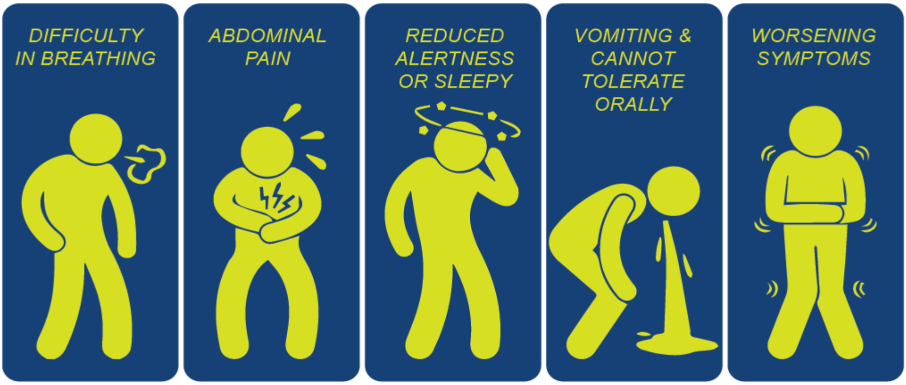

Source: Type 2 diabetes: What to Do When You Are Ill (Diabetes UK,2014)

APPENDIX G: Steps to check your blood sugar level

1. Wash hands with soap and dry.
2. Prepare a poking tool:
   a. Prepare unused needles and lancets

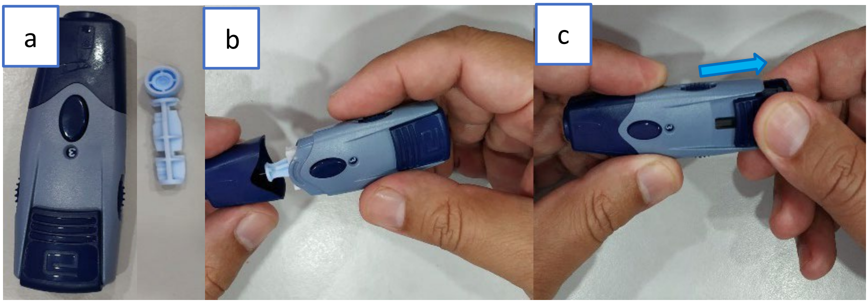

   b. Open the lid of the device. Insert the new needle into place. Open the needle cap.
   c. Close the piercing tool and pull.

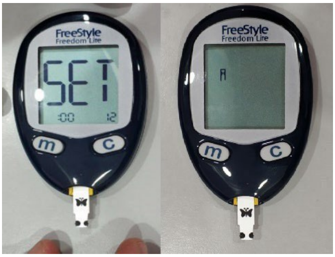

3. Take a strip from the bottle and insert the strip into the glucometer. "Glucometer" will light up. Wait until the blood drop Label appears. This indicates the meter is ready*.

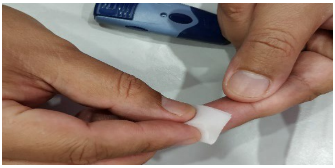

4. Use alcohol cotton to clean the finger that will be injected.

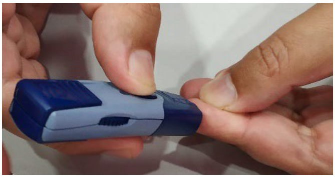

5. Inject the side of the fingertip.

** Follow the instructions as in the respective "glucometer" manual. It may vary between different brands.*

6. Squeeze the finger to remove some blood.

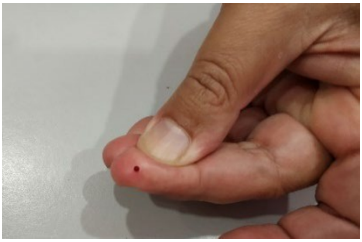

7. Place a drop of blood on the end of the strip until you hear a "beep" sound, or the screen displays a waiting signal. Press the tip of the finger with cotton to stop the bleeding.

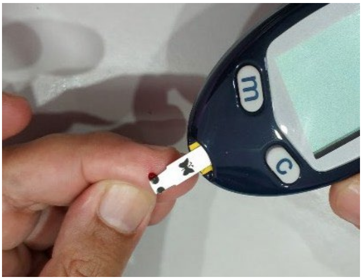

8. Read the sugar level on the glucometer and record it in the diary.

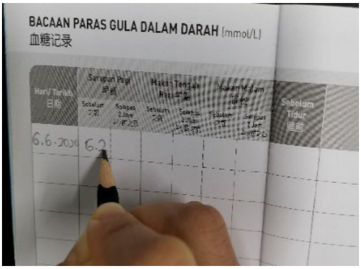

9. Carefully, hold the needle by the side and pull it out of the lancing device. Dispose of in the sharps bin.

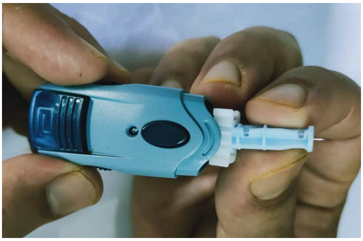

2
