# Supplementary material for: The effect of USM-IAM-based counselling vs standard counselling on insulin adherence, FBS and HbA1c among patients with uncontrolled type 2 diabetes mellitus (T2DM): a randomised controlled trial
Source: BMC Endocr Disord. 2024 Jul 18;24:118. doi: 10.1186/s12902-024-01577-6 (PMC11256455; doi:10.1186/s12902-024-01577-6)
Supplement: Supplementary file 2 — Supplementary Material 2. [file 12902_2024_1577_MOESM2_ESM.pdf]

HOSPITAL UNIVERSITI SAINS MALAYSIA DIABETES CENTRE

BASALOG INSULIN INJECTION AT NIGHT BEFORE SLEEPING

Before bed, inject insulin: _______________unit

(An example of the earliest injection time is 10pm and the latest is 12pm)

SELF-CHECKING OF BLOOD SUGAR LEVEL (SMBG) AND ADJUSTMENT OF INSULIN DOSAGE

Before breakfast, check the blood sugar level. If the blood sugar reading:

Less than 4.0 mmol/L **(hipoglycaemia)** ⟶ reduce 2 unit insulin pre-bed

Between 4.0 and 6.0 mmol/L **(TARGET)** ⟶ maintain insulin dose

more than 6.0 mmol/L **(terlebih dari sasaran)** ⟶ increase 2 unit insulin pre-bed

ATTENTION

*Check the sugar level in the morning every 3 days.
* Change the insulin units either less/more by 2 units every three days
until reaching the sugar target that has been set.
*Target sugar level if there are no signs of low sugar (hypo) 4.0 - 6.0 mmol/L
*Target sugar level if there are signs of low sugar 6.0 - 8.0 mmol/L
*Injection time should be the same every day
*There is no need to eat after the injection UNLESS there are signs of low sugar.

*Please mark the type of insulin directed for injection patients.

HOSPITAL UNIVERSITI SAINS MALAYSIA DIABETES CENTRE

INSUGEN R INSULIN INJECTION 3 TIMES A DAY

MORNING: Inject Insugen R ____________ unit ½ hour pre-breakfast
(Example: Inject at 7.00a.m., take meal at 7.30a.m).

MIDDAY: Inject Insugen R ____________ unit ½ hour pre-lunch
(Example: Inject at 12.30.p.m., take meal at 1.00p.m).

EVENING: Inject Insugen R ____________ unit ½ hour pre-breakfast
(Example: Inject at 6.30p.m., take meal at 7.00p.m).

SELF-CHECKING OF BLOOD SUGAR LEVEL (SMBG) AND ADJUSTMENT OF INSULIN DOSAGE

Before midday dose, check your sugar level. If blood sugar level:

Less than 4.0 mmol/L **(hypoglycemia)** ⟶ Reduce 2 unit pre-breakfast dose

Between 4.0 and 6.0 mmol/L **(TARGETS)** ⟶ Maintain pre-breakfast dose

More thab 6.0 mmol/L **(EXCEEDS TARGET)** ⟶ INCREASE 2 unit pre-breakfast dose

ATTENTION

*Check the sugar level at noon every 3 days.
* Change the insulin units either less/more by 2 units every three days until reaching the sugar target that has been set.
*Target sugar level if there are no signs of low sugar (hypo) 4.0 - 6.0 mmol/L.
*Target sugar level if there are signs of low sugar 6.0 - 8.0 mmol/L.
*Injection time should be the same every day.
*If you do not eat/drink sweets DO NOT INJECT.
*Avoid waiting for injection and mealtime.

PUSAT DIABETES HOSPITAL UNIVERSITI SAINS MALAYSIA

NOVORAPID INSULIN INJECTION 3 TIMES A DAY

MORNING: Inject Novorapid ____________ unit immediately before breakfast (Example: Inject before 8a.b).

MIDDAY: Inject Novorapid ____________ unit immediately before lunch (Example: Inject before 1.00p.m).

EVENING: Inject Novorapid ____________ unit immediately before dinner (Example: Inject before 7.00p.m).

SELF-CHECKING OF BLOOD SUGAR LEVEL (SMBG) AND ADJUSTMENT OF INSULIN DOSAGE

Before midday dose, check your sugar level. If blood sugar level:

Less than 4.0 mmol/L ⟶ Reduce 2 unit pre-breakfast dose
(hypoglycemia)

Between 4.0 and 6.0 mmol/L ⟶ Maintain pre-breakfast dose
(TARGETS)

More thab 6.0 mmol/L ⟶ INCREASE 2 unit pre-breakfast dose
(EXCEEDS TARGET)

ATTENTION

*Check the sugar level at noon every 3 days.
* Change the insulin units either less/more by 2 units every three days until reaching the sugar target that has been set.
*Target sugar level if there are no signs of low sugar (hypo) 4.0 - 6.0 mmol/L.
*Target sugar level if there are signs of low sugar 6.0 - 8.0 mmol/L.
*Injection time should be the same every day.
*If you do not eat/drink sweets DO NOT INJECT.
*Immediately consume meal after injection.

PUSAT DIABETES HOSPITAL UNIVERSITI SAINS MALAYSIA

NOVOMIX INJECTION 2 TIMES A DAY

MORNING Inject immediately before breakfast: Novomix ____________ unit (example breakfast before 8 am).

EVENING Inject immediately before dinner: Novomix____________unit (example dinner before 7pm).

SELF-CHECKING OF BLOOD SUGAR LEVEL (SMBG) AND ADJUSTMENT OF INSULIN DOSAGE

Before injecting pre-breakfast, check your sugar level. If blood sugar level:

Less than 4.0 mmol/L **(hypoglycemia)** → Reduce 2 unit pre-dinner dose

Between 4.0 and 6.0 mmol/L **(TARGETS)** → Maintain pre-dinner dose

More thab 6.0 mmol/L **(EXCEEDS TARGET)** → INCREASE 2 unit pre-dinner dose

ATTENTION

*Check the sugar level at noon every 3 days.
* Change the insulin units either less/more by 2 units every three days until reaching the sugar target that has been set.
*Target sugar level if there are no signs of low sugar (hypo) 4.0 - 6.0 mmol/L.
*Target sugar level if there are signs of low sugar 6.0 - 8.0 mmol/L.
*Injection time should be the same every day.
*If you do not eat/drink sweets DO NOT INJECT.
