# Supplementary material for: The effect of USM-IAM-based counselling vs standard counselling on insulin adherence, FBS and HbA1c among patients with uncontrolled type 2 diabetes mellitus (T2DM): a randomised controlled trial
Source: BMC Endocr Disord. 2024 Jul 18;24:118. doi: 10.1186/s12902-024-01577-6 (PMC11256455; doi:10.1186/s12902-024-01577-6)
Supplement: Supplementary file 3 — Supplementary Material 3. [file 12902_2024_1577_MOESM3_ESM.pdf]

DIABETES
Education Manual 2016

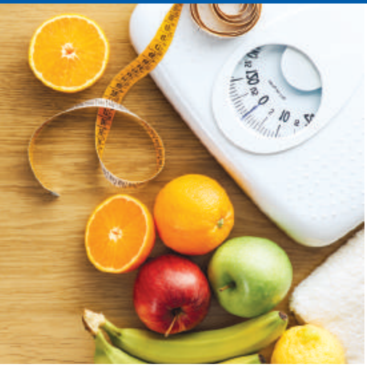
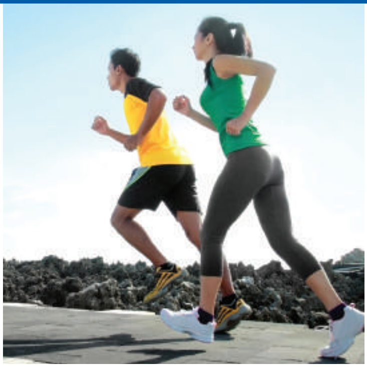
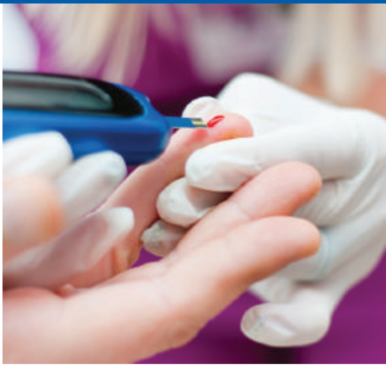
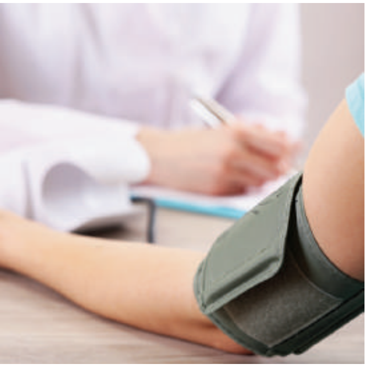
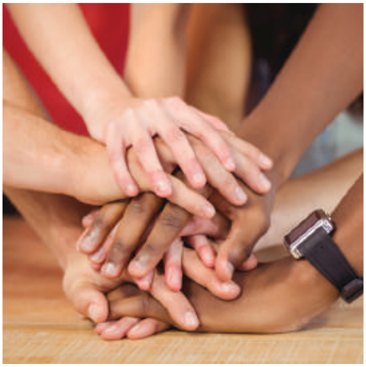
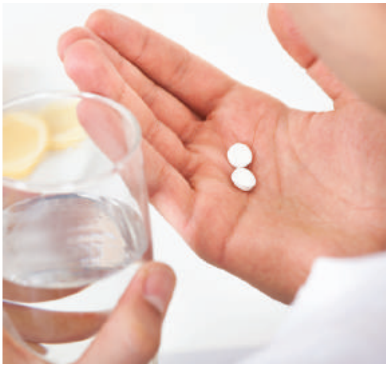
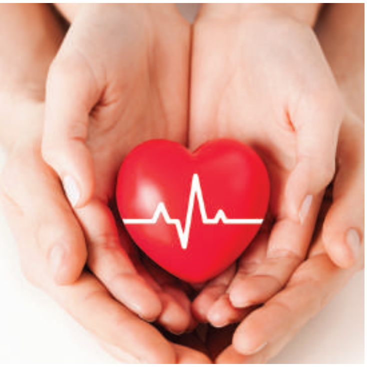
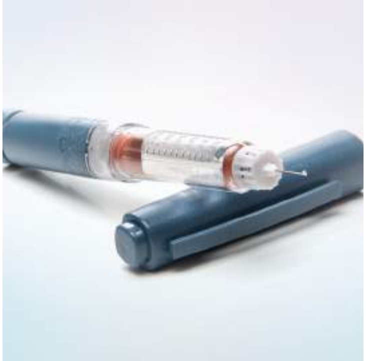
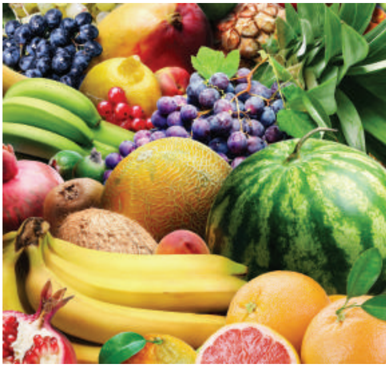

Ministry of Health Malaysia

Malaysian Diabetes Educators Society

First published April 2016

By The Malaysian Diabetes Educators Society

TABLE OF CONTENTS

TABLE OF CONTENTS		PAGE NO
FOREWORD		4
PREFACE		5
MANUAL OBJECTIVES		6
MANUAL WORKING COMMITTEE		6
EXTERNAL REVIEWERS		8
SECTION 1	INTRODUCTION TO DIABETES SELF-MANAGEMENT EDUCATION	9
SECTION 2	EDUCATION ASSESSMENT	11
SECTION 3	HEALTHY EATING	22
SECTION 4	PHYSICAL ACTIVITY AND EXERCISE	31
SECTION 5	MEDICATION	42
SECTION 6	SELF-MONITORING	72
SECTION 7	RISK REDUCTION	94
SECTION 8	BEHAVIOURAL INTERVENTION	124
GLOSSARY OF TERMS		135
ACKNOWLEDGEMENTS		137
SOURCE OF FUNDING		137

FOREWORD

The Ministry of Health congratulates the Malaysian Diabetes Educators Society for developing the Malaysian Diabetes Education Manual, 1st edition, 2016.

Diabetes is a globally growing disease and managing the disease is highly complex for it requires attention from many parties. It has been recognized that to control the disease effectively, it relies on three main factors; the competency of the healthcare professionals, commitment of people living with diabetes and the availability of resources. Experts of diabetes care suggested a multidisciplinary integrated care to impede the disease from growing in the primary, secondary and tertiary care areas. Research for new drugs and advanced technologies for the invention of sophisticated diabetes management instruments have been ongoing since the discovery of the disease. Living in the modern world, new drugs and advanced technologies are insufficient to manage the disease without the person living with diabetes being committed and taking ownership in practicing healthy living daily. The Malaysian Diabetes Education Manual is a timely piece of production. It provides valuable guidelines for healthcare professionals making useful approaches in couching people with diabetes.

This manual is recommended for healthcare professionals who are dealing with people with diabetes or at risk for diabetes. It is a comprehensive manual that adopts the American Association of Diabetes Educator AADE7™ Self Care Behaviour System in formulating the guidelines. The guidelines have been written systematically based on healthy eating, being active, monitoring, taking medication, problem solving, reducing risk and healthy coping. Reducing the prevalence of diabetes is one of the priority healthcare targets of the Ministry of Health. Diabetes self-care knowledge among the people living with diabetes in Malaysia is generally requires improvement. The Malaysian Diabetes Education Manual in one way or another shall serve it purpose in promoting a better standard of diabetes care in the country.

The Ministry of Health commends the Malaysian Diabetes Educators Society for taking the initiative in making this handy manual and all the healthcare professionals for their efforts in contributing to the manual development.

Datuk Dr Noor Hisham Bin Abdullah
Director General of Health
Ministry of Health Malaysia

PREFACE

The 2015 International Diabetes Federation Congress refers to Diabetes Mellitus as ”one of the fastest epidemics in human history”, due to its rapid rise in prevalence worldwide. Malaysia is not immune to this epidemic. The prevalence of diabetes in Malaysia in the 1960’s was 6.2%, by 2011 it has increased to 20%. In developed countries like the USA and Canada, 50% of people with diabetes manage to achieve targeted glycaemic control. However less than 25% of Malaysians with diabetes manage to achieve similar targets. It is no surprise that this results in a high rate of diabetes complications locally.

Even with the availability of more novel pharmacological anti-diabetes agents, patient empowerment to achieve behavioural change via structured Diabetes Self-management Education and Support, continues to play an important role in improving clinical outcomes. With the high prevalence of diabetes in Malaysia, the number of trained diabetes educators is insufficient to provide the knowledge and skills required for self-care. To narrow the gap of facilitating diabetes education in clinical practice, other healthcare professionals can play an active role using this manual as a guide.

Several Clinical Practice Guidelines related to management of Type 2 diabetes have been published in Malaysia such as for Management of Type 2 diabetes, Practical Guidelines for Type 2 Insulin Treatment, Medical Nutrition Therapy Guideline for Type 2 Diabetes and Screening for Diabetes Retinopathy. However until today there is no clinical practice guideline or related guideline on Diabetes Education. This is the first Diabetes Education Manual developed in Malaysia. The aim of this manual is to standardise the content and process for structured diabetes education to ensure the education and support received by individuals with diabetes and those at risk of diabetes are of high quality which can significantly contribute to improved metabolic and psychological outcomes.

To address the gap between diabetes management and education, I hope this manual will be fully utilized by all relevant health care professionals. Last but not least I would like to express my sincere gratitude to everyone involved in the development of this manual and especially to the task force members and reviewers for their immense support and contribution.

Tan Ming Yeong RN CDE
Chair, Diabetes Education Manual Committee

Manual Objectives

The aim of the diabetes education manual is to serve as a guide to standardise the structure and content of diabetes education while taking into the account the importance of individual needs.

Target Population

This education manual provides the educational process and content for adults 18 years old and above with Type 2 Diabetes Mellitus.

Who Should Use This Manual

This diabetes education manual may be used by healthcare professionals who provide diabetes education for people with Type 2 Diabetes including diabetes educators, nurses, assistant medical officers, dietitians, pharmacists, physiotherapists, clinical psychologists and medical practitioners.

Manual Working Committee

CHAIRPERSON

Dr. Tan Ming Yeong
Diabetes Nurse Specialist, International Medical University, Kuala Lumpur

**Adeline Tay Guek Peng**<br>Diabetes Educator<br>AstraZeneca Sdn Bhd<br>Kuala Lumpur	**Dr. Arlene Ngan**<br>Consultant Endocrinologist<br>Kuala Lumpur
**Chew Wai Fong**<br>Lecturer (Nutrition)<br>University Tunku Abdul Rahman<br>Sungai Long, Selangor	**Daljeet Kaur**<br>Diabetes Nurse Educator<br>Clinic Daljeet Sandhu<br>Puchong, Selangor
**Foong Pui Hing**<br>Principal Dietitian<br>National Heart Institute<br>Kuala Lumpur	**Grace Goh Siew Ying**<br>Pharmacist<br>Pharmacy Service Division<br>The Federal Territories of Kuala Lumpur and Putrajaya
**Dr. Jeshen Lau Hui Giek**<br>Consultant Endocrinologist<br>University Malaysia Sarawak<br>Sarawak	**Lee Lai Fun**<br>Adjunct Senior Lecturer (Dietetics)<br>International Medical University<br>Kuala Lumpur

**Lim Chooi Imm**<br>Lecturer (Nursing)<br>Institute of Science and Technology<br>Darul Takzim, Johor	**Mary Easaw**<br>Senior Manager Dietetics & Food Services<br>National Heart Institute<br>Kuala Lumpur
**Dr Mastura Ismail**<br>Consultant Family Medicine<br>Seremban 2 Health Clinic<br>Seremban, Negri Sembilan	**Dr. Mohd Nahar Azmi Mohamed**<br>Head of Sports Medicine Department<br>University Malaya Medical Centre<br>Kuala Lumpur
**Pee Lay Ting**<br>Pharmacist<br>Hospital Kuala Lumpur<br>Kuala Lumpur	**Poh Kai Ling**<br>Clinical Dietitian<br>University Malaya Medical Centre<br>Kuala Lumpur
**Dr. Sasikala Devi Amirthalingam**<br>Family Medicine Specialist<br>International Medical University<br>Kuala Lumpur	**Siah Guan Jian**<br>Senior Diabetes Educator<br>National Heart Institute<br>Kuala Lumpur
**Tan Pei Jun**<br>Clinical Psychologist<br>Ting Psychiatrist Clinic<br>Puchong, Selangor	**Tan Sing Ean**<br>Clinical Dietitian<br>Hospital Kuala Lumpur<br>Kuala Lumpur
**Vasanthi Ganesan**<br>Diabetes Nurse Educator<br>KPJ Kajang Specialist Hospital<br>Selangor	**Violet Choo Khee Ling**<br>Nurse Manager Diabetes Care Centre<br>Gleneagles Hospital<br>Kuala Lumpur
**Wong Yoke Lian**<br>Senior Diabetes Educator<br>LifeScan, Johnson & Johnson Sdn Bhd<br>Kuala Lumpur	**Woo Li Fong**<br>Nurse Specialist<br>University Tunku Abdul Rahman<br>Sungai Long, Selangor
**Yong Lai Mee**<br>Nurse Manager Diabetes Care Services<br>Subang Jaya Medical Centre<br>Subang Jaya, Selangor	

External Reviewers

Prof. Dr. Winnie Chee Siew Swee
Consultant Dietitian
International Medical University
Kuala Lumpur
Malaysia

Prof. Dr. Trisha Dunning
Inaugural Chair in Nursing
Deakin University and Barwon Health
Melbourne
Australia

Dr. Zanariah Hussein
Senior Consultant Endocrinologist
Putrajaya Hospital
Putrajaya
Malaysia

Dr. Hjh Rotina Abu Bakar
Senior Principal Assistant Director (NCD)
Disease Control Division
Ministry of Health Malaysia
Malaysia

Puan Noraini Mohamad
Senior Pharmacist
Division of Pharmaceutical Services
Ministry of Health Malaysia
Putrajaya
Malaysia

Section 1

INTRODUCTION TO DIABETES SELF-MANAGEMENT EDUCATION

Diabetes Mellitus is a lifelong disease. Daily management is primarily self-directed as people with diabetes spend less than 5% of their life with healthcare professionals who care for them. It is essential that they are able to self-manage their condition to achieve optimal health, good quality of life, minimize diabetes related complications and reduce the need for costly healthcare.

One approach to empower people with diabetes is by creating access, training and support for diabetes self-management education (DSME). DSME is defined as "collaborative process through which people with or at risk for diabetes gain the knowledge and skills needed to modify behaviour and successfully self-manage the disease and its related conditions" (Burke et al, 2014). DSME has been shown to be associated with improved diabetes outcomes, reduced diabetes complications and decrease in healthcare costs (American Diabetes Association, 2014).

It is still a common impression that diabetes education means transfer of information / knowledge from healthcare professionals to people with diabetes using the compliance / adherence model. Over the past decades, it became clear that while knowledge is an essential prerequisite for self-care, ***knowledge alone is not enough to promote behaviour change*** (Brown, 1999; Norris et al, 2002). In response to a growing body of evidence, there has been a paradigm shift from the didactic (lecture) teaching style of self-management skills to a patient-centred facilitation approach that encourages empowerment and self-efficacy to promote behaviour change (Anderson and Funnell, 2005). Behaviour change is the unique outcome criterion for effective diabetes education. It is critical that the person with diabetes (and caregivers) has the knowledge, skills and behaviours needed to successfully manage the disease (Hass et al, 2014).

The American Association of Diabetes Educators recommends that successful and effective diabetes management involves seven specific self-care behaviours. They are Healthy Eating, Being Active, Monitoring, Taking Medication, Reducing Risk, Problem Solving and Healthy Coping (Peeples et al, 2007). In this manual, the discussion starts with Education Assessment followed by the first five of the seven self-care behaviours. Problem solving and healthy coping are discussed in the behavioural intervention section.

Structured Diabetes self-management Education involves the 5-steps of educational process namely assessment, goal setting, planning, implementation and evaluation. This should be individualized and in collaboration with the person with diabetes (and caregivers). The subsequent sections in this manual follow the 5-steps of DSME and the process is detailed in its own section.

References

1. American Diabetes Association (2014) Standard of Medical Care in Diabetes. *Diabetes* Care Vol.37, S14-S79.

2. Anderson,R.M. and Funnell, M.M. (2005) Patient empowerment: reflections on the challenge of fostering and adoption of a new paradigm. *Patient Education Counseling* Vol.57 (2), 53-57.

3. Burke, S.D., Thorlton, J. and Hall, M. (2014). Diabetes Self-Management Education: The Art and Science of Disease Management. In Mensing, C., Cornell, S., and Halstenson, C. (3rd ed.), *The art and science of diabetic self-management education desk reference* (pp.3-30). Chicago: American Association of Diabetes Educators.

4. Brown, S.A. (1999) Interventions to promote diabetes self-management: state of the science. *Diabetes Educator* Vol.25, S52-S61.

5. Hass, L., Maryniuk, M., Beck, J. et al (2014) National Standard for Diabetes Self-management Education. *Diabetes Care* Vol.37, S155-S163.

6. Norris, S.L., Lau, J., Smith, S.J. et al (2002) Self-management education for adults with Type 2 Diabetes: a meta-analysis on the effect on glycaemic control. *Diabetes Care Vol.5, 1159-1171.*

7. Peeples, M., Tomky, D., Mulcahy, K. et al (2007) Evolution of the American Association of Diabetes Educators' diabetes education outcomes project. *The Diabetes Educators* Vol.33 (5), 794-817.

Section 2

EDUCATION ASSESSMENT

No		Content	Page
2.1		Initial Assessment	12
	2.1.1	Introduction	12
	2.1.2	Aim	12
	2.1.3	Objectives	12
	2.1.4	Assessment Topics	12
	2.1.5	Goal Setting	15
	2.1.6	Planning	15
	2.1.7	Implementation	15
2.2		Follow-up Assessment	16
	2.2.1	Introduction	16
	2.2.2	Review History	16
	2.2.3	Examination	16
	2.2.4	Implementation	17
	2.2.5	Evaluation	17
2.3		References	17
2.4		Appendices	18

2.1 INITIAL ASSESSMENT

2.1.1 Introduction

Assessment is the initial phase of diabetes education. It is a vital process for the diabetes educator to build rapport with the person with diabetes. More importantly it helps to identify the needs of the person with diabetes and sets individualized goals for a diabetes education program. The assessment, education, plan, intervention and outcomes shall be documented in the education record (American Association of Diabetes Educators, 2011).

2.1.2 Aim

The aims of initial assessment are to identify the baseline of an individual's diabetes knowledge, skills and confidence in taking control of their own condition and to develop an individualized educational plan directed towards empowering the individual to make appropriate choices regarding self-managing diabetes.

2.1.3 Objectives

- To determine the cognitive, affective and psychomotor level of an individual or caregiver in learning diabetes self-management.
- To identify the learning needs of an individual or caregiver in self-managing diabetes.
- To identify barriers towards empowering the individual or caregiver in achieving self-management diabetes targets.
- To plan an individualized diabetes education program that could engage the individual and their caregiver in implementing an effective diabetes self-management plan.
- To formulate measurements to evaluate learning, behavioural and clinical outcomes as well as levels of satisfaction and well being.

2.1.4 Assessment Topics

<u>Demographic and anthropometric data</u>

- Name, age, ethnicity, religion
- Marital status (e.g. single, married, divorced, separated, or widowed)
- Living arrangements (e.g. staying with family members, friends, others or alone)
- Preferred language
- Education level (e.g. none, primary, secondary, college or tertiary)
- Occupation
- Weight, height, BMI, waist circumference
- Blood pressure

Physical factors

- Including mobility, visual ability, hearing, manual dexterity, alertness
- Special needs or limitations for example adaptive support or requiring special accommodation like staying at nursing home

Nutrition history and practices (Baron, 2014) – Refer Section 3

- Details of latest dietitian review
- Timing of meals
- Frequency of meals
- Frequency of snacks
- Frequency of supper
- Frequency of sweetened beverages (e.g. glasses / day, days / week)

Physical activity – Refer Section 4

- Physical activity at work (e.g. light, moderate, vigorous)
- Physical activity during leisure time (e.g. light, moderate, vigorous)
- Type of physical activity
- Frequency of physical activity (e.g. minutes / week, times / week) (Warburtor et al, 2010).

Risk factors

- Smoking, cigarettes/day
- Alcohol, type and frequency (e.g. days/ week, quantity / week)

Medication

- Name, dose, frequency
- Timing
- Adherence to medication regimen

Monitoring of blood glucose

- Frequency and schedule of monitoring (e.g. times/ day, days / week)
- Review of logbook

Hypoglycemia

- Frequency of hypoglycemia (e.g. times/ day, days/ week, times/ month, rarely or never)
- Signs, symptoms, causes
- Treatment, require assistance

Foot assessment

- Details of recent foot assessment

Biochemical Tests

- Fasting/random blood sugar
- HbA1c
- Creatinine
- eGFR
- Microalbuminuria
- Total Cholesterol
- HDL Cholesterol
- LDL Cholesterol
- Triglycerides

Basic knowledge and skills in managing diabetes

- Previous diabetes education, actual knowledge and skills
- Assess skills in managing diabetes (e.g. monitoring blood sugar technique, insulin administration technique.)
- Assess diabetes knowledge by asking sample questions (refer table 1)

Table 1: Sample Questions to Assess Diabetes Knowledge

What is the effect of the following on blood glucose?<br>*Please tick (√) the appropriate column on your right.*	Increase	Decrease	No Effect	Not sure
i. Taking oats				
ii. Taking wholemeal bread				
iii. Taking green apple				
iv. Taking fried chicken				
v. Taking nuts				
vi. Drinking fruit juice				
vii. Physical activity such as walking, cycling or swimming				
viii. Taking oral anti-diabetic medication/insulin injection but missing meal				
ix. Missing anti-diabetic medications or insulin injection				
x. Having infection such as cold or flu				

2.1.5 Goal Setting

Involvement of the person with diabetes is critical for achievement of goals. Effective goal setting for each counseling session is both self-directed by the person with diabetes and a collaboration between the diabetes educator and the person with diabetes.

2.1.6 Planning

The diabetes educator develops the plan to attain the mutually defined goals and outcomes with regards to each specific self-care behaviour (healthy eating, physical activity and exercise, medication intake, self-monitoring, risk reduction and behaviour intervention).

2.1.7 Implementation

The diabetes educator provides education according to the agreed upon plan. The educator guides implementation of the diabetes education plan and interfaces with the various care providers, person with diabetes and caregivers. Implementation also may be linked to other professional services and resources. Good communication is fundamental for effective implementation because it is important that the person with diabetes fully understands and is able to perform the tasks defined in the plan (Ishikawa and Kuchi, 2010).

2.2 FOLLOW-UP ASSESSMENT

2.2.1 Introduction

The diabetes self-management education program recommended for persons with Type 2 diabetes is comprised of an initial "introductory" session and subsequent follow-up sessions at one month, three months and six months. After the completion of the program at 6 months, people with diabetes are scheduled for follow-up sessions as needed annually (American Association of Diabetes Educators, 2011).

In the follow-up session, a systematic assessment is done to evaluate the outcomes of the previous education session. Many people with diabetes have work or financial stresses. Some may require lifestyle changes such as alterations in eating habits, sleep pattern, smoking cessation, exercise initiation and pain issues that may affect their priorities.

The follow up assessment process includes:

2.2.2 Review History (Mulcahy et al, 2003)

History	Examples
• Lifestyle factors	- Meal planning, physical activities, smoking and sleeping pattern
• Clinical investigation results	- HbA1c, lipid profile, renal profile
• Medication related problems	- Side effect and adherence
• Self-monitoring blood glucose record	- Frequency, timing
• Foot symptoms and foot care	- Paraesthesia, numbness, types of foot wear
• Challenges with diabetes self-management	- Engaging in social activities and daily life

2.2.3 Examination

- Check weight and waist circumference.
- Check blood pressure.
- Perform feet examination if new symptoms or at risk (e.g. neuropathy ± Peripheral vascular disease).

2.2.4. Implementation

- Determine specific self-care issues for discussion within the education session.
- Identify supplementary education goals with individual involvement.
- Renew diabetes self-management plan with identified interventions.

2.2.5. Evaluation

- Learning outcomes
- Behavioural changes such as adherence to lifestyle changes
- Achievement of clinical targets

2.3 References

1. American Association of Diabetes Educators, AADE (2011) Guidelines for the Practice of Diabetes Self-Management Education and Training. Website: http://www.diabeteseducator.org/export/sites/aade/_resources/pdf/general/PracticeGuidelines2011.pdf. Accessed 16 December 2014.

2. Baron, R.B.(2014) Nutritional Disorders. In Papadakis, M., McPhee, S.J. and Rabow, M.W.(eds). *Current Medical Diagnosis & Treatment 2014*, McGraw-Hill Education, USA.

3. Ishikaw,H.and Kiuchi,T. (2010) Review: Health literacy and health communication. *Bio psychosocial Medicine* Vol.4, 18-22.

4. Mulcahy, K., Maryniuk, M., Peeples, M. et al. (2003) Diabetes Self-Management Education Core Outcomes Measures. *The Diabetes Educator* Vol.29, 768-802.

5. Warburton, D. Er., Charleswort, S., Ivery, A. et al. (2010) A systematic review of the evidence for Canada's Physical Activity Guidelines for Adults International *Journal of Behavioral Nutrition and Physical Activity* Vol.7, 1-22.

2.4 Appendices

2.4.1 Initial Assessment Form

Name ____________________

Date of Birth ____________________

MRN ____________________

(AFFIX PATIENT LABEL HERE)

Inpatient ☐ Outpatient ☐

Date: ____________________

Referred by: ____________________

Reason for Referral: ____________________

DEMOGRAPHIC DATA:

Duration of diabetes: ________ years Preferred Language: ____________________

Education: ☐ Never ☐ Primary ☐ Secondary ☐ Tertiary

Marital Status: ☐ Single ☐ Married ☐ Divorced ☐ Separated ☐ Widowed

Living with: ☐ Family member ☐ Friends ☐ Alone ☐ Others

Residence: ____________________ Occupation: ____________________

Family History: DM ☐ No ☐ Yes Mother ( ) Father ( ) Siblings ( ) Children ( )

Medical History: ____________________

Surgical History: ____________________

CURRENT DIABETES TREATMENT:

Any changes in diabetes medicines for this visit: ☐ No ☐ Yes

Type of changes: ____________________

Oral Anti-diabetic: ____________________

Insulin: ☐ Basal ____________________

☐ Bolus ____________________

☐ Premix ____________________

Others: ____________________

Adherence to medicines (MMAS-4): ☐ High adherence ☐ Medium adherence ☐ Low adherence

DIET:

Last dietitian review: ____________________

Regularity of meals: ☐ Breakfast ☐ Lunch ☐ Dinner

Snacks: ☐ Yes ☐ No ☐ Sometimes

Supper: ☐ Yes ☐ No ☐ Sometimes

Comments: ____________________

PHYSICAL ACTIVITY:

Physical activity at work: ☐ Light ☐ Moderate ☐ Vigorous

Physical activity during leisure time: ☐ Light ☐ Moderate ☐ Vigorous

Type of activity, times per week/hrs. ____________________

RISK FACTORS:

Smoking: No ☐ Yes ☐ ____________ cigarettes/day

Alcohol: No ☐ Yes ☐ ____________ type, days/week, quantity/time

SELF-MONITORING BLOOD GLUCOSE (SMBG):

SMBG frequency: ______________________________________ (time/day, days/week)

Interpreting result: ______________________________________

HYPOGLYCAEMIA (HYPO):

Hypo in last 3 months: ☐ No ☐ Yes, frequency ____________________

Aware of symptoms: ☐ No ☐ Yes knows how to treat: ☐ Yes ☐ No

Possible contributing factors: ______________________________________

PHYSICAL MEASUREMENT	PATHOLOGY RESULT					
Weight: ______ kg	FBS/RBS	______	mmol/l	TG	______	mmol/l
Height: ______ m	HbA1c	______	%	HDL	______	mmol/l
BMI: ______ kg/m²	Creatinine	______	umol/l	LDL	______	mmol/l
Waist circumference: ______ cm	eGFR	______	ml/min/1.73m²	Cholesterol	______	mmol/l
Blood pressure: ______ mmHg	Others	______				

FOOT ASSESSMENT:

Last foot assessment: ☐ Never ☐ Yes (when)

CURRENT ISSUES related to DIABETES CARE:

____

____

____

____

DIABETES EDUCATION INTERVENTION:

Clinical Suggestions: ______________________________________

____

Self-care Education Discussed: ______________________________________

____

____

____

PLANS:

Follow-up: ☐ Yes ☐ No ____________________

Refer to: ☐ Dietitian ________ ☐ Endocrinologist ____________ ☐ Others ____________

____

Diabetes Educator

2.4.2 Follow-up Assessment Form

Name ____________________

Date of Birth ____________________

MRN ____________________

(AFFIX PATIENT LABEL HERE)

Inpatient ☐ Outpatient ☐

Date: ____________________ No. of follow up: ____________________

CURRENT DIABETES TREATMENT:

Any changes in diabetes medicines for this visit: ☐ No ☐ Yes

Type of changes: ____________________

Oral Anti-diabetic: ____________________

Insulin: ☐ Basal ____________________

☐ Bolus ____________________

☐ Premix ____________________

Others: ____________________

Adherence to medicines (MMAS-4): ☐ High adherence ☐ Medium adherence ☐ Low adherence

SELF-MONITORING BLOOD GLUCOSE (SMBG):

Hypo in last 3 months: ☐ No ☐ Yes, frequency ____________________

Aware of symptoms: ☐ No ☐ Yes knows how to treat: ☐ Yes ☐ No

Possible contributing factors: ____________________

PHYSICAL MEASUREMENT	PATHOLOGY RESULT				
Weight: _______ kg	FBS / RBS _______	mmol / l	TG	_______	mmol / l
Height: _______ m	HbA1c % ↑ / ↓ _______	%	HDL	_______	mmol / l
Blood pressure: _______ mmHg	Creatinine _______	umol / l	LDL	_______	mmol / l
	eGFR _______	ml/min/1.73m²	Cholesterol	_______	mmol / l
	Others _______				

FOOT ASSESSMENT:

Last foot assessment: ☐ Never ☐ Yes (when)

Follow-up Assessment Form

1

OUTCOME AFTER LAST DIABETES EDUCATION:

____

____

____

____

CURRENT ISSUES RELATED TO DIABETES CARE

____

____

____

____

DIABETES EDUCATION INTERVENTION:

Clinical Suggestions:

____

____

____

____

Self-care Education Discussed:

____

____

____

____

____

____

PLANS:

Follow-up: ☐ Yes ☐ No ______________

Refer to: ☐ Dietitian ______ ☐ Endocrinologist __________ ☐ Others ______________

____

Diabetes Educator

SECTION 3

HEALTHY EATING

No		Content	Page
3.1		Definition	23
3.2		Medical Nutrition Therapy (MNT) and Type 2 Diabetes Mellitus	23
3.3		General Recommendations	23
	3.3.1	Energy balance	23
	3.3.2	Carbohydrates	23
	3.3.3	Dietary fibre	24
	3.3.4	Protein	24
	3.3.5	Dietary fats and sodium	25
	3.3.6	Alcohol intake	25
3.4		Assessment	25
3.5		Goal Setting	25
3.6		Planning and Implementation	26
3.7		Evaluation and Monitoring	27
3.8		Referral for Trouble Shooting	27
3.9		References	27
3.10		Appendices	28

Healthy eating is an important part of self-care diabetes management. Recommendations for Healthy Eating should be individualized according to nutritional needs, cultural practices and willingness to change.

3.1 Definition

Healthy eating is the ability of the individual to choose a variety of foods from all food groups with suitable portions and healthier food preparation according to their diabetes treatment. (American Association of Diabetes Educators, 2011).

3.2 Medical Nutrition Therapy (MNT) and Type 2 Diabetes Mellitus

1. Individuals with diabetes should receive individualized MNT to assist in achieving blood glucose, lipids and blood pressure goals (American Diabetes Association, 2015).
2. Diabetes MNT has the greatest impact at initial diagnosis and is important at any time during the disease process (Malaysian Dietitians' Association, 2013).
3. People with diabetes especially those at high risk of complications should consult a dietitian at diagnosis and subsequent follow-up.
4. MNT should be individualized according to individual needs, cultural preferences and respecting the individual's willingness to change (refer to Section 8 on Behavioural Intervention).

3.3 General Recommendations

3.3.1 Energy balance

1. Overweight and obese individuals with diabetes should aim to lose weight 0.5- 1.0 kg per week to achieve weight loss 5-10% of their initial weight within 6 months (Malaysian Dietitians' Association, 2013).
2. This can be achieved by reducing energy intake and increasing energy output through physical activity. Individualized prescription and meal plan should be discussed with a dietitian.
3. Regular self-monitoring of weight is encouraged e.g. once a week (refer to Section 6 on Self-monitoring).

3.3.2 Carbohydrates (CHO):

1. People with diabetes need to be educated on sources and types of carbohydrates using teaching tools e.g. plate model and carbohydrate exchange list.
2. Sources of preferred CHO include wholegrain cereals, fruit, low fat dairy products and legumes.
3. CHO portions must be kept consistent on a day-to-day basis (e.g. 2-3 exchanges of cereals, grains and starchy vegetables for main meals and 1-2 exchanges per snacks) (Malaysian Dietitians' Association, 2013). Please refer to Appendix 2. Sufficient CHO should be included in the daily diet to avoid hypoglycaemia.

4. People with diabetes should limit intake of CHO from sugar sweetened drinks to reduce risk of weight gain and worsening of cardio metabolic risk profile (American Diabetes Association, 2015). This includes drinks with white sugar, brown sugar, honey, *gula Melaka* and condensed milk.
5. Non-nutritive sweeteners may be used as a substitute for caloric sweeteners to reduce overall calorie and CHO intake. Examples of non-nutritive sweeteners are aspartame, acesulfame potassium, saccharin, sucralose and stevia.

3.3.3 Dietary fibre

1. Diet high in fibre as for the normal population is recommended. Good sources of fibre include wholegrains, vegetables, fruits, legumes, seeds and nuts (Ministry of Health Malaysia, 2010).
2. Benefits of adequate fibre intake:
   - Improves blood glucose
   - Lowers total and LDL cholesterol
   - Prevents constipation
   - Controls appetite by providing fullness
3. Tips to increase fibre intake:
   - Choose whole grains products such as brown rice, wholemeal bread, oatmeal biscuits
   - Choose whole fruit instead of fruit juices
   - Include vegetables in every meal

3.3.4 Protein

1. Lean sources of protein such as lean meat or poultry, fish, lentils (e.g. dhal, chickpeas), soy products (e.g. tofu, tempeh) and low fat dairy products (milk, yogurt, cheese) are recommended.
2. Lentils, milk and yogurt contain significant amount of carbohydrate which should be taken into consideration during meal planning.
3. Protein foods should not be used to replace CHO to achieve weight loss and blood glucose control.
4. For individuals with diabetic nephropathy or diabetic kidney disease, protein restriction is necessary and dietitian referral is recommended.

3.3.5 Dietary fats and sodium

1. Total intake of fat should be limited to control body weight and improve lipid profile.
2. Low fat food preparations e.g. boiling, steaming, grilling or baking are encouraged. Deep fried foods and high fat foods should be limited.
3. Saturated fats and trans fats should be limited e.g. coconut-based products, palm oil, animal fats, butter, hard margarine, ghee, pastries.
4. Unsaturated fats are encouraged e.g. corn, sunflower, olive, canola, soy oil as a replacement for saturated fats.
5. The consumption of fatty fish (e.g. salmon, mackerel, tuna) at least two servings per week is recommended.
6. Sodium intake should be limited to less than 2300 mg daily (or 1 teaspoon of salt) (American Diabetes Association, 2015). Sources of sodium include added salt, sauces and condiments, processed food, preserved food and canned food.

3.3.6 Alcohol intake

1. If a person with diabetes chooses to drink alcohol, it should be limited to 2 drinks for men and 1 drink for women per day (Malaysian Dietitians' Association, 2013).
   Examples of 1 drink = 360ml beer / 150ml wine / 45ml hard liquor / distilled spirits.
2. If alcohol is consumed, it should be taken with meals to prevent hypoglycemia especially in individuals on insulin or insulin secretagoues.

3.4 Assessment

- Refer to Section 1 on Assessment for Anthropometry: weight, height, BMI, waist circumference.
- BMI > 23.0 kg / m^2: advise weight loss.
- BMI 18.5 to 23.0 kg / m^2: advise weight maintenance.
- Assess diet intake based on plate model, general diet pattern and food habits.
- Review frequency, causes and severity of hypoglycemia.

3.5 Goal Setting

- Able to identify CHO foods.
- Able to have consistent CHO servings per meal daily.
- Have balanced meals by choosing foods from all food groups.
- Keep regular food diary.
- Able to manage hypoglycaemia especially if on insulin therapy.

3.6 Planning and Implementation

Diagram 1: Decision Pathway for Medical Nutrition Therapy

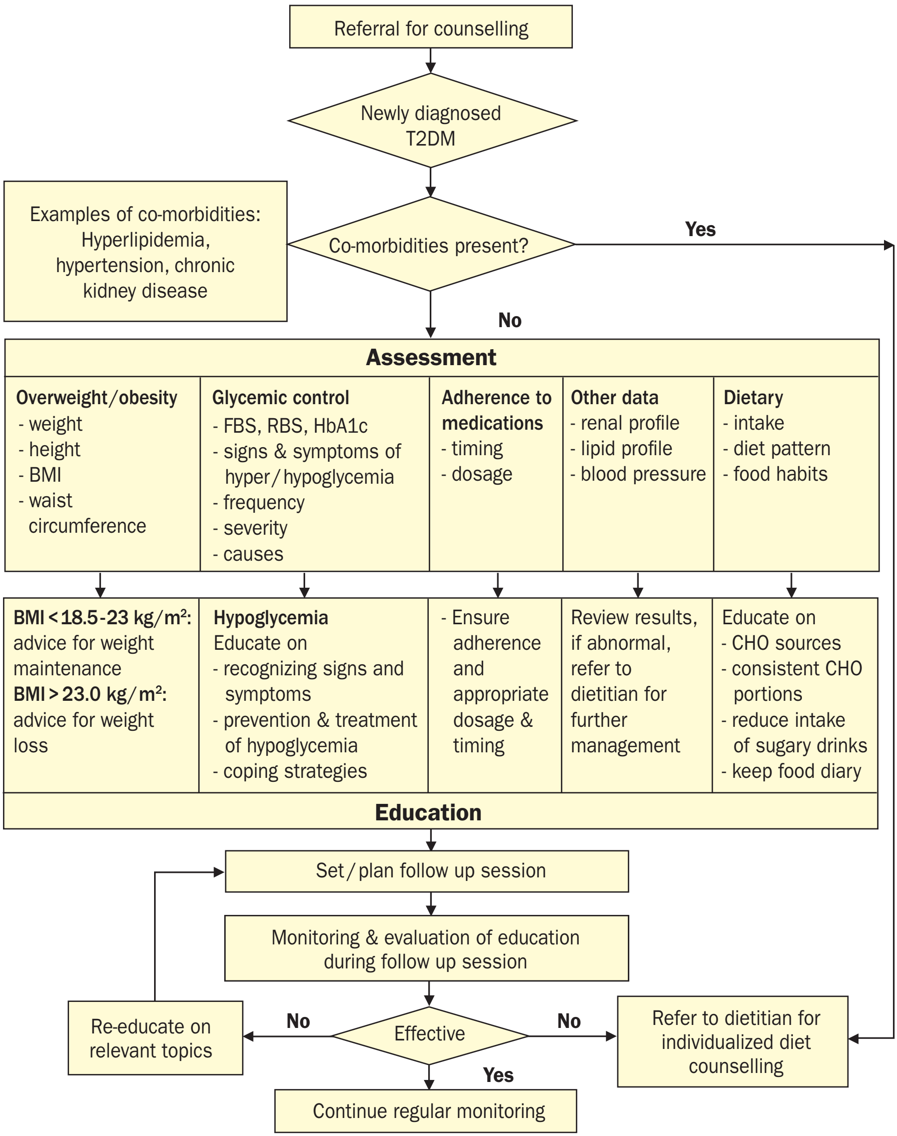

3.7 Evaluation and Monitoring

Table 1 provides a guide to monitoring the outcomes of MNT.

Table 1: Monitoring Outcomes of Medical Nutrition Therapy

No	Criteria	Yes	No
1	Maintain a reasonable body weight?		
2	Monitor blood glucose levels regularly?		
3	Eat three meals a day at consistent times?		
4	Use a meal plan to help monitor food portions?		
5	Identify foods high in carbohydrate, sugar, fats and sodium?		
6	Make appropriate food selections when dining out e.g. low sugar, low fat and high fibre food / drinks?		
7	Use sugar-free or no-added-sugar foods appropriately?		
8	Treat hypoglycaemia / hyperglycaemia appropriately?		

(Adapted from Gehling, 2001)

3.8 Referral for Trouble Shooting

Non-dietitians and Diabetes Nurse Educators should refer challenging individuals to dietitians for comprehensive MNT. These include individuals with diabetes on insulin therapy, chronic kidney disease (with or without dialysis), cancer, dyslipidemia, hypertension or any other medical condition requiring special dietary advice.

3.9 References

1. American Association of Diabetes Educators, AADE (2011) Guidelines for the Practice of Diabetes Education. *Website:* www.diabeteseducator.org. Accessed on 26 June 2015.

2. American Diabetes Association (2015) Foundations of Care: Education, Nutrition, Physical Activity, Smoking Cessation, Psychosocial Care and Immunization. Sec. 4. In Standards of Medical Care in Diabetes - 2015. *Diabetes Care* Vol. 38 (Suppl.1), S20-S30.

3. Gehling E. (2001) Lippincott's Case Management, Vol. 6, No. 1. 2-12, Lippincott Williams & Wilkins, Inc. *Website:* http://www.nursingcenter.com. Accessed on 15 June 2015.

4. Ministry of Health Malaysia (2010) Malaysian Dietary Guidelines, *Website:* http://www2.moh.gov.my/. Accessed on 17 January 2016.

5. Malaysian Dietitians' Association (2013) Medical Nutrition Therapy Guidelines for Type 2 Diabetes Mellitus (2nd Edition).

3.10 Appendices

Appendix 1: Plate model

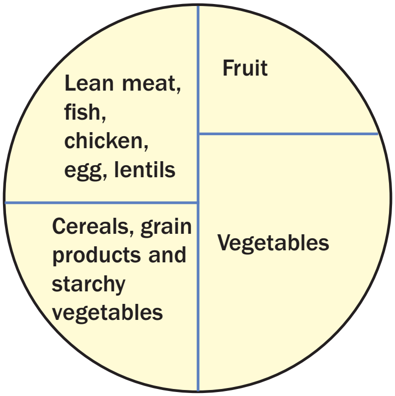

Appendix 2: Carbohydrate Exchange Lists

Cereals, grain products and starchy vegetables

Each item contains 15 g carbohydrate, 2.0 g protein, 0.5 g fat and 75 calories

Rice, white unpolished (cooked)	½ cup or ⅓ chinese rice bowl
Rice porridge	1 cup
Kway teow, mee hoon, tang hoon, spaghetti, macaroni	½ cup or ⅓ chinese rice bowl
Mee, wet	⅓ cup
Idli	1 piece (60 g)
Putu mayam	1 piece (40 g)
Thosai, diameter 20 cm	½ piece
Chappati, diameter 20 cm	⅓ piece
Bread (wholemeal, high fibre, white/brown), plain roll	1 slice (30 g)
Burger bun, pita bread (6”)	½ piece
Oatmeal, cooked	¼ cup
Oats, uncooked	3 rounded tablespoons
Muesli	¼ cup
Flour (wheat, rice, atta)	3 rounded tablespoons
Biscuits (plain, unsweetened) e.g. cream crackers, Ryvita	3 pieces
Small thin, salted biscuits (4.5 x 4.5 cm)	6 pieces

**Starchy vegetables**	
*Baked beans, lentils (*Contains more protein than other foods in the list i.e. 5 g / serve)	⅓ cup
Corn kernel, peas (fresh / canned)	½ cup
Sweet potato, tapioca, yam	½ cup (45 g)
Breadfruit (sukun)	1 slice (75 g)
Pumpkin	1 cup (100 g)
Corn on the cob, 6 cm	1 small
Potato	1 small (75 g)
Potato, mashed	½ cup
Water chestnut	4 pieces
1 cup is equivalent to 200 ml in volume, 1 cup = ¾ chinese rice bowl (11.2 cm diameter x 3.7 cm deep).<br>Tablespoon refers to dessertspoon level (equivalent to 2 teaspoons)	

Fruit

Each item contains 15 g carbohydrate and 60 calories

Banana	1 small (60 g)
Apple, orange, custard apple (*buah nona*)	1 medium
Star fruit, pear, peach, persimmon, *ciku*, kiwi	
Hog plum (*kedondong*)	6 whole
Mangosteen, plum	2 small
Duku langsat, grapes, longan	8 pieces
Water apple (*jambu air*), small	
Lychee, *rambutan*	5 whole
Pomelo	5 slices
Papaya, pineapple, watermelon, honeydew, soursop	1 slice
Guava	½ fruit
Cempedak, nangka	4 pieces
Prunes	3 pieces
Dates (*kurma*)	2 pieces
Raisin	20 g
Durian	2 medium seeds
Mango	½ small

Milk

Fresh cow's milk, UHT milk	1 cup (240 ml)
Powdered milk (skim, full cream)	4 rounded tablespoons or ⅓ cup
Yogurt (plain/low fat)	¾ cup
Evaporated (unsweetened)	½ cup

	CHO (g)	Protein (g)	Fat (g)	Energy (kcal)
Skimmed (1% fat)	15	8	trace	90
Low fat (2% fat)	12	8	5	125
Full cream	10	8	9	150

(Adapted from Malaysian Dietitians' Association, 2013)

SECTION 4

PHYSICAL ACTIVTY AND EXERCISE

No		Content	Page
4.1		Definition	32
	4.1.1	Physical Activity	32
	4.1.2	Exercise	32
4.2		Benefits of Regular Physical Activity in Type 2 Diabetes Mellitus	33
4.3		General Recommendations	33
4.4		Special Considerations	34
4.5		Assessment	34
4.6		Goal Setting	35
4.7		Planning	36
4.8		Implementation	37
4.9		Evaluation and Monitoring	37
4.10		Referral for Trouble Shooting	37
4.11		References	38
4.12		Appendices	40

Physical activity and exercise are important for people with diabetes to control weight, improve glucose control and overall health and wellbeing. Types and duration of physical activity or exercise should be individualized depending on the individual's medical condition.

4.1 Definition

4.1.1 Physical Activity

- Any bodily movement produced by skeletal muscles that results in energy expenditure (Carsperson et al, 1985).
- Physical activity in daily life can be categorized into occupational, sports, conditioning, household or other activities (Carsperson et al, 1985).

4.1.2 Exercise:

A subset of physical activity that is planned, structured, repetitive and has as a final or an intermediate objective the improvement or maintenance of physical fitness. 'Physical activity' and 'exercise' are used interchangeably in this document (Carsperson et al, 1985).

Aerobic Exercise

This consists of rhythmic, repeated, and continuous movements of the same large muscle groups for at least 10 minutes at a time. Examples include brisk walking, cycling and jogging (Sigal et al, 2004 Appendix 1).

Moderate intensity exercise: Exercise causes

- Noticeable increase in breathing rate at which a conversation can be maintained but does not have enough breath to sing (Health Promotion Board, 2011).
- 50 to 70% of maximum heart rate (maximum heart rate = 220 - age) (Sigal et al, 2004).

Vigorous intensity exercise: Exercise causes

- Large increase in breathing rate - one is not able carry on a conversation but is not out of breath (Health Promotion Board, 2011).
- More than 70% of maximum heart rate (Sigal et al, 2004).

Resistance Exercise

- Activities that use muscular strength to move a weight or work against a resistive load. When performed with regularity and moderate to high intensity, resistance exercise increases muscular fitness (Golberg et al. 2010; American College of Sports Medicine 2010).
- Examples include push-ups, sit-ups, weight lifting and exercises using weight machines (Golberg et al, 2010; American College of Sports Medicine, 2010).

4.2 Benefits of Regular Physical Activity in Type 2 Diabetes Mellitus

- Improves metabolic control (glucose control, increased insulin sensitivity, decreased HbA1c, and decreased insulin requirements).
- Improves blood pressure and lipid profile.
- Promotes weight loss with Medical Nutrition Therapy (Golberg et al, 2010).

4.3 General Recommendations

Appropriate frequency, intensity, duration, mode of exercise should be individualized based on personal condition.

<u>Aerobic exercise</u>

1. People without diabetes complications should have regular, moderate intensity exercise (Appendix 1).
   a. For a total duration of at least 150 minutes per week in bouts of 10 minutes or more (Tremblay et al, 2011) spread across the week; or
   b. Minimum 5 times per week of at least 30 minutes per session or 75 minutes per week of vigorous-intensity aerobic physical activity (American Diabetes Association, 2015).
2. People with diabetes should have no more than 2 consecutive days without exercise.
3. Physical activity benefits can be achieved at one session or accumulated over the day (e.g. 3 x 10 minutes sessions in a day or 30 minutes session) (Eckel et al, 2013).
4. Higher levels of physical activity, 200-300 minutes per week are recommended to maintain weight lost or minimize weight regain in the long term (Obesity Expert Panel Report, 2013).
5. Exercise should be started based on individual fitness level.

<u>Resistance exercise</u>

1. Resistance exercise of at least 2 -3 days/week with each session consisting of 2-4 sets of 8-12 repetitions is recommended in addition to aerobic exercise (Eckel et al, 2013).

Other types of exercises

1. Milder forms of exercise (e.g. yoga, tai chi) may have additional benefit but these should not be in replacement of other recommended types of exercise.
2. Walk more often with a goal in mind (e.g. 10,000 steps a day) which can be monitored using a pedometer/step counter (Qiu et al, 2014). 10,000 steps (~8 kilometers or 5 miles), burns 300 to 400 calories and may be achieved with an active lifestyle that includes a 30-minute walk each day (Choi et al, 2007) (Refer to Appendix 3 for the Daily pedometer step count category).

4.4 Special Considerations

The following conditions require further assessment before embarking on exercise:

- Cardiovascular disease
- Retinopathy
- Neuropathy
- Foot ulceration or injury
- Hypoglycemia i.e. when individuals show signs and symptoms of shakiness, weakness, abnormal sweating, nervousness, anxiety, tingling of the mouth and fingers, and hunger)

Caution is needed in patients with the following conditions.

- When exercising with blood glucose levels >16.7 mmol/L, check for ketone bodies and ensure individual is feeling well and is adequately hydrated.
- For individuals using insulin or insulin secretagogues, medication doses may need to be reduced or extra carbohydrate intake may be needed before or during physical activity to prevent exercise associated hypoglycaemia (Golberg et al, 2010; Ministry of Health, 2015; Obesity Expert Panel Report, 2013; Eckel et al, 2013).

4.5 Assessment

Assessment before the recommendation of physical activity

1. Risk stratification
    a. Physical activity readiness questionnaire (PAR-Q)(Appendix 4)
    b. Fitness assessment
        i. Anthropometry (body mass index & waist circumference)
        ii. Fitness Test (6 Minutes Walk Test, step test etc.)
    c. Diabetes treatment modality
    d. Metabolic control (e.g. blood glucose, blood pressure etc.)
    e. Diabetes complications (refer to special consideration & Appendix 2)
2. Readiness for behaviour change (refer to Section 8 on Behavioural Intervention)
3. Extra questions on history of physical activity:
    a. Hobbies or leisure activities
    b. Current exercise (yes or no)
    c. Types, duration & frequency
    d. Success &/or failure of previous physical activity/exercise efforts

Sample questions

- How many times a week do you usually do 20 minutes or more of vigorous-intensity physical activity that makes you sweat or puff and pant? (e.g., heavy lifting, digging, jogging, aerobic or fast bicycling).

  ☐ 3 or more times a week ☐ 1 to 2 times a week ☐ None

- How many times a week do you usually do 30 minutes or more of moderate-intensity physical activity or walking that increases your heart rate or makes you breathe harder than normal? (e.g., carrying light loads, bicycling at a regular pace, or doubles tennis).

  ☐ 5 or more times a week ☐ 3-4 times a week ☐ 1-2 times a week ☐ None

  (Web source: *http://appliedresearch.cancer.gov/tools/paq/q101.htm*)

4. Identify potential barriers to behaviour change (Refer to sample questionnaire for assessment in Appendix 4)

4.6 Goal Setting

- Increase physical activity while performing daily activities.
- Increase duration, intensity and frequency of exercise.
- Identify support and community resources e.g. home setting/environment, parks, workplace exercise facilities.
- Prioritise physical activity goals based upon assessment and preference.
- Use behavioural change methodology to influence people with diabetes to increase physical activity and exercise.
- Set individual behavioural physical activity goal (American Association of Diabetes Educators, 2011; American Society of Bariatric Physicians, 2013; Canadian Society for Exercise Physiology, 2014).

4.7 Planning

Diagram 1: Decision Pathway for Exercise

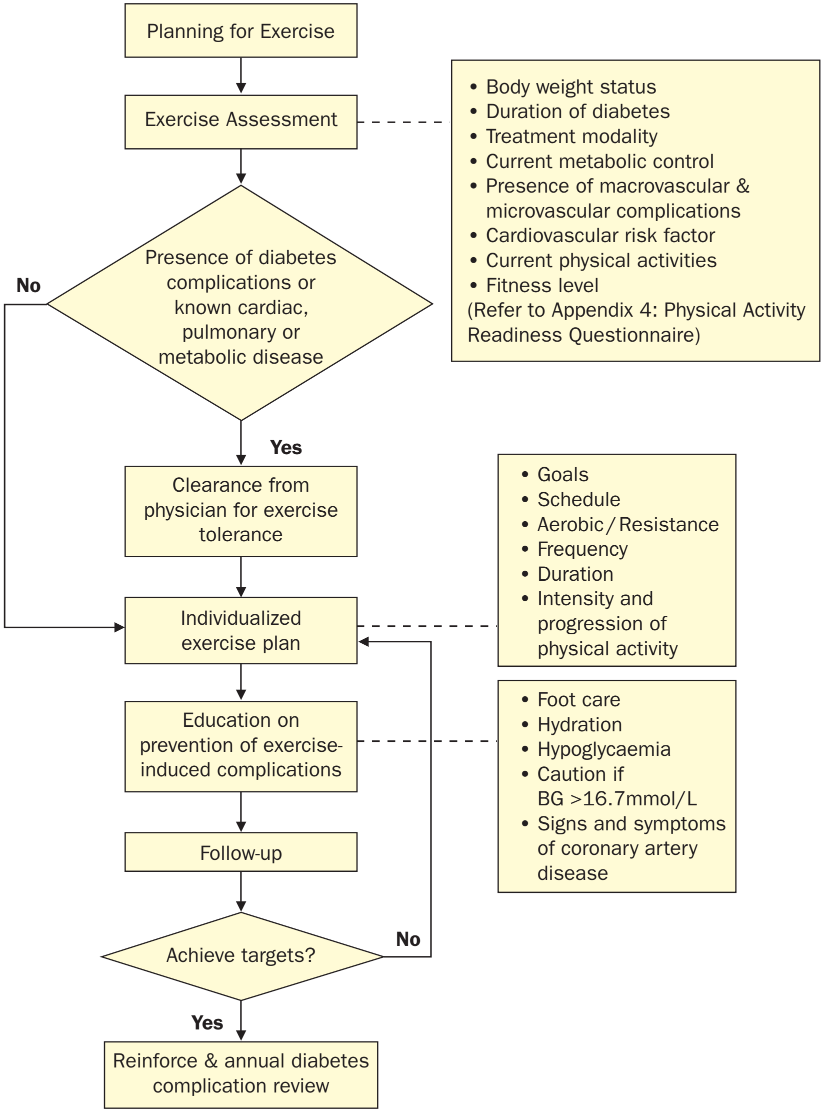

4.8 Implementation

To provide supportive tools and reference on how to use it/rationale for use, e.g. use of pedometer and step counting, phone apps e.g. Walk Star (iPhone); Walking Mate (Android), exercise diary.

- Refer/support diabetes management skill training, and offer guidance on accessing care.
- Recommend support groups or community physical activity.
- Recommend and execute plans, ensuring that the person with diabetes has the required knowledge, skills and resources.
- Identify and address barriers that become evident throughout the process (American Association of Diabetes Educators, 2011).

4.9 Evaluation and Monitoring

Table 2 provides an evaluation checklist for physical activity and exercise.

Table 2: Evaluation Checklist

1	**Knowledge on exercise safety**
	• Proper attire and sufficient hydration<br>• Foot care – proper foot wear for exercise, foot examination post exercise<br>• Safe level of blood glucose for exercise<br>• Stop exercise if there is chest discomfort, dizziness, sharp or significant muscle pain or symptoms of hypoglycaemia
2	**Achievement of people with diabetes on physical activity plan**
	• Logbook/monitoring tool, achievement level, challenges. Refer Section 8 on behaviour modification evaluation
3	**Tolerance of people with diabetes on physical activity**
	• Cardiac discomfort<br>• Hypoglycaemia

4.10 Referral for Trouble Shooting

1. Sports physician or Endocrinologist regarding patient's medical problems.
2. Physiotherapist/exercise physiologist/physical trainer for exercise techniques and safety of certain exercise.

4.11 References

1. American Association of Diabetes Educators (2011) Guidelines for the Practice of Diabetes Education. Website: www.diabeteseducator.org. Accessed on 18 November 2013.

2. American Diabetes Association (2015) Foundations of Care: Education, Nutrition, Physical Activity, Smoking Cessation, Psychosocial Care and Immunization, *Diabetes Care* Vol. 38 (Suppl. 1), S20 - S30.

3. American Society of Bariatric Physicians, ASBP (2013) Adult Adiposity Evaluation and Treatment. *Website: www.obesity algorithm.org.* Accessed on 20 January 2014.

4. Ainsworth, B., Haskell, W.L., Whitt, M.C. et al (2000) Compendium of Physical Activities: An Update of Activity Codes and MET Intensities, *Medicine and Science in Sports and Exercise* Vol. 32 (9), S498 - S516.

5. Canadian Society for Exercise Physiology (2014) Physical Activity Readiness Questionnaire. Website: http://www.csep.ca/PAR-QForms. Accessed on 20 March 2014.

6. Carsperson, C.J., Powell, K.E. and Christenson, G.M. (1985) Physical Activity, Exercise, and Physical Fitness: Definitions and Distinctions for Health-Related Research. *Public Health Reports* Vol. 100 (2), 126 - 131.

7. Choi, B.C.K., Pak, A.W.P., Choi, J.C.L et al (2007) Daily Step Goal of 10,000 Steps: A Literature Review. *Clinical Investigation Medicine* Vol. 30(3), E146 - E151.

8. Eckel, R.H., Jakicic, J.M., Ard, J.D. et al (2013) AHA/ACC Guideline on lifestyle management to reduce cardiovascular risk. *Journal of the American College of Cardiology* doi: 10.1016/j.jacc.2013.11.003.

9. Golberg, S., Sigal, R.J., Fernhall, B. et al (2010) Exercise and Type 2 Diabetes: The American College of Sports Medicine and the American Diabetes Association: Joint Position Statement. *Diabetes Care* Vol.33, e147 - e167.

10. Health Promotion Board, Singapore (2011) National Physical Activity Guidelines: Professional Guide, Website: https://www.ntu.edu.sg/has/SnR/Documents/NPAG_Professional_Guide.pdf. Accessed on 28 September 2015.

11. Ministry of Health Malaysia (2015) *Clinical Practice Guidelines for Management of Type 2 Diabetes Mellitus* (5th Edition).

12. Obesity Expert Panel Report (2013), Circulation, Website: http://www.circ.ahajournals.org/content/early/2013/11/11/01.cir.0000437739.71477.ee/suppl/DC1. Accessed on 20 January 2014.

13. Qiu, S., Cai, X., Chen, X. et al (2014) Step Counter Use in Type 2 Diabetes: A Meta-Analysis of Randomized Controlled Trials. *BMC Medicine*; 12 - 36.

14. Sigal, R.J., Wasserman, D.H., Kenny, G.P. et al (2004) Physical Activity / Exercise and Type 2 Diabetes. *Diabetes Care* Vol. 27, 2518 - 2538.

15. Tremblay, M.S., Warburton, D.E.R., Janssen, I. et al. (2011) New Canadian Physical Activity Guideline. *Applied Physiology Nutrition Metabolism* Vol. 36, 36 - 46.

16. Tudor-Locke, C. and Bassett, D.R. (2004) How Many Steps Per Day Are Enough? Preliminary Pedometer Indices for Public Health. *Sports Medicine* Vol. 34, 1 - 8.

4.12 Appendices

Appendix 1: Example of Aerobic Physical Activity Based on Intensity

Moderate intensity physical activity	Vigorous intensity physical activity
Brisk walking<br>Bicycling 8 - 15 km/hour<br>Line dancing<br>Traditional dancing (e.g. *Joget, Mak Yong*)<br>Table tennis – competitive<br>Tennis<br>Badminton – competitive<br>Volley ball<br>Golf<br>Netball	Race walking<br>Jogging or running<br>Walking and climbing briskly uphill<br>Bicycling more than 15 km/hour<br>Aerobic dancing – high impact<br>Teaching an aerobic dance class<br>Basketball<br>Squash<br>Sepak takraw<br>Swimming – steady paced laps<br>Scuba diving

(Adapted from Ainsworth et al., 2000)

Appendix 2: Diabetes Complication Assessment Related to Exercise

Are complications present?
• Known to have or at risk of CVD<br>• At risk for injury due to peripheral neuropathy<br>• Known to have renal disease (high intensity aerobic or resistance exercise may worsen the progression)<br>• Retinopathy (may be worsened by activities that increase ocular pressure)

(Adapted from American College of Sports Medicine, 2010)

Appendix 3: Daily Pedometer Step Count Categories

Range of Daily Step Counts (steps per day)	Activity Classification
<5000	Sedentary lifestyle index
5000 - 7499 as typical day excluding sports/ exercise	Low active
7500 - 9999 include some volitional activities (and/elevated occupational activity demands)	Somewhat active
≥ 10000	Active
> 12500	Highly active

(Adapted from Tudor-Locke & Bassett, 2004; Tudor-Locke, 2010)

Appendix 4: Physical Activity Readiness Questionnaire (PAR-Q)

AIM: To identify those individuals with a known disease, signs or symptoms of disease, who may be at a higher risk of an adverse event during physical activity/ exercise. This stage is self-administered and self-evaluated.

Regular physical activity is fun and healthy and increasingly more people are starting to become more active every day. Being more active is very safe for most people. However, some people should check with their doctor before they start becoming much more physically active.

If you are planning to become much more physically active than you are now, start by answering the seven questions in the box below. If you are between the ages of 15 and 69, the PAR-Q will tell you if you should check with your doctor before you start. If you are over 69 years of age, and you are not used to being very active, check with your doctor.

Common sense is your best guide when you answer these questions. Please read the questions carefully and answer each one honestly: check YES or NO.

Name: ________________________________ Age : _________

Gender: Male ☐ Female ☐

Please circle a response for each question.

1	Has your doctor ever told you that you have a heart condition or have you ever suffered a stroke?	Yes	No
2	Do you ever experience unexplained pains in your chest at rest or during physical activity/exercise?	Yes	No
3	Do you ever feel faint or have spells of dizziness during physical activity/ exercise that causes you to lose balance?	Yes	No
4	Have you had an asthma attack requiring immediate medical attention at any time over the last 12 months?	Yes	No
5	If you have diabetes (Type 1 or Type 2) have you had trouble controlling your blood glucose in the last 3 months?	Yes	No
6	Do you have any diagnosed muscle, bone or joint problems that you have been told could be made worse by participating in physical activity/exercise?	Yes	No
7	Do you have any other medical condition(s) that may make it dangerous for you to participate in physical activity/exercise?	Yes	No

If you answered ‘Yes’ to any of the 7 questions, please seek guidance from your GP or appropriate allied health professional prior to undertaking physical activity/ exercise.

If you answered ‘No’ to all 7 questions, and you have no other concerns about your health, you may proceed to undertake light-moderate intensity physical activity/ exercise

(Adapted from Canadian Society for Exercise Physiology, 2014)

SECTION 5

MEDICATION

No		Content	Page
5.1		Definition	43
5.2		General Recommendations	43
5.3		Oral Anti-Diabetic (OAD) Agents	44
		Combination of Oral Anti-Diabetic (OAD) Agents	48
5.4		Algorithm for Oral Medication	49
5.5		Tips to Overcome Non-adherence	50
5.6		Glucagon-like Peptide-1 (GLP-1) Receptor Agonists	51
	5.6.1	Step-by-step Injection Technique (GLP-1 Receptor Agonist)	52
5.7		Algorithm for Insulin	54
5.8		Barriers to Initiate Insulin Therapy	55
5.9		Practical Guide to Insulin Injection	56
	5.9.1	Step-by-step Injection Technique (Insulin)	56
	5.9.2	Injection Problems and Solutions	59
	5.9.3	Insulin Storage	60
5.10		Pharmacokinetic Profile of Insulins	61
5.11		Commonly used Antihypertensives in Diabetes	62
5.12		Dyslipidaemia Therapy in Diabetes	64
5.13		Antiplatelet Therapy in Diabetes	64
5.14		Drugs That May Cause Hyperglycaemia	65
5.15		Drugs that May Cause Hypoglycaemia	66
5.16		Assessment	67
5.17		Goal Setting	67
5.18		Planning	68
5.19		Implementation	68
5.20		Evaluation and Monitoring	69
	5.20.1	Insulin Administration Checklist	69
	5.20.2	Oral Anti-Diabetic Agent Checklist	70
5.21		References	71

Medication for Type 2 Diabetes

In type 2 diabetes, hyperglycaemia occurs due to a combination of pathophysiological defects such as:

1. Insufficient insulin secretion from the pancreas
2. Resistance to insulin action in liver, fat and muscle cells
3. Decreased incretin secretion from small intestinal cells causing non-suppression of glucagon

Medication is targeted at these different areas in an attempt to normalize blood glucose levels.

5.1 Definition:

Glucose lowering medications (oral and injections) approved for use in people with Type 2 diabetes in Malaysia.

- Medications which improve blood glucose control
- Medications which improve other risk factors contributing to morbidity & mortality of people with diabetes

5.2 General Recommendations

Classes of medication which lower blood glucose levels

- Medications which reduce carbohydrate absorption from the gut
- Medications which increase insulin secretion/production from the pancreas
- Medications which improve insulin sensitivity
- Medications which increase incretin levels
- Medications which reduce glucose reabsorption in the kidney
- Exogenous Insulin

Some of these medications are available in combination tablets. Due to their different modes of action and side effects, people with diabetes who take these medications (and those who advise them) need to be aware of the importance of timing, dosage and other relevant factors.

5.3 Oral Anti-Diabetic (OAD) Agents (American Diabetes Association, 2015)

Drug Name	Recommended dose	Physiological Actions	Advantages	Disadvantages	Administration
**1. Medications which reduce glucose absorption from the gut**					
**α-glucosidase Inhibitors (AGIs)**					
Acarbose<br>50 mg / 100 mg tablet	Initial dose: 50 mg OD<br>Max dose: 100 mg TDS	Slows absorption of complex carbohydrates	• No hypoglycaemia<br>• ↓ Postprandial glucose excursions<br>• Non systemic	• Gastrointestinal side effects (flatulence, diarrhoea)<br>• Frequent dosing schedule	Take with 1st bite of each main meal
**2. Medications which increase insulin secretion / production from the pancreas**					
**a) Sulphonylureas**					
Gliclazide 80 mg tablet	Initial dose: 40 mg OM<br>Max dose: 160 mg BD	↑ Insulin secretion	• Extensive experience	• Hypoglycaemia<br>• Newer sulphonylureas (eg. Gliclazide MR and Glimepiride) have less risk of hypoglycaemia<br>• Weight gain	Take before meals
Glibenclamide 5 mg tablet	Initial dose: 2.5 mg OM<br>Max dose: 10 mg BD				
Glipizide 5 mg tablet	Initial dose: 2.5 mg OM<br>Max dose: 10 mg BD				
Gliclazide MR<br>30 mg / 60 mg tablet	Initial dose: 30 mg OM<br>Max dose: 120 mg OM				Take immediately before 1st meal of the day
Glimepiride 2 mg / 3 mg tablet	Initial dose: 1 mg OM<br>Max dose: 6 mg OM				

Drug Name	Recommended dose	Physiological Actions	Advantages	Disadvantages	Administration
**b) Meglitinides**					
Repaglinide 1 mg / 2 mg tablet	Initial dose: 0.5 mg with main meals<br>Max dose: 4 mg with main meals (≤ 16 mg daily)	↑ Insulin secretion	• ↓ Postprandial glucose excursions<br>• Dosing flexibility	• Hypoglycaemia<br>• Weight gain<br>• Multiple dosing	Take before meals
Nateglinide 120 mg tablet	Initial dose: 60 mg with main meals<br>Max dose: 120 mg with main meals (≤ 360 mg daily)				
**3. Medications which improve insulin sensitivity**					
**a) Biguanides**					
Metformin 500 mg tablet	Initial dose: 500 mg OD<br>Max dose: 1 g BD	• ↓ Hepatic glucose production<br>• ↑ Peripheral glucose uptake	• Extensive experience<br>• No weight gain<br>• No hypoglycaemia<br>• Mild reduction in cholesterol	• Gastrointestinal side effects (diarrhoea, abdominal cramps)<br>• Lactic acidosis risk (rare)<br>• Vitamin B_{12} deficiency<br>• Multiple contraindications: CKD eGFR < 30 mL / min, acidosis, hypoxia, dehydration, etc.	Take after meals
Metformin retard 850 mg tablet (slow release formulation)	Initial dose: 850 mg OD<br>Max dose: 1700 mg OM / 850 mg ON				
Metformin XR extended release 500 mg / 750 mg / 1000 mg tablet	Initial dose: 500 mg ON<br>Max dose: 2 g ON		Less GI symptoms		Take with evening meal

Drug Name	Recommended dose	Physiological Actions	Advantages	Disadvantages	Administration
**b) Thiazolidinediones**					
Pioglitazone<br>15 mg / 30 mg tablet	Initial dose: 15 mg OD<br>Max dose: 45 mg OD	• ↑ Insulin action in periphery<br>• ↑ Glucose utilization by muscle and fat tissue<br>• ↓ Hepatic glucose output	• No hypoglycaemia<br>• Slight reduction in TG	· Weight gain<br>· Oedema / fluid retention<br>· Bone fractures (in older women)<br>· Do not use in those with symptomatic HF or class III or IV HF<br>· Contraindicated in heart failure or those at risk for heart failure	Take with or without meal
Rosiglitazone<br>4 mg / 8 mg tablet	Initial dose: 4 mg OD<br>Max dose: 8 mg OD				
**4. Medications which increase incretin levels**					
**a) DPP-4 inhibitor**					
Sitagliptin<br>25 mg / 50 mg / 100 mg tablet	Initial dose: 50 mg OD<br>Max dose: 100 mg OD	• ↑ Insulin secretion<br>• ↓Post-prandial glucagon levels	• No hypoglycaemia<br>• No weight gain	• Urticaria / angioedema<br>• Rare report of pancreatitis<br>• Requires dose adjustment in renal insufficiency except for Linagliptin	Take with or without meal
Vildagliptin 50 mg tablet	Initial dose: 50 mg OD<br>Max dose: 100 mg OD				
Linagliptin 5 mg tablet	Initial and max dose: 5 mg OD				
Saxagliptin<br>2.5 mg / 5 mg tablet	Initial dose: 2.5 mg OD<br>Max dose: 5 mg OD				
Alogliptin 6.25 mg / 12 mg / 25 mg	Initial dose: 6.25 mg OD<br>Max dose: 25 mg OD				

Drug Name	Recommended dose	Physiological Actions	Advantages	Disadvantages	Administration
**5. Medications which reduce the reabsorption of glucose at kidney**					
**Sodium-glucose cotransporter 2 (SGLT 2) inhibitor**					
Dapagliflozin<br>5 mg / 10 mg tablet	Initial dose: 5 mg OD,<br>Max dose: 10 mg OD	Inhibiting SGLT2 in the proximal renal tubules, ↓ reabsorption of filtered glucose from the tubular lumen, result in increased urinary excretion of glucose	• Weight loss	• Increased risk of UTI<br>• Increased risk of genitourinary tract infection<br>• Contraindicated in those with severe renal impairment as its MOA requires a functional kidney.	Take with or without meal
Empagliflozin<br>10 mg / 25 mg tablet	Initial dose: 10 mg OD,<br>Max dose: 25 mg OD				
Canagliflozin 100 mg / 300 mg tablet	Initial dose: 100 mg OD<br>Max dose: 300 mg OD				

Combination of Oral Anti-Diabetic (OAD) Agent

Combination (Brand Name)	Dose per tablet	Minimum and Max dose
Metformin and Glibenclamide (Glucovance)	500 mg / 2.5 mg tablet<br>500 mg / 5 mg tablet	Min dose: 500 mg / 2.5 mg BD<br>Max dose: 1000 mg / 10 mg BD
Metformin and Glimepiride (Amaryl M SR)	500 mg / 2 mg tablet	Min dose: 500 mg / 2 mg OD<br>Max daily dose: 2000 mg / 8 mg
Rosiglitazone and Metformin (Avandamet)	2 mg / 500 mg tablet<br>4 mg / 500 mg tablet<br>4 mg / 1000 mg tablet	Min dose: 2 mg / 500 mg OD<br>Max dose: 4 mg / 1000 mg BD
Sitagliptin and Metformin (Janumet)	50 mg / 500 mg tablet<br>50 mg / 850 mg tablet<br>50 mg / 1000 mg tablet	Min dose: 50 mg / 500 mg BD<br>Max dose: 50 mg / 1000 mg BD
Vildagliptin and Metformin (Galvus-Met)	50 mg / 500 mg<br>50 mg / 850 mg<br>50 mg / 1000 mg	Min dose: 50 mg / 500 mg BD<br>Max dose: 50 mg / 1000 mg BD
Linagliptin and Metformin (Trajenta Duo)	2.5 mg / 500 mg<br>2.5 mg / 850 mg<br>2.5 mg / 1000 mg	Min dose: 2.5 mg / 500 mg BD<br>Max dose: 2.5 mg / 1000 mg BD
Saxagliptin and Metformin XR (Kombiglyze XR)	2.5 mg / 1000 mg<br>5 mg / 500 mg<br>5 mg / 1000 mg	Min dose: 2.5 mg / 1000 mg OD or 5 mg / 500 mg OD<br>Max dose: 5 mg / 2000 mg OD

5.4 Algorithm for Oral Medication

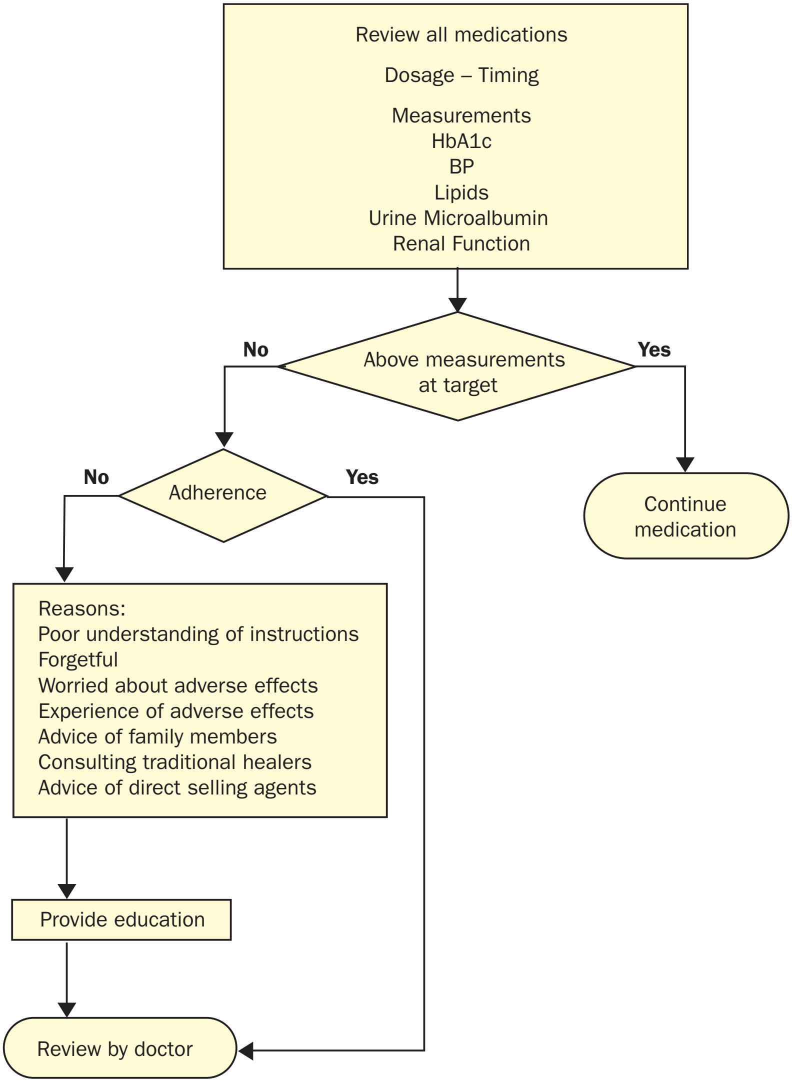

5.5 Tips to Overcome Non-adherence

Problem	Solution
Forgetful	Suggest the use of pillbox or hand phone alarm as reminder.
Worried of side effects of medicine	Explain that benefits outweigh risks hence the doctor has started him/her on this medicine. Share complications of diabetes mellitus.
Experience of adverse effects	Explain common side effects of each medicine and how to prevent or minimize them, e.g. take metformin after meals instead of before to avoid GI symptoms, take gliclazide with or before meal to prevent hypoglycaemia. Discuss with doctor regarding medication adjustment if a particular medication cannot be tolerated.
Poor understanding of medicine	Ensure understanding of dose, indication, frequency and time of administration of each medication. We may use tools like drawing a table, or pictures.
Taking traditional medicines	Explain risk of adulteration. If diabetes control or risk factors are not at target, explain the efficacy of modern medicine and long term complications of poorly controlled diabetes.
Ignorance/Apathetic	Motivate to take responsibility for own health.

5.6 Glucagon-like Peptide-1 (GLP-1) Receptor Agonists (American Diabetes Association, 2015)

Drug Name	Recommended dose	Physiological Actions	Advantages	Disadvantages	Administration
Exenatide<br>IR (Immediate release)<br>5 µg / 20 µl<br>10 µg / 40 µl	Immediate release<br><br>Min dose: 5 µg BD<br>Max dose:10 µg BD	• ↑ glucose-dependent insulin secretion<br>• ↓ inappropriate glucagon secretion<br>• slows gastric emptying	• No hypoglycaemia<br>• Weight loss	• Nausea, vomiting, diarrhoea are common side effects	Given subcutaneously
Exenatide<br>XR (Extended release) 2 mg	Extended release:<br><br>Min dose: 2 mg once weekly<br>Max dose: 2 mg once weekly				
Liraglutide<br>6 mg / ml	Initial dose: 0.6 mg OD<br>Max dose: 1.8 mg OD				
Lixisenatide<br>50 µg / ml<br>100 µg / ml	Min dose: 10 µg OD<br>Max dose: 20 µg OD				

For barriers to initiate injectables, please refer to section 5.8.3.

5.6.1 Step-by-step Injection Technique (GLP-1 Receptor Agonist)

The injection technique for Exenatide IR (Immediate Release) and Liraglutide are similar to insulin injection technique. Refer to section 5.9.1 for insulin injection technique. However, they do not require priming (step 6 and 7 in section 5.9.1).

The injection technique for Exenatide XR (Extended Release) involves 3 steps:
1. Prepare
2. Mix
3. Inject

1. Prepare

i.) Remove one pen from the refrigerator. Wait for 15 minutes. Medicine at room temperature is easily mixed well.

ii) Attach the needle to the pen and do not remove the needle cover. (see Picture A)

iii) Combine the medicine by holding the pen upright and slowly turning the knob. Stop when you hear the click and the green label disappears. (see Picture B)

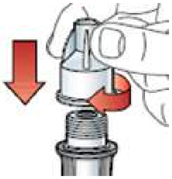

Picture A

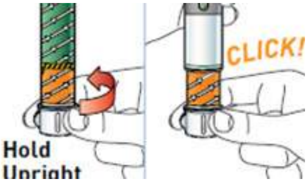

Picture B

(Image source: AstraZeneca)

2. Mix

i) Hold the pen by the end with the orange label and tap the pen firmly against the palm of your hand to mix. Rotate the pen every ten taps. (see Picture C)

ii) Hold your pen up to the light and look through both sides of the mixing window to make sure the medicine is mixed well. (see Picture D)

* To get your full dose, the medicine must be mixed well. If not mixed well, tap longer and more firmly.

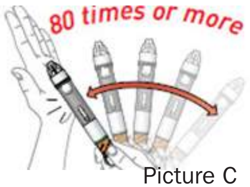

Picture C

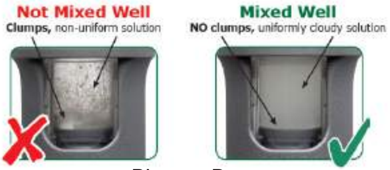

Picture D

(Image source: AstraZeneca)

3. Inject

i) Twist the knob until the injection button is released. (see Picture E)

ii) Pull the needle cover straight off. For Injection technique, please refer to section 5.9.1 no. 11 and 12.

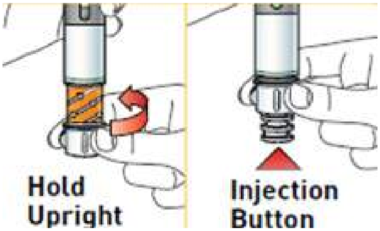

Picture E
(Image source: AstraZeneca)

5.7 Algorithm for Insulin

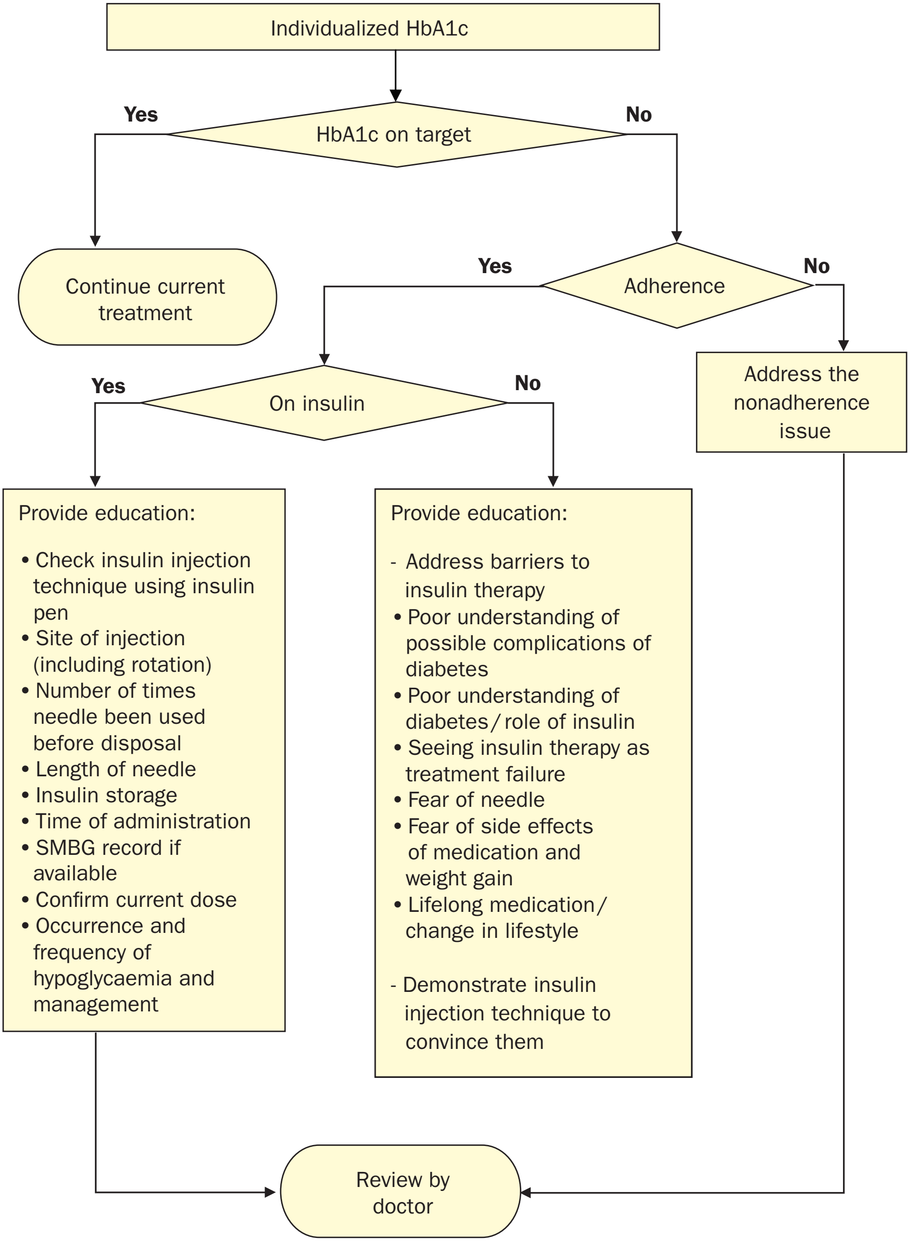

5.8 Barriers to Initiate Insulin Therapy

Insulin production by the pancreas decreases with age and duration of diabetes so that many people with Type 2 diabetes will eventually need insulin to maintain blood glucose control. Most individuals are reluctant to inject themselves and try to avoid it if at all possible.

No.	Barriers	Suggested solutions
5.8.1	Poor understanding of diabetes & its complications, and role of insulin	• Provide comprehensive education.<br>• Explain reduction of risk for complications with better glycaemic control.<br>• Explain the role of insulin in glucose regulation.
5.8.2	Seeing insulin therapy as treatment failure	• Explain the decrease in insulin production which occurs with ageing, so that most people with diabetes will eventually need insulin to maintain glucose control.
5.8.3	Fear of needle	• Provide reassurance that today's needles are much smaller and are coated with silicon, allowing them to slide in more easily. In fact, most people say that it is almost painless and less uncomfortable than a finger stick to monitor blood glucose level.<br>• Use trial injection.
5.8.4	Fear of side effects of insulin i.e. hypoglycaemia and weight gain	• Provide education on how to prevent, recognize and treat hypoglycaemia.<br>• Refer to dietitian before starting insulin.
5.8.5	Lifelong medications / change in lifestyle	• Provide reassurance that with good glucose control achieved after starting insulin, many people have more energy and feel better.

5.9 Practical Guide to Insulin Injection

(Australian Diabetes Educators Association, 2011; American Association of Diabetes Educators, 2014; Siminerio et al, 2011)

5.9.1 Step-by-step Injection Technique

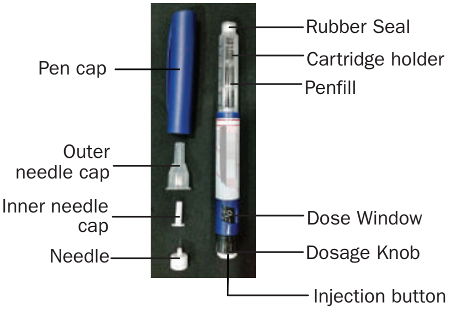

1. Know the parts of the insulin pen and needle.

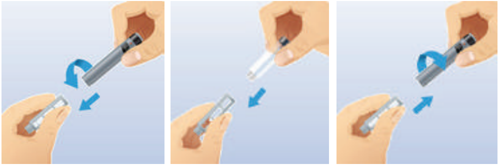

(Image source: Becton Dickinson)

2. Insert the penfill into the cartridge holder (if needed).

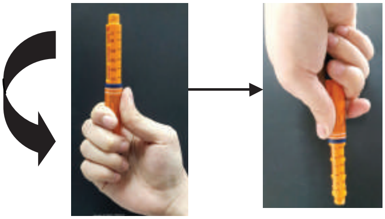

3. Move the penfill up and down gently 10 times until solution becomes milky white. If a cold insulin is used, roll the cartridge or pen in between the palms x 10 times before moving the pen up and down (This step is only needed for cloudy insulin).

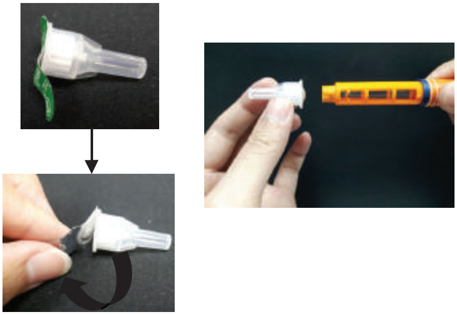

4. Remove the protective tab from a new disposable needle. Attach to the insulin pen.

   Remarks: Use needle **ONLY ONCE**. Reusing needles can bend and dull the tip and increase pain, bleeding and bruises.

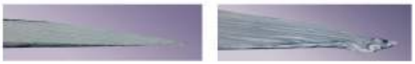

New needle | Blunt needle

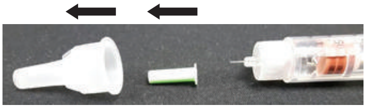

Keep Discard

5. Pull off the outer and inner needle cap. Keep the big needle cap for later.

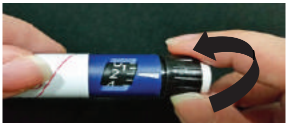

6. Turn the dose selector to select 2 units.

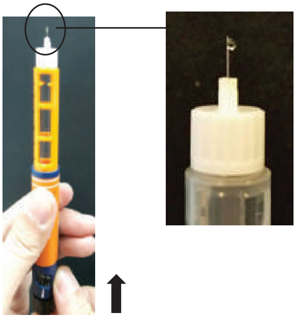

7. Hold the insulin pen with the needle pointing upwards and tap the cartridge gently with a finger a few times.

   Then press the push-button all the way until the dose selector returns to ‘0’. A drop of insulin should appear at the needle tip.

   Remarks: If a drop of insulin still does not appear, the pen is defective, and a new one must be used.

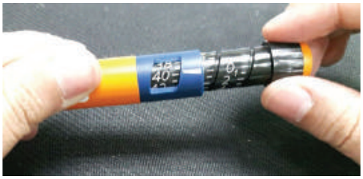

8. Turn the dose selector to select the number of units needed to inject.

   Remarks: Be careful not to push the push-button when turning the dose selector.

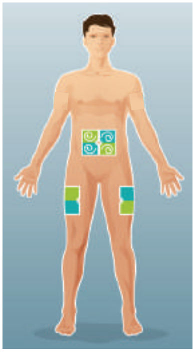

(Image source: Becton Dickinson)

9. Choose the injection site. Main areas for insulin injection are the abdomen and thighs. The abdomen is generally recommended. It is easy to reach, and insulin absorption from the abdomen is more consistent.

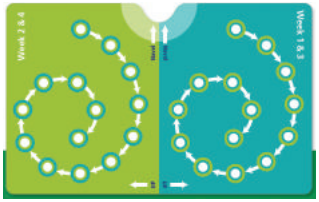

(Image source: Becton Dickinson)

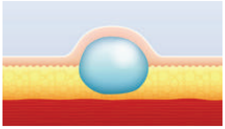

(Image source: Becton Dickinson)

10. MUST inject insulin in different spots within an area. Minimum at least 1 cm distance between two injections.

    Remarks: Injecting insulin at the same spot causes hard lumps and fat deposits, called lipohypertrophy. Injecting into lipohypertrophic area delays insulin absorption (See picture).

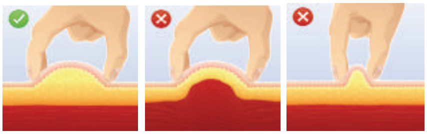

(Image source: Becton Dickinson)

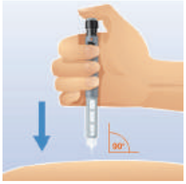

(Image source: Becton Dickinson)

11. Choose the correct needle size: 4mm, 5mm and 6mm needles are suitable for all people with diabetes regardless of BMI. 4 mm needles do not require pinching. 5 mm and 6mm may require pinching in very slim adults (See picture).

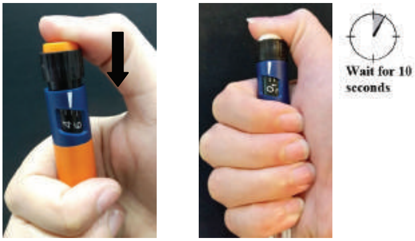

12. Once the injecting area is chosen, inject the dose by pressing the push-button all the way in until ‘0’. Keep the push-button fully depressed and the needle must remain under the skin for at least 10 seconds or count 1-10 to ensure that the full dose has been injected. Pull out pen.

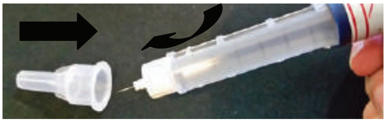

13. Lead the needle tip into the big outer needle cap and cover. Unscrew and dispose the needle into a puncture proofed container (e.g. Milo tin).

    Remarks: Pen needles should be removed after each use to prevent air from entering the cartridge and to prevent insulin from leaking out.

5.9.2 Injection Problems and Solutions

Injection Problems	Solution
Painful injection	• Review injection technique.<br>• Try injecting at a 45 degree to prevent injecting into the muscle.<br>• Inject quickly.<br>• Check that needle is not bent.<br>• Inject insulin when it is at room temperature. Cold insulin hurts.<br>• Try injecting in different site.<br>• Do not use needles more than once. Reusing needles can bend and dull the tip and causes pain.<br>• Keep the muscle at injection area relaxed.<br>• Larger doses hurt more. May benefit from more frequent injection with smaller amount. Check with doctor.
Bleeding at injection site	• Do not rub the injection site.<br>• Apply light pressure with finger to prevent bruising.<br>• If bruising, avoid that injection site again until the bruise resolves.<br>• Frequent bleeding may indicate poor technique or another medical problem. Inform healthcare provider and/or diabetes educator.
Insulin is dripping from the needle after injection	• Wait at least 10 seconds after injecting before removing the needle.<br>• Do not carry a pen with the needle attached.<br>• This causes air to enter the cartridge, thus slowing the time to get insulin dose.
Insulin leaking form injection site	• Count to 10 after the plunger is fully depressed before removing the needle from the skin. This allows enough time for the injected medication to spread out through the tissue planes and/or to cause the tissue to expand and stretch.<br>• A small amount of skin leakage (little pearl of liquid at injection site) can be ignored. It is almost always clinically insignificant.
The injection device is clogged	• Small amount of insulin may be caught in the needle from a previous use. Never reuse needles.<br>• There may be a clump in the insulin; if using cloudy insulin, be sure to properly mix insulin before injection.

5.9.3 Insulin Storage

Unused insulin penfills should be stored at 2 °C – 8 °C in the refrigerator.

Insulin penfills in use should not be kept in the refrigerator. Insulin in use generally lasts for one month at room temperature (< 30 °C).

Do NOT

- Freeze, expose to excessive heat and direct sunlight.
- Use expired insulin.
- Use dry ice to transport or store insulin.
- Keep insulin in unventilated places or vehicles parked under the sun.

Do

- Keep insulin in a clean place.
- Move insulin from time to time to avoid the cloudy insulin from caking.
- Transport insulin to desired destination as fast as possible and follow the temperature guidelines.
- Inspect insulin for unusual changes (frosting, clumping or change in colour).

When Travelling

- Keep insulin in hand-carried luggage.
- Always carry additional supplies.
- Keep insulin in its original packing.
- Do not keep in the car glove compartment.
- Do not use dry ice.

Discard insulin if

- It has been frozen and thawed.
- Colour changes.
- Clumps, flakes or granular deposits present.
- Expired.
- Contaminated.
- Cloudy insulin does not get uniformly cloudy despite mixing.

5.10 Pharmacokinetic Profile of Insulins

Insulin Preparation Brand (Generic) Name	Onset of Action	Peak Action	Duration of Action	Timing of Insulin Administration
Rapid Acting Analogue<br>- Novorapid (Aspart)<br>- Humalog (Lispro)<br>- Apidra (Glulisine)	0-20 min	1-3 hr	3.5-4.5 hr	5-15 min before or immediately after meals.
Short Acting (Human Regular)<br>- Actrapid<br>- Humulin R<br>- Insuman Rapid	30 min	1-4 hr	6-8 hr	30 minutes before meals.
Intermediate Acting (Human NPH Insulin)<br>- Insulatard<br>- Humulin N<br>- Insuman Basal	1-1.5 Hr	4-12 hr	16-23 hr	Pre-breakfast / Pre-bed
Long Acting Analogue<br>- Glargine (Lantus)<br>- Detemir (Levemir)	1-4 Hr	Peakless (For Glargine)<br>3-9 hr (For Detemir)	17-24 hr	Same time everyday at any time of the day
Premixed Human Insulin (30% regular insulin + 70% NPH)<br>- Mixtard<br>- Humulin 30/70<br>- Insuman Comb 30	30 min	Dual	Dual	30 minutes before meals.
Premixed Analogue<br>- NovoMix (30% Aspart + 70% aspart protamine)<br>- Humalog Mix 25 (25% lispro + 75% lispro protamine)<br>- Humalog Mix 50 (50% lispro + 50% lispro protamine)	0-20 min	Dual	Dual	5 to 15 minutes before or immediately after meals.

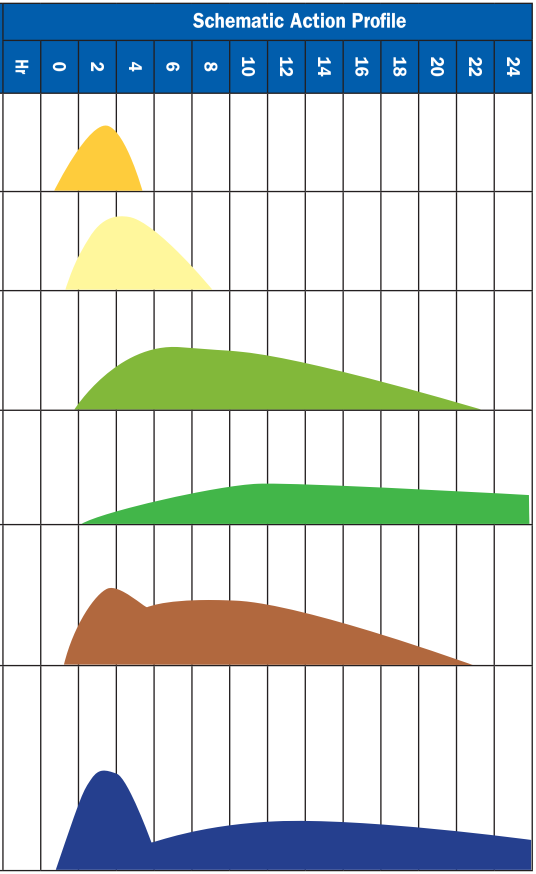

5.11 Commonly used Antihypertensive Medication in Diabetes

Pharmacological treatment is usually started in people with diabetes when BP is persistently > 140 mmHg systolic and / or > 90 mmHg diastolic. Target BP should be aimed at < 135 / 75 mmHg (Ministry of Health Malaysia, 2015) generally and < 130 / 80 mmHg in younger people and those with proteinuria of ≥ 1 g / 24 hours (Ministry of Health Malaysia, 2013). In the presence of microalbuminuria or overt proteinuria, ACEI or ARB should be initiated even if the BP is not elevated.

Class	Starting Dose	Max Dose/ day	Common side effects
**Diuretics**<br>Hydrochlorothiazide<br>Chlorthalidone<br>Amiloride / hydrochlorothiazide (5 mg / 50 mg)<br>Indapamide SR<br>Indapamide	 12.5 mg OD<br>50 mg OD<br>1 tablet OD<br><br><br>1.5 mg OD<br>2.5 mg OD	 50 mg OD<br>200 mg OD<br>2 tablet OD<br><br><br>1.5 mg OD<br>2.5 mg OD	Hypokalaemia, hyponatraemia, hypomagnesaemia, raised serum cholesterol, impaired glucose tolerance, hyperuricaemia and erectile dysfunction.
**β - blockers**<br>Atenolol<br>Bisoprolol<br>Metoprolol<br>Propranolol<br>Carvedilol	 50 mg OD<br>5 mg OD<br>50 mg BD<br>40 mg BD<br>6.25 mg BD	 100 mg OD<br>10 mg OD<br>200 mg BD<br>320 mg BD<br>25 mg BD	Glucose intolerance, bronchospasm, dyslipidaemia, masking of hypoglycaemia, cold extremities, Raynaud's phenomenon, fatigue, nightmares, hallucinations and erectile dysfunction.
**CCBs**<br>(i) Dihydropridines<br>Amlodipine<br>Felodipine<br>Nifedipine LA<br><br>(ii) Non-dihydropridines<br>Diltiazem<br>Diltiazem SR	 <br>5 mg OD<br>5 mg OD<br>30 mg OD<br><br><br>30 mg TDS<br>100 mg OD	 <br>10 mg OD<br>10 mg OD<br>120 mg OD<br><br><br>120 mg TDS<br>200 mg OD	i) Headache, sweating, swelling of ankles, palpitations and flushing.<br><br>ii) Bradycardia, negative inotropic and chronotropic effects can worsen heart failure and cause cardiac arrhythmias.

Class	Starting Dose	Max Dose/ day	Common side effects
**ACEIs**			Dry cough, hyperkalaemia, hypotension, angioedema, deterioration of renal function in those with bilateral reno-vascular disease.
Captopril	25 mg BD	50 mg TDS	
Enalapril	2.5 mg OD	20 mg BD	
Lisinopril	5 mg OD	80 mg OD	
Perindopril	2 mg OD	8 mg OD	
Ramipril	2.5 mg OD	10 mg OD	
**ARBs**			Very similar to those of ACEI except for lower incidence of cough.
Candesartan	8 mg OD	16 mg OD	
Irbesartan	150 mg OD	300 mg OD	
Losartan	50 mg OD	100 mg OD	
Telmisartan	20 mg OD	80 mg OD	
Valsartan	80 mg OD	160 mg OD	
Olmesartan	20 mg OD	40 mg OD	
**Peripheral α – blockers**			Orthostatic hypotension, dizziness, headache and drowsiness, occasionally blood dyscrasias and liver dysfunction.
Prazosin	0.5 mg ON	20 mg in divided doses	
Terazosin	1 mg ON	5 mg OD	

5.12 Dyslipidaemia Therapy in Diabetes

Lipid abnormalities are common in people with diabetes, and contribute to increase in risk of cardiovascular disease. Selection of medication is based on the lipid goal (Ministry of Health Malaysia, 2015).

Lipid Goal	Recommended Drug	Common Side Effects
Lower LDL Cholesterol	Statins, Ezetimibe<br><br>E.g. of statins – Lovastatin, Pravastatin, Simvastatin, Fluvastatin, Atorvastatin, Rosuvastatin	Statins: Myopathy and increased liver enzymes<br><br>Ezetimibe: Headache, abdominal pain and diarrhoea
Increase HDL Cholesterol	Fibrate or Nicotinic Acid<br><br>E.g. of fibrates – Gemfibrozil, Fenofibrate, Ciprofibrate	Fibrate: Dyspepsia, gallstones and myopathy<br><br>Nicotinic Acid: Flushing, hyperglycaemia, hyperuricaemia, upper GIT distress and hepatotoxicity
Lower TG	Fibrates	As above
Treat Combined Hyperlipidaemia	Statins	As above

5.13 Antiplatelet Therapy in Diabetes

Although the benefit of aspirin in secondary CV prevention is well established, that for primary prevention remains controversial. For the primary prevention of cardiovascular disease in people with diabetes at increased cardiovascular risk (10-year risk >10 percent), low dose of aspirin (75 to 150 mg OD) is recommended (Hennekens, 2014).

5.14 Drugs that May Cause Hyperglycaemia

(Rehman et al, 2001; Nigro and Dang, 2013)

**Antihypertensive**	
Beta-adrenergic receptor blockers:	Higher risk with non-vasodilating agents (e.g. Propranolol, Atenolol, Metoprolol), vasodilating agents (e.g. Carvedilol, Nebivolol, Labetalol) do not cause hyperglycaemia
Diuretics (thiazides, thiazide-like, loop)	Most literature with thiazide and thiazide-like diuretics
**Lipid-lowering agents**	
Statins:	Greatest risk with Rosuvastatin and least with Pravastatin
Niacin:	At doses > 2 g/day
**Antiretroviral Agents**	
Nucleoside reverse transcriptase inhibitors	
Protease inhibitors	Atazanavir, Darunavir, Fosamprenavir, Indinavir, Nelfinavir, Ritonivir, Saquinavir, Tipranivir
**Calciceurin inhibitors**	
Cyclosporine, Sirolimus, Tacrolimus	
**Glucocorticoids**	
Greatest risk with systemic formulation (parenteral/oral)	
**Second-Generation antipsychotics**	
Greatest risk with Olanzapine and Clozapine	

5.15 Drugs that May Cause Hypoglycaemia

(Vue and Setter, 2011; Nigro and Dang, 2013; Rehman A et al, 2011; Hooper, 2016)

**Antihypertensive**	
ACE inhibitors	
Beta-adrenergic receptor blockers	Mask many autonomic hypoglycaemic symptoms, can delay recovery from hypoglycaemia, may increase peripheral insulin sensitivity, indirectly decrease gluconeogenesis
**Diabetes medications**	
Insulin	Greater risk with NPH insulin vs long-acting insulin analogues, human regular insulin vs rapid acting insulin analogues, regimens using premixed vs basal-bolus regimens, more intensive regimens (multiple daily doses)
Sulphonylureas	Greatest risk with Glibenclamide vs Glimepiride and Gliclazide
Non-sulphonylurea secretagogues	Lower risk vs sulphonylureas due to shorter duration of action
**Antibiotics**	
Fluoroquinolones	Fluoroquinolones have been associated with both hypoglycaemia and hyperglycaemia. The risk of dysglycaemia should be considered when prescribing fluoroquinolones, particularly in diabetic patients.<br><br>Incidence of Quinolone-induced hypoglycaemia varies within the class. It is most commonly reported in the elderly on concomitant insulin or sulphonylurea.
Sulfamethoxazole	
Quinine	

5.16 Assessment

Refer to initial assessment in Section 2.

It is crucial for people with diabetes to understand their own medication. A diabetes educator may use the following to check understanding of each medication:

a) Indication for the medication

b) Dosage & frequency

c) Time of administration

d) Mode of action

e) Side effects

Adherence to medication may be assessed using Morisky Medication Adherence Scale (Morisky et al, 1986).

5.17 Goal Setting

A diabetes educator must ensure that the people with diabetes are aware of glycaemic, BP and lipid profile targets and are able to comply. These targets should be INDIVIDUALIZED. Below are current recommendations (Ministry of Health Malaysia, 2015):

Parameters		Levels
Glycaemic Control*	Fasting	4.4-6.1 mmol/L
	Non-fasting	4.4-8.0 mmol/L
	HbA1c	≤6.5%
Lipids	Triglycerides	≤1.7 mmol/L
	HDL-cholesterol	≥1.1 mmol/L
	LDL-cholesterol	≤2.6 mmol/L#
Blood Pressure		≤135/75 mmHg
Exercise		150 minutes/week
Body weight		If overweight or obese, aim for 5-10% weight loss in 6 months

* Glycaemic target should be individualised to minimise risk of hypoglycaemia.

# In Individuals with overt CVD, LDL cholesterol target is <1.8 mmol/L.

5.18 Planning

Diabetes treatment should be planned according to current evidence based guidelines in collaboration with individuals with diabetes.

5.19 Implementation

Diabetes educator can provide feedback to doctor regarding problems with adherence to medication.

Educators are encouraged to create a chart of all the medications in their institution to aid identification. The medication chart can be photos or actual pills in small plastic wrappers stapled to a stiff cardboard sheet.

5.20 Evaluation and Monitoring

5.20.1 Insulin Administration Checklist

Use the checklist below to evaluate injection practices and determine areas of educational need.

Name:

SRN:

Sex: F / M

Age:

No	Checklist	Date Of Education	Date Of Reinforce
1.	Assessment of learning needs and ability to participate in self injection		
2.	Information on onset, action and duration of insulin, doses and timing.		
3.	Injection site<br>• Selection of injection site<br>• Evidence of<br>☐ Lipohypertrophy<br>☐ Lipoatrophy<br>☐ Bruising<br>☐ Infection<br>• Describe injection rotation pattern		
4.	Injection techniques<br>• Hand wash<br>• Assemble equipment appropriately<br>• Check insulin for discolouration, formation of clumps etc. Discard if these occur.<br>• Rocking up and down 10 times each to ensure uniformity (only for cloudy insulin)<br>• Attachment of needle<br>• Pre-injection priming<br>• Dosing accuracy<br>• Site pinch-up (depends on needle depth)<br>• Complete injection<br>• Needle withdrawal with waiting of 6 seconds / 10 counts		
5.	Disposal of needle		
6.	Needle reuse		
7.	Storage of insulin		
8.	Assess knowledge of hypoglycaemia symptoms and management.		
9.	Education carried out by (Name & Sign)		

5.20.2 Oral Anti-Diabetic (OAD) Agent Checklist

1.	**Compliance to OAD regime**	**Please tick (√) if applicable**
	• Indication	
	• Dose of OAD	
	• Frequency of OAD	
	• Timing of OAD<br>(e.g. Gliclazide MR: before the first meal of day, Metformin XR: after dinner)	
	• Method of administration<br>(e.g. Acarbose: with meals, Sulphonylurea: before meals, Metformin: after meals)	
2.	**Tolerance to OAD regime**	
	• **Acarbose:** diarrhoea, abdominal pain, flatulence<br>• **Metformin:** Anorexia, nausea, vomiting, diarrhoea<br>• **DPP-IV inhibitor:** nausea, diarrhea, headache, flu-like symptoms, severe joint pain, risk of pancreatitis (symptoms: nausea, vomiting, anorexia, persistent severe abdominal pain, sometimes radiating to the back)<br>• **SLG2 inhibitor:** hypoglycaemia (when used with sulphonylureas/ insulin), polyuria, genital infection, urinary tract infection, risk of bone fracture & decrease bone mineral density, risk of ketoacidosis (symptoms: nausea, vomiting, abdominal pain, tiredness and trouble breathing)	
3.	**Glucose control**	
	• Pre-breakfast blood glucose	
	• Post-prandial blood glucose	
	• HbA1c	

5.21 References

1. American Association of Diabetes Educators (2014) Strategies for insulin injection therapy in diabetes self-management. http://www.diabeteseducator.org. Accessed on 16 December 2014.

2. American Diabetes Association (2015) Standards of medical care in diabetes. *Diabetes Care* Vol. 38 (Suppl 1),537 - 552.

3. Australian Diabetes Educators Association (2011) ADEA Clinical recommendations subcutaneous injection technique for insulin and glucagon like peptide. *ADEA*, 11-12.

4. Hennekens, C.H. (2014) Overview of primary prevention of coronary heart disease and stroke. www.uptodate.com. Accessed on 16 June 2014.

5. Hooper, D.C. (2016) Fluoroquinolones. www.uptodate.com. Accessed on 09 April 2016.

6. Ministry of Health, Malaysia (2015) *Clinical Practice Guidelines: Management of Type 2 Diabetes Mellitus (5th edition)*.

7. Ministry of Health, Malaysia (2013) *Clinical Practice Guidelines: Management of Hypertension (4th Edition)*. 24-26, 47-54.

8. Morisky, D.E., Green, L.W. and Levine, D.M. (1986) Concurrent and predictive validity of a self-reported measure of medication adherence. *Medical Care* Vol. 24, 67-74.

9. Nigro, S. and Dang, D.K. (2013) Drug-induced hyperglycemia and hypoglycemia, nonprescription medications, and complementary and alternative medicine for diabetes care. *Drug Topics*, 48-57.

10. Rehman, A., Setter, S.M., and Vue, M.H. (2011) Drug-induced glucose alterations part 2: drug-Induced hyperglycemia. *Diabetes Spectrum* Vol. 24, 234-238.

11. Siminerio, L., Kulkarni, K., Meece, J. et al (2011) Strategies for insulin injection therapy in diabetes self-management. *Diabetes Education* Vol. 37, 1-10.

12. Vue, M.H. and Setter, S.M. (2011) Drug-induced glucose alteration part 1: drug-induced hypoglycemia. *Diabetes Spectrum* Vol. 24, 171-177.

SECTION 6

SELF-MONITORING

No		Content	Page
6.1		Introduction to Self-monitoring	73
	6.1.1	Definition of Self-monitoring	73
6.2		Self-monitoring Blood Glucose	74
	6.2.1	Introduction	74
	6.2.2.	Definition	74
	6.2.3	Objectives	74
	6.2.4	General Recommendations	74
	6.2.5	Assessment	77
	6.2.6	Goal Setting	79
	6.2.7	Planning	79
	6.2.8	Implementation	81
	6.2.9	Interpretation and Action	82
	6.2.10	Evaluation	83
	6.2.11	Trouble Shooting for Blood Glucose Reading	84
	6.2.12	Care of Equipment	84
	6.2.13	Checklist for Healthcare Professional	84
6.3		Blood Pressure Monitoring	85
	6.3.1	Introduction	85
	6.3.2	Recommendations for BP Measuring Technique	85
	6.3.3	General Recommendations	86
	6.3.4	Evaluation	86
6.4		Weight Monitoring	87
	6.4.1	Introduction	87
	6.4.2	Definition	87
	6.4.3	Implementation	88
	6.4.4	Evaluation	88
6.5		References	89
6.6		Appendices	90

Self-Monitoring for Type 2 Diabetes Mellitus

6.1 Introduction to Self-monitoring

Type 2 Diabetes Mellitus is a chronic condition. People with diabetes are advised to perform self-monitoring of blood glucose, blood pressure and weight in order to manage his/her condition and to reduce or prevent acute and long term complications. This chapter aims to provide guidelines on knowledge and skills in self-management for people with diabetes. It includes self-monitoring of blood glucose, home blood pressure monitoring and body weight.

6.1.1 Definition of Self-monitoring

Home self-monitoring is defined as the ability for a person with a chronic disease to manage his or her own illness. It should not be considered as a stand-alone intervention but used in conjunction with appropriate therapy such as healthy lifestyle choices and informed decisions with regards to current treatment options. Home self-monitoring should be in partnership with a team of health care providers for integrated self-care as it requires lifelong choices, skills and strategies for optimal management in the long term (Health Service Executive, 2008).

6.2 Self-Monitoring of Blood Glucose

6.2.1 Introduction

Self-monitoring of blood glucose (SMBG) is widely used in clinical practice and is accepted as part of diabetes management. It can be used to detect or confirm hypoglycaemia and hyperglycaemia. It helps people with diabetes to better understand the impact of lifestyle on glycaemic control thus encouraging positive changes to enhance well-being and quality of life. Optimization of glycaemic control is important in diabetes management to prevent and / or reduce risk of acute and chronic complications

(American Diabetes Association, 2015; Bergenstal and Gavin, 2005)

6.2.2 Definition

Self-monitoring of blood glucose is the use of a glucose meter to enable a person with diabetes to recognize glycaemic variations. The result provides immediate feedback regarding the impact of his or her lifestyle behaviour and if pharmacological interventions are required.

(American Association of Diabetes Educators, 2014)

6.2.3 Objectives

- To improve diabetes self-management
- To provide reassurance as well as empower people with diabetes to take control of diabetes self-management
- To optimize or intensify diabetes management either with insulin treatment or oral glucose-lowering medications or lifestyle adaptations
- To provide better assessment of changes of glycaemic levels resulting from medication and lifestyle changes

6.2.4 General Recommendations

The following factors should be considered when facilitating people with diabetes to determine monitoring frequency and timing:

- Medication regimen
- Level of diabetes control with regards to activity, food and work
- Financial limitations
- Comorbid conditions, e.g. Chronic Kidney Disease, Coronary Artery Disease etc
- Ability to interpret the results and take appropriate action

SMBG recommendations for different therapy regimes

i) SMBG regime for Insulin Therapy

Table 1: Basal / Basal Bolus Regime

	Breakfast		Lunch		Dinner		Bedtime
	Pre	Post	Pre	Post	Pre	Post	Pre
Basal only	x						
Basal bolus (Short-acting)	x		x		x		x
Basal bolus (Rapid-acting)	x	x		x		x	

Table 2: Premixed Regime

	Breakfast		Lunch		Dinner		Bedtime
	Pre	Post	Pre	Post	Pre	Post	Pre
Pre-mixed Human BD	x		x		x		x
Pre-mixed Analogues BD	x	x			x	x	
Pre-mixed Analogues TDS	x	x	x	x	x	x	

(Adapted from Practical Guideline to Insulin Therapy in Type 2 Diabetes Ministry of Health Malaysia, 2010)

ii) SMBG regime for oral anti-diabetic medication

Table 3: Oral Anti-diabetic SMBG Regime

**Mode of treatment**	Breakfast		Lunch		Dinner	
	Pre	Post	Pre	Post	Pre	Post / Pre-bed
Oral Anti-Diabetic Agent	x	x		x		x

(Adapted from Clinical Practice Guideline Management of Type 2 Diabetes Mellitus Ministry of Health Malaysia, 2015)

Table 4: Oral Anti-Diabetic Agent SMBG Regime

Medication	Fasting	Post-prandial
Biguanides	x	x
Insulin secretagogues<br>Sulfonylureas, Meglitinides	x	x
Sodium Glucose Co-Transporter 2 inhibitors<br>Dapaglifozin, Canaglifozin	x	x
Alpha-Glucosidase inhibitors<br>Acarbose		x
Incretin Based Therapies<br>DPP-4 inhibitors, GLP-1 receptor agonist		x
Amylin analogs<br>Pramlintide		x

(Adapted from Austin and D'Hondt, 2014)

The above table also serves as a guideline in the following situations:

- People with diabetes who are new to oral hypoglycaemic therapy especially on the drug group of Sulphonylureas.
- People with diabetes who are undergoing dose adjustment.
- If blood glucose readings are stable and on target, SMBG can be done up to 2-4 readings per week at varying times to identify asymptomatic hyperglycaemia and assess glucose excursion due to lifestyle changes from the routine.

(Ministry of Health Malaysia, 2015)

iii) SMBG regime on Diet Alone

- SMBG should be done on an intermittent basis, to assess glucose excursions due to lifestyle changes.

Table 5: Diet Alone SMBG regime

	Breakfast		Lunch		Dinner	
Mode of treatment	Pre	Post	Pre	Post	Pre	Post / pre-bed
Diet only	x	x		x		x

(Adapted from Clinical Practice Guideline Management of Type 2 Diabetes Mellitus Ministry of Health Malaysia, 2015)

iv) SMBG regime for special situations:

More frequent SMBG is required for the following:

- People with diabetes who are at increased risk of developing hypoglycaemia.
- People with diabetes who experience acute illness, e.g. sick days.
- People with diabetes on insulin before long distance driving.
- People with diabetes who participate in vigorous exercise.

(American Diabetes Association, 2014)

- People with diabetes undergoing fasting, e.g. during Ramadan; SMBG should be done:
- Pre Sahur (pre-dawn meal) and 2 hours post Sahur
- Pre Iftar (Sunset) and 2 hours post Iftar

(Ministry of Health Malaysia, 2010)

6.2.5 Assessment

1. Refer to initial assessment Section 2.
2. In addition to the above, additional assessment has to be considered as follows:
   - Financial history
     To assess the ability to afford the items for performing SMBG
   - Health literacy
     To assess the ability to use, understand instructions and interpret the blood glucose result accurately e.g. "What will you do if your blood glucose reading is 4.0 mmol/L."
   - Barriers to SMBG
     If any and resolve it using the proposed approach as in table 6

Table 6: Approaches to Overcome Barriers to SMBG

Barrier	Solution
Cost	Ensure correct testing technique to minimize wastage of test strip. Facilitate people with diabetes on structured testing regimen to make every test result useful for intervention if required.<br>Explore programs offered by meter companies and social services agencies which offer financial aid.
Pain perception	Ensure correct technique by identifying sites of pricking- at the side of the fingertips and not at finger pads (more nerve endings at the tips of finger cause more pain).<br>Use finer lancet size.<br>Check the numbering/dots of the lancing device to ensure the depth is appropriate for pricking.<br>Choose meters and test strips requiring small sample size.
Language/Instructions	Involve caregivers for people with diabetes who are unable to understand instructions.<br>Use pictures and illustrations for better understanding.
See no value in monitoring	Acknowledge effort taken to perform SBMG by:<br>• saying "Thank you"<br>• help in interpreting result<br>• help in problem solving<br>• giving reassurance
Forgetting/ inconvenience	Questions to encourage<br>• 'How important is SMBG to you, why?<br>• What is your greatest concern or frustration about SMBG?<br>• What is the hardest part about monitoring?<br>• What do you need to help you monitor your blood glucose?
Health literacy/ numeracy	• Include caregiver in training.<br>• Provide information in simple, practical and usable format.<br>• Introduce one concept at a time, ensure it is understood before introducing the second subject.<br>• Demonstrate and illustrate.<br>• Provide simple written information or pictorial information.

(American Association of Diabetes Educators, 2014)

6.2.6 Goal Setting

- This is individualized according to assessment findings and Malaysian CPG 2015 guidelines (Table 7).
- SMART: It should be **S**pecific, **M**easurable, **A**chievable and **A**ffordable, **R**eliable and **T**ime specific.
- Make informed choices to ensure that the person is able to achieve the goal.

Table 7: Guidelines for Glycaemic Control in Type 2 Diabetes Mellitus

	Malaysian CPG (2015)	IDF	ADA	AACE
Fasting/Pre-prandial glucose (mmol / L)	4.4 - 6.1	< 6.0	3.7 - 7.2	< 6.0
Non-fasting / 2-h postprandial glucose (mmol / L)	4.4 - 8.0	< 7.8	< 10.0	< 7.8
HbA1c (%)	≤ 6.5	≤ 6.5	< 7.0	≤ 6.5

(Adapted from Clinical Practice Guideline Management of Type 2 Diabetes Mellitus Ministry of Health Malaysia, 2015)

6.2.7 Planning

- Discuss the need for SMBG according to goal setting. Discuss and get mutual agreement between the person with diabetes and health care team on SMBG regime.
- Encourage SMBG as part of diabetes self-management using sample questioning in Table 8.
- Frequency of blood glucose monitoring depends on the glycaemic status and goal, mode of treatment, concomitant underlying condition as well as psychosocial factors (Refer to Appendix 1).
- Educational tools: Logbook, video clip, brochure, instructional guideline.
- Recommended glucometer should meet **ISO 15197 minimum accuracy acceptable criteria (MACC) by medical requirement. (Information can be obtained in the glucometer instruction booklet).**

Table 8: Sample Questioning to Enhance the Process of SMBG

No	Instead of	Consider
1.	What is the most difficult part of blood glucose monitoring?	What is the easiest part of blood glucose monitoring for you?<br>What would make blood glucose monitoring easier for you?
2.	Why did you forget to test in the morning?	What helps you remember to test?
3.	Your numbers are running high in the morning; what do you think you are doing wrong?	Look how great your numbers are on these days (or times). Let's talk about what you do then.
4.	Why aren't you checking your blood glucose?	How important is blood glucose monitoring to you?
5.	Why are your post-dinner blood glucose levels always high?	Tell me about your dinner meal.<br>Tell me about what happens the hour or two before dinner.
6.	Why are checking only fasting blood glucose?	Tell me what your fasting blood glucose is telling you.
7.	Why aren't you checking your blood glucose at least twice per day?	What part of your day would you most like to know something about your blood glucose results?<br>How often do you think you would want to look at(check) your blood glucose results in a week?
8.	Do you think checking your blood glucose 3 times a day is realistic?	On a scale of 1-10, how confident are you that you can check your blood glucose 3 times a day every day of the week? (If not confident, ask if he/she would like to change the goal to one in which she or he felt confident.)

(Adapted from the American Association of Diabetes Educators, 2014)

6.2.8 Implementation

Follow the glucometer manufacturer's guidelines on method / technique of using the glucometer.

Demonstrate the proper technique by following the manual provided (Table 9) (Ed Bryant, 2015).

Table 9: Steps in Performing Capillary Blood Glucose Test

1.	Wash hands and ensure the site is completely dry before obtaining blood sample.	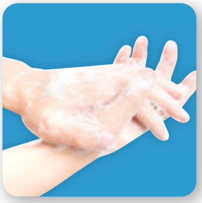<br>Wash hands, rinse and dry
2.	Install lancet.	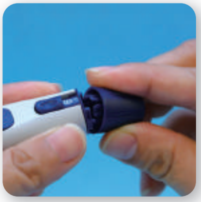 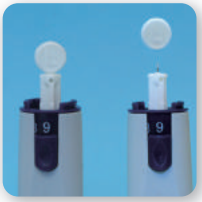 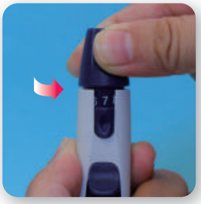<br>Remove the cap (twist it) and insert a lancet<br>Adjust the depth setting
3.	Insert test strip.	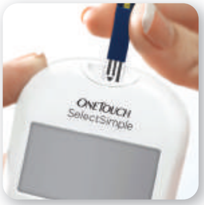
4.	Prick a spot on the side of your finger tip.	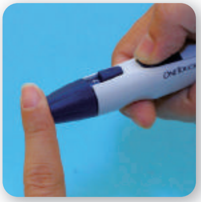

5.	Apply blood on tip of test strip.	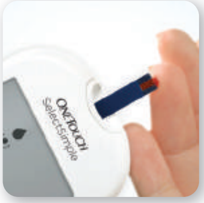
6.	In seconds, the blood glucose result will appear automatically. Remove and dispose of the test strip.	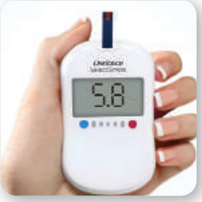

(Image source: Johnson and Johnson)

- Record results: Date, time, level of blood glucose reading in mmol/L.
- Involve family members during the demonstration to ensure the person will be assisted by family members in case of difficulty.
- Provide individualized blood glucose target in collaboration with health care team, based on age, duration of diabetes, presence of comorbidities, life expectancy and risk of hypoglycaemia.

6.2.9 Interpretation and Action

People with diabetes should be educated on SMBG data with regards to adjusting food intake, exercise, or pharmacological therapy in order to achieve individualized goals and targets.

General guidelines:

- Blood glucose < 4 mmol/L indicates hypoglycaemia
- Blood glucose > 15 mmol/L indicates hyperglycaemia

Table 10: SMBG Interpretation and Intervention

SMBG Timing	Information provided
Fasting glucose	Assess overnight effect of medications.<br>Residual beta-cell function.<br>If fasting is higher than bedtime, possible nocturnal hypoglycaemia or dawn effect.
Pre-prandial	Efficacy of previous basal insulin or intermittent insulin.<br>Assess previous meal CHO intake.
Post-prandial glucose	Assess adequacy of pre-meal medications with the meal taken.<br>If no pre-meal medications, assess the effect of CHO intake.
Bed time	Assess the effect of evening meals/supper and pre-dinner insulin therapy.
Random	Determine that the symptoms experienced are due to blood glucose fluctuation.

(Adapted from Clinical Practice Guideline Management of Type 2 Diabetes Mellitus Ministry of Health Malaysia, 2015)

6.2.10 Evaluation

1.	Assessment of technique of performing blood glucose testing<br>• Ask the person with diabetes to bring glucometer to reassess his/her technique of performing blood glucose testing.
2.	Review and discuss blood glucose results in logbook.
3.	Assess ability to interpret blood glucose results and take appropriate actions if results are not on target.
4.	Discuss any challenges encountered.
5.	For unexpected high and low SMBG readings, healthcare providers may use Table 11 to identify problems.

Table 11: Trouble Shooting for Unexpected Blood Glucose Readings

False Highs	False Lows	False Highs / Lows
• Sample site contaminated (Fruit, juice, foods that contain sugar)<br>• Dehydration<br>• Anaemia	• Inadequate blood sample<br>• Medical shock<br>• Milking finger too vigorously<br>• Polycythemia	• Finger wet due to alcohol or water<br>• Expired test strip<br>• Test strip stored at extreme temperature<br>• Test strip container compromised or left open<br>• Alternate site testing (other than finger sites) at times of blood glucose variability.

(American Association of Diabetes Educators, 2014)

6.2.11 Care of Equipment

- Blood Glucose Meter:
  - ➤ Clean the meter according to glucometer instruction booklet.
- Test strip:
  - ➤ Storage of test strips should be as recommended by individual glucometer company.
  - ➤ Do not expose to extreme hot or cold temperature as well as humidity.
  - ➤ Strips should be stored in package provided.
  - ➤ Dispose used lancets and test strips appropriately to avoid cross contamination.

6.2.12 Checklist for Healthcare Professional

Refer to Appendix 2

6.3 Blood Pressure Monitoring

6.3.1 Introduction

Hypertension is common among people with diabetes leading to additional increase in risk of cardiovascular disease. Hence, home blood pressure monitoring is essential for people with diabetes. According to the ADA, blood pressure should be measured routinely to a goal of <140/80 mmHg.

6.3.2 Recommendations for Blood Pressure Measuring Technique

- Blood pressure should be measured correctly using correct technique either by mercury sphygmomanometer or aneroid manometer or a validated electronic device.
- Size of Blood Pressure Cuff depends on upper arm circumference as in table 12.
- Seated quietly for at least 5 minutes before blood pressure is taken.
- Smoking, caffeine intake and physical exercise are avoided for at least 30 minutes prior to measurement.
- The person should be seated in a quiet room, with back supported, arm supported (for example, resting on the table).
- The arm is supported at heart level.
- Outer garments are removed; avoid rolling sleeves if this causes constriction.
- Legs should be uncrossed. The person should be relaxed.
- The correct cuff bladder is placed at heart level.
- Talking is avoided during the reading.

Table 12: Measurement of Blood Pressure Cuff

Arm circumference	Blood pressure cuff size
15 - 22.5 cm (7 - 9 inches)	Small adult cuff
22.5 - 32.5 cm (9 - 13 inches)	Standard adult cuff
32.5 - 42.5 cm (13 - 17 inches)	Large adult cuff

6.3.3 General Recommendations

- Monitor blood pressure once or twice per week (for long term monitoring of hypertension).
- A minimum measurement for 3 days per week or ideally daily should be performed.
- Should be done at about same time once in the morning and evening.
- Morning (before drug intake if treated) and evening (before meal) readings should be taken with two measurements per occasion (1-2 minutes apart).
- The results should be recorded in a specific logbook or stored in device memory.
- People with diabetes are provided with their individualized blood pressure goal.
- Lifestyle modification is encouraged as an integral part in reducing blood pressure.
- People with diabetes need advice on weight loss, regular exercise, restriction of alcohol and reduced salt consumption.

(British Hypertension Society, 2011; National Institute for Health and Care Excellence, 2015)

6.3.4 Evaluation

1.	Assess adherence to blood pressure monitoring frequency according to goal setting.
2.	Assess if the person with diabetes achieves the individualized BP target.
3..	Assess adherence to blood pressure medication.
4.	Assess adherence to lifestyle modification.
5.	Assess regularity of follow-up with health care providers.
6.	Discuss challenges in achieving desired goal and discuss/propose solutions to overcome them.

6.4 Weight Monitoring

6.4.1 Introduction

Many people with diabetes are overweight or obese. Weight loss of 5 - 10% of total weight improves insulin sensitivity and glucose tolerance. In addition it helps to prevent or delay diabetes related complications such as cardiovascular disease. (Wing et al, 2011; Ministry of Health Malaysia, 2004; Colosia et al, 2013).

People with diabetes should be advised on weight management through healthy meal planning and physical activity. It is important to collaborate with a registered dietitian in healthy meal planning. (Refer to section 3 and 4 for Healthy Eating, Physical Activity and Exercise).

6.4.2 Definition

Body mass index (BMI) is an established and widely used measurement of obesity and is defined as BMI = weight (kg) / Height (m^2). Table 13 shows the classification of body weight by BMI.

Table 13: Classification of Weight by Body Mass Index

Classification	BMI (kg / m^2)
Underweight	< 18.5
Normal range	18.5 - 22.9
Overweight	≥ 23
Pre-obese	23.0 - 27.4
Obese I	27.5 - 34.9
Obese II	35.0 - 39.9
Obese III	≥ 40.00

(Adapted from Clinical Practice Guideline on Management of Obesity, 2004)

6.4.3 Implementation

- Measure body weight at the same time once a week wearing light clothing.
- Self-monitoring "tracker"- this is a device to monitor daily physical activity, food intake and weight record.

(Eaki, 2010)

6.4.4 Evaluation

1.	Assess whether the person with diabetes has achieved the individualized weight target.
2.	Assess his or her adherence to healthy lifestyle advice. If target weight not achieved, reassess challenges in his/her lifestyle behaviour and reset a new short term goal.

6.5 References

1. Austin, M.A. and D'Hondt, N. (2014) Monitoring In Mensing, C., Cornell, S. and Halstenson, C. (3rd edition) *The Art & Science of Diabetes Self-Management Desk Reference* (pp 197-246). Chicago: American Association of Diabetes Educators.

2. American Diabetes Association (2015) Standards of Medical Care In Diabetes. *Diabetes Care* Vol. 38 (Suppl.1), S14-S79.

3. Bergenstal, R.M. and Gavin, J.R. Global Consensus Conference on Glucose Monitoring Panel. (2005) The role of self-monitoring of blood glucose in the care of people with diabetes: report of a global consensus conference. *American Journal of Medicine* Vol.118,1S-6S.

4. British Hypertension Society (2011) *Home Blood Pressure Monitoring Protocol.* Available from: http://www.bhsoc.org/files/4414/1088/8031/Protocol.pdf. Accessed on 25 January 2015.

5. Colosia, A.D., Palencia, R. and Khan, S. (2013) Prevalence of hypertension and obesity in patients with type 2 diabetes mellitus in observational studies: a systematic literature review. *Diabetes Metabolic Syndrome and Obesity* Vol.6, 327-338.

6. Eakin, E.G., Reeves, M., Marshall, A.L. et al. (2010) Living Well with Diabetes: a randomized controlled trial of a telephone-delivered intervention for maintenance of weight loss, physical activity and glycemic control in adults with type 2 diabetes. *BioMedCentral Public Health* Vol. 10, 452-466.

7. Ed Bryant. (2015) *Finger Sticking Techniques.* Available from: https://nfb.org/finger-sticking. Accessed on 22 March 2015.

8. Health Service Executive (2008) A Practical Guide To Integrated Type 2 Diabetes Care. Available from: http://www.hse.ie/eng/services/publications/topics/Diabetes/A_Practical_Guide_to_Integrated_Type_II_Diabetes_Care.pdf. Accessed on 22 March 2015.

9. International Diabetes Federation (2009) *Self-Monitoring of Blood Glucose in Non-Insulin Treated Type 2 Diabetes.*

10. Ministry of Health Malaysia (2004) *Clinical Practice Guideline on Management of Obesity.*

11. Malaysian Endocrine and Metabolic Society (2010) *Practical Guide To Insulin Therapy in Type 2 Diabetes Mellitus.*

12. Ministry of Health Malaysia (2015) Clinical Practice Guideline *Management of Type 2 Diabetes Mellitus* (5th Edition).

13. National Institute for Health and Care Excellence (2015) *Managing blood pressure in type 2 diabetes*. United Kingdom. Available from: http//pathways.nice.org.uk/pathways/diabetes. Accessed on 4 March 2015.

14. Wing, R.R, Lang, W., Wadden, T.A. et al. (2011) Benefits of Modest Weight Loss in Improving Cardiovascular Risk Factor in Overweight and Obese Individual with Type 2 Diabetes. *Diabetes Care* Vol. 43,1481-1486.

6.6 Appendices

Appendix 1

List of SMBG Regimes

The following are examples of SMBG regimes from International Diabetes Federation (2009) and Malaysian Clinical Practice Guideline 2015. The IDF recommends different intensity of SMBG regime based on individual needs, goals and psychosocial factors.

1. 7-point Profile

	Pre-breakfast	Post-breakfast	Pre-lunch	Post-lunch	Pre-supper	Post-supper	Bedtime
Monday							
Tuesday	x	x	x	x	x	x	x
Wednesday	x	x	x	x	x	x	x
Thursday	x	x	x	x	x	x	x
Friday							
Saturday							
Sunday							

2. 5-point Profile

	Pre-breakfast	Post-breakfast	Pre-lunch	Post-lunch	Pre-supper	Post-supper	Bedtime
Monday							
Tuesday							
Wednesday	x	x		x	x	x	
Thursday	x	x		x	x	x	
Friday	x	x		x	x	x	
Saturday							
Sunday							

3. Staggered SMBG Regime

	Pre-breakfast	Post-breakfast	Pre-lunch	Post-lunch	Pre-supper	Post-supper	Bedtime
Monday	x	x					
Tuesday			x	x			
Wednesday					x	x	
Thursday	x	x					
Friday			x	x			
Saturday					x	x	
Sunday	x	x					

4. Low-intensity SMBG Regime: Meal-based

	Pre-breakfast	Post-breakfast	Pre-lunch	Post-lunch	Pre-supper	Post-supper	Bedtime
Monday	x	x					
Tuesday							
Wednesday			x	x			
Thursday							
Friday							
Saturday					x	x	
Sunday							

5. Detection / Assessment of Fasting Hyperglycaemia

	Pre-breakfast	Post-breakfast	Pre-lunch	Post-lunch	Pre-supper	Post-supper	Bedtime
Monday							x
Tuesday	x						
Wednesday							x
Thursday	x						
Friday							x
Saturday	x						
Sunday							

6. Detection of Asymptomatic Hypoglycaemia

	Pre-breakfast	Post-breakfast	Pre-lunch	Post-lunch	Pre-supper	Post-supper	Bedtime
Monday			x		x		
Tuesday							
Wednesday			x		x		
Thursday							
Friday			x		x		
Saturday							
Sunday							

Appendix 2

Checklist for Healthcare Provider

Assessment checklist

		Remark / Date
☐	Newly diagnosed	____________________
☐	On insulin treatment	____________________
☐	On Sulphonylurea drugs	____________________
☐	Having acute illness	____________________
☐	Having frequent hypoglycaemic episodes	____________________

Your role in SMBG:

- ☐ Explain the importance of SMBG
- ☐ Technique of using glucose meter
- ☐ Explain individualized blood glucose target
- ☐ Explain the ideal target reading of blood glucose
- ☐ Ask the person with diabetes or care giver to demonstrate use of glucose meter
- ☐ Provide blood glucose logbook
- ☐ Follow up appointment
- ☐ Discussion issue: State:________________________________

SECTION 7

RISK REDUCTION

No		Contents	Page
7.1		Introduction	95
7.2		Cardiovascular Complications	95
	7.2.1	Hypertension	95
	7.2.2	Dyslipidaemia	96
	7.2.3	Smoking Cessation	98
7.3		Diabetic Nephropathy	99
7.4		Diabetic Retinopathy	100
7.5		The Diabetic Foot	101
7.6		Hypoglycaemia	102
7.7		References	104
7.8		Appendices	105

Risk Reduction

7.1 Introduction

People with diabetes are at risk of various complications such as cardiovascular diseases/ events, diabetic nephropathy, diabetic retinopathy, diabetes foot ulcer and hypoglycaemia.

Risk reduction

Risk reduction in people with diabetes for cardiovascular diseases, diabetic nephropathy, diabetic retinopathy, diabetes foot ulcer and hypoglycaemia is based on blood glucose control as well as healthy lifestyle activities. Diabetes educators are advised to work with people with diabetes to reduce the above risks and achieve the goals of self-management of diabetes.

7.2 Cardiovascular Disease/Events

The combination of diabetes, hypertension, dyslipidaemia and smoking markedly increases the risk of cardiovascular disease (CVD), and coronary heart disease. In addition to optimal glycemic control, blood pressure and cholesterol control, tobacco smoking causes harm to almost all body organs resulting in a wide range of disease. Smoking cessation is associated with risk reduction in cardiovascular disease/event and prolonged life expectancy. Routine clinical monitoring using Clinical Monitoring Schedule (Appendix 1, Figure 7.1.) and smoking cessation are recommended to reduce or prevent risk of diabetes chronic complications.

7.2.1 Hypertension

Assessment

- Blood pressure measurement is recommended at every routine clinic visit.
- Measurement in the seated position, with feet on the floor and arm supported at heart level, after 5 minutes of rest.
- Cuff size should be appropriate for the upper arm circumference (refer section 6, Self-monitoring).
- Elevated values are to be confirmed.
- Review self-monitoring of blood pressure records.
- Identify knowledge of blood pressure control:
  - What do you understand about your BP readings and its risk?
  - Are you taking any medications for high blood pressure?
  - When do you usually consume them?
  - How often do you miss your medications?
  - Can you tell me more about your concern on the side effects of medications?
  - How often do you eat out?
  - Do you usually put additional salt/soya sauce in your meals?

Goal Setting

Achieve target blood pressure.

The target blood pressures recommended are as follow:

- Target BP <140/80 mmHg in all people with diabetes
- Target BP <130/80 mmHg is recommended for younger age group and people with diabetes with Ischaemic Heart Disease (IHD)/Cardiovascular Vascular Disease (CVD) /renal impairment.

(Ministry of Health Malaysia, 2013)

Planning

- Planning of care based on assessment and management of hypertension as shown in Appendix 2 and 3, Management of hypertension (Ministry of Health Malaysia, 2013).
- Plan appropriate action with people with diabetes on strategies to improve BP control using Action plan for BP and lipid control (Appendix 4).

Implementation

- Explore pros and cons of blood pressure control and decision for action plan including Diabetes education using Two-way dialogue and Decisional Balance Box (Appendix 5).
- Discuss strategies to achieve BP target (Section 3, Healthy Eating, Section 4, Physical Activity and Exercise and Section 5, Medication).

Evaluation

- Evaluate the blood pressure control by using evaluation check list for hypertension (Appendix 6).

7.2.2 Dyslipidaemia

Assessment

- Lipid profile is recommended at time of diagnosis and at least annually.
- More frequent lipid profile may be needed especially after commencement of treatment.
- Assess knowledge and attitude towards lipid control:
    - What do you understand about your cholesterol results and its risk?
    - Are you taking any medication for reducing cholesterol?
    - When do you usually take your medication?
    - How often do you miss your medication?
    - Are you concerned about the side effects of medication?

Goal Setting

People with diabetes are able to achieve target lipid levels.

The targets of cholesterol level recommended are as follows:

1) Primary target: LDL cholesterol

i) In individuals without overt CVD

a) All patients over the age of 40 years should be treated with a statin regardless of baseline LDL cholesterol levels.

b) The target of LDL cholesterol level is 2.6 mmol / L.

ii) In individuals with overt CVD

a) All patients should be treated with a statin.

b) The target of LDL cholesterol level is 1.8 mmol / L.

2) Secondary target: Non-HDL cholesterol, HDL cholesterol and TG

i) Non-HDL cholesterol < 3.4 mmol / L (when TG > 2.3 mmol / L)

ii) HDL cholesterol > 1.0 mmol/L for males, > 1.2 mmol / L for females

iii) TG < 1.7 mmol / L

(Ministry of Health Malaysia, 2015)

Planning

- Review lipid profile results.
- Planning of care based on flow chart in Appendix 7, the lipid management of persons with Coronary Vascular Disease (CVD) or Coronary Heart Disease (CHD) risk equivalents.

(Ministry of Health Malaysia, 2011)

Implementation

- Ask questions to identify barriers to dyslipidemia management and clarify on frequently asked questions (FAQ) regarding the management of dyslipidaemia (Committee of the Diabetes Education Manual, 2016 Appendix 8).

Evaluation

- Evaluate the achievement of target lipid level and evaluation check list for dyslipidaemia (Appendix 9).

7.2.3 Smoking Cessation

Assessment

- Assess for current or past smoking behaviour.
- Identify Nicotine dependence using Fagerstrom Test as shown in Appendix 10, Items and scoring for Fagerstrom Test for Nicotine dependence. (Fagerstrom et al, 1990).
- Assess willingness to quit.

Goal Setting

- People with diabetes express interest to reduce smoking behaviour and or able to quit smoking and avoid second hand smoke.

Planning

- Ask for amount of tobacco used on each clinic visit.
- Document tobacco-use status on a regular basis.

Implementation

- Discuss risks of smoking.
- Advice to stop smoking and avoid second hand smokes.
- Refer to quit smoking clinic.

Evaluation

- Obtain feedback on current smoking cessation behaviour.

7.3 Diabetic Nephropathy

Diabetic nephropathy (DN) is the most common cause of Chronic Kidney Disease (CKD). Progression of kidney disease is based on the average decline in eGFR with age as shown in Screening and investigations of CKD in People with Diabetes (Ministry of Health Malaysia, 2011 Appendix 11).

Progression of kidney disease

Average rate of decline of eGFR with age after age 30-40 = 0.4 to 1.2 (0.8) ml/min/1.73 m^2 per year.

Progression of kidney disease = decline in eGFR of >5 ml/min/1.73 m^2 per year or 10 ml/min/1.73 m^2 within 5 years is an indication for referral to a nephrologist (Ministry of Health Malaysia, 2011).

Assessment

- Assess for awareness of the latest renal function test.
- Compare past and current results for change in renal functions.

Goal Setting

- Target recommended BP <130/80 mmHg (SBP range 120-129 mmHg)
  - In people with diabetes having proteinuria ≥1 gm/day
  - In people with diabetes and established diabetic kidney disease

Target HbA1c ≤7% in people with diabetes (individualized according to co-morbidities).

Planning

- Advise screening and investigations for chronic kidney disease at time of diagnosis using Appendix 12 (Ministry of Health Malaysia, 2011).
- Emphasize the importance of annual urine and blood test.
- Explore strategies to delay progression of CKD.

Implementation

- People with diabetes and/or hypertension should be screened at least annually.
- Explain the concept of eGFR by pointing out that in an average person, their GFR declines by about 0.8 ml / min every year after the age of 30-40, and in diabetes, this decline is more rapid (you can use their previous years' eGFR value if available to predict the rate of decline), so they may calculate by age x (e.g. age 60), what their predicted eGFR might be or when they might reach end stage renal failure needing dialysis.
  - Discuss plan to achieve optimal BP and Blood glucose control (Refer hypertension and blood glucose monitoring in Section 5).

Evaluation

- Evaluate the achievement of the target blood pressure and blood glucose level.

7.4 Diabetic Retinopathy

Most significant risk factors for the development of diabetic retinopathy (DR) are hypertension, diabetes, dyslipidemia, increased Body Mass Index (BMI) and smoking. Screening and early treatment can prevent substantial visual loss in many cases.

Assessment

- Examine for awareness for diabetic retinopathy.

Goal Setting

- People with diabetes achieve target control in BP, HbA1c and Total Cholesterol.
- Regular eye follow-up as recommended in follow-up schedule in Appendix 13, Follow-up schedule based on stages of retinopathy (Ministry of Health Malaysia, 2011).

Planning

Screening for Diabetic Retinopathy in Type 2 diabetes at time of diagnosis and if people with diabetes has never had his/her fundus examined.

- Emphasize importance of adhering to follow-up schedule based on the stages of retinopathy to prevent further deterioration of the condition.

Implementation

- Perform screening at time of diagnosis.
- Advise follow-up based on the stages of retinopathy to prevent further deterioration of the condition.

Evaluation

- Evaluate the achievement of the targeted blood pressure and blood glucose level.

7.5 The Diabetic Foot

People with diabetes are at risk for diabetic foot ulcer and delayed wound healing which can lead to amputation. Eighty percent of amputation is preventable with proper foot care. The underlying reasons for diabetic foot are:

- Neuropathy
- Peripheral Arterial Disease
- Combination of neuropathy and peripheral arterial disease (Neuroischaemic).

Assessment

- Identify early the risk factors for foot complications using Appendix 14, Comprehensive Foot Examination and Risk Assessment (Boulton et al, 2008).
- All people with diabetes should receive comprehensive foot examination at diagnosis and at least annually.

Goal Setting

- People with diabetes are able to care for their feet and are aware of the risks of diabetic foot.

Planning

- Provide foot health education for prevention of foot ulcers.

Implementations

- Ensure diabetes foot assessment as per requirement.
- Educate on daily foot care using Diabetes foot: Health education (Appendix 15).
- Refer people with diabetes whose foot are at risk/presence of ulcer to foot care/ wound care division for further care and management.

Evaluation

- Evaluate using the evaluation check list for diabetic foot (Appendix 16).

7.6 Hypoglycaemia

Hypoglycemia refers to blood glucose less than <4.0mmol/L and or presence of neurogenic/autonomic (Adrenegic: Palpitation, tremors, Anxiety/arousal. Cholinergic: cold sweats, hunger, paraesthesia) or (Neuroglycopenic symptoms: Cognitive dysfunction such as behavioural changes, psychomotor abnormalities, seizure, coma, brain damage). In the absence of blood glucose measurement with symptoms of hypoglycaemia which are reversible after food intake. (Ministry of Health Malaysia, 2010).

Assessment

- Identify risk for Hypoglycaemia
    - Concomitant used of insulin segretagogues (Sulphonylurea) and insulin.
    - Elderly
    - Presence of renal or liver impairment.
    - History of recurrent hypoglycaemia.
    - Impaired hypoglycaemia awareness.
    - Behavioural factors such as delayed, skipped or inadequate carbohydrates, unusual exertion, alcohol ingestion, and insulin dosage mishaps, etc.
    - Lack of knowledge in recognizing hypoglycaemia.
- Review knowledge of hypoglycaemia using questionnaire on Hypoglycaemia for people with diabetes. (Task force American Diabetes Association and Endocrine Society, 2013 Appendix 17).

Goal Setting

- People with diabetes are able to achieve target blood glucose without hypoglycaemia.

Planning

- Educate to inform risk for hypoglycemia, recognize signs and symptoms of hypoglycaemia.
- Discuss strategies to prevent hypoglycaemia.

Implementation

- Dietary intervention:
    - Understanding the impact of carbohydrates on blood glucose.
    - Explanation on the consequences of delayed/skipped meals.
    - Ensuring adequate carbohydrates intake.
    - Recommending inter-prandial and bedtime snacks as necessary.
    - Ensuring access to simple carbohydrate.
    - If the person drinks alcohol, advice to consume with meal.

- **Exercise**
  - Check blood glucose prior, during and post exercise as indicated.
  - Advise
    - pre-exercise snacks if blood glucose is <5.5 mmol / L.
    - supplement with carbohydrate during and post exercise if activity is prolonged.
  - Compare BG chart on exercise versus non-exercise day to understand impact of exercise on BG changes.

- **Monitoring**
  - Encourage SMBG before meals, at bedtime and if symptoms of hypoglycaemia occurs.
  - Encourage SMBG between 2 A.M. and 5 A.M. if on insulin therapy.

- **Medications**
  - Advise and explain insulin administration in relation to meals.
  - Advise the use of medic alert (card / bracelet / pendant).

Evaluation

- People with diabetes able to achieve targeted blood glucose without hypoglycaemia.
- If hypoglycemia occurs assess severity of hypoglycaemia and treatment of hypoglycaemia using Appendix 18, Treatment of Hypoglycaemia (Ministry of Health Malaysia, 2011).
- Refer to doctor for further management if:
  - Hypoglycaemia happens >2 times in a week.
  - Hypoglycaemia unawareness.

7.7 References

1. Boulton, A.J.M., Armstrong, D.G., Albert, S.F. et al. (2008) Comprehensive foot examination and risk assessment. *Diabetes Care* Vol. 31, 1679-1685.

2. Committee of the Diabetes Education Manual, Malaysian Diabetes Educator Society (2016) Frequently asked questions (FAQ) regarding management of dyslipidaemia.

3. Fagerstrom, K.O., Heatherton, T.F. and Kozlowski, L.T. (1990) Nicotine Addiction and Its Assessment. *Ear, Nose and Throat Journal* Vol.69, 763-765.

4. Ministry of Health Malaysia (2011) *Clinical Practice Guidelines in Management of Dyslipidemia* (4th Edition).

5. Ministry of Health Malaysia (2011) *Clinical Practice Guidelines in Management of Chronic Kidney Disease in Adults.*

6. Ministry of Health Malaysia (2011) *Clinical Practice Guidelines for Screening of Diabetic Retinopathy.*

7. Ministry of Health Malaysia (2010) *Practical Guide to Insulin Therapy in Type 2 Diabetes Mellitus.*

8. Ministry of Health Malaysia (2013) *Clinical Practice Guidelines on the Management of Hypertension* (4th Edition).

9. Ministry of Health Malaysia (2015) *Clinical Practice Guidelines on the Management of Type 2 Diabetes* (5th Edition).

10. Seaquist, E.R., Anderson, J., Childs, E. et al. (2013) Hypoglycemia and Diabetes: A report of a workgroup of the American Diabetes Association and the Endocrine Society. *Diabetes Care* Vol. 36, 1384-1395.

7.8 Appendices

Appendix 1 Clinical Monitoring Schedule

Test	Initial visit	3 monthly-visit	Annual visit
Weight			
Waist circumference			
BMI			
Blood Pressure			
Eye: visual acuity			
Fundoscopy			
Feet: pulses			
Neuropathy			
Dental Check-up		6-monthly	
Blood glucose			
A1c			
Cholesterol / HDL Cholesterol			
Triglycerides			
Creatinine / BUSE			
Liver function test			
Urine microscopy			
Albuminuria*			
ECG**			

conduct test
conduct test if abnormal 1st visit or symptomatic
no test required

* Microalbuminuria if resources are available

** At initial visit and if symptomatic

(Adapted: Clinical Practice Guidelines Management of Type 2 Diabetes Mellitus: 5th edition, Malaysia Ministry of Health, 2015)

Appendix 2 Management of Hypertension

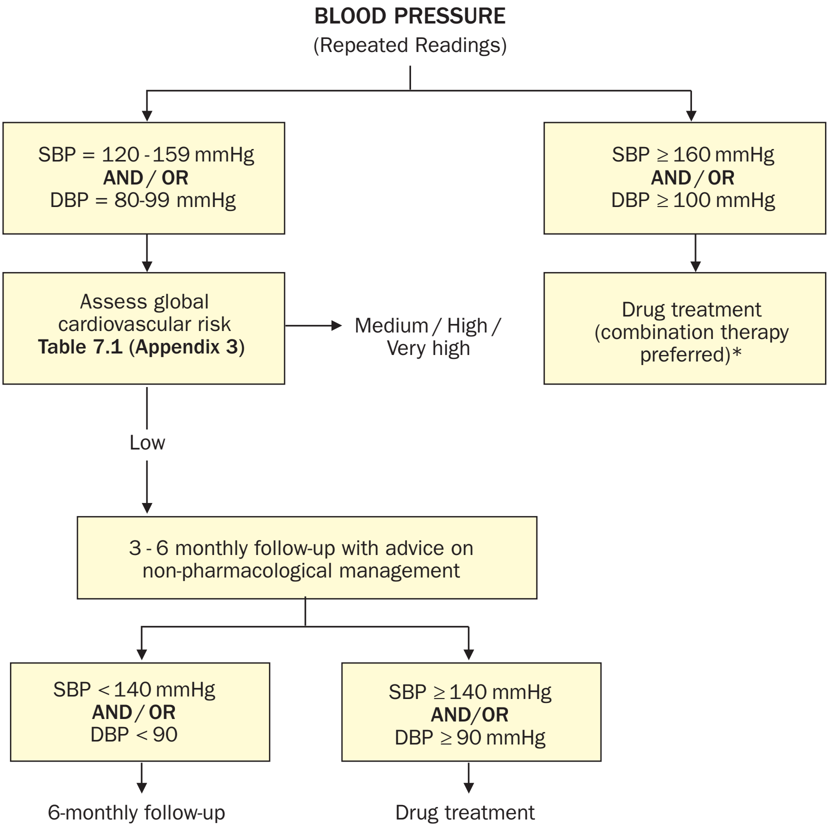

*either free or single pill combination (free pill combination or single pill combination will improve adherence and it reduces the total overall health cost).

(Adapted: Clinical Practice Guidelines Management of Hypertension, Malaysia Ministry of Health, 2013)

Appendix 3: Risk Stratification

Co-existing Condition / BP Levels (mmHg)	No RF No TOD No TOC	TOD or RF (1-2) No TOC	TOC or RF (≥3) or Clinical atherosclerosis	Previous MI or Previous stroke or Diabetes
SBP 130-139 and / or DBP 80-89	Low	Medium	High	Very high
SBP 140-159 and / or DBP 90-99	Low	Medium	High	Very high
SBP 160-179 and / or DBP 100-109	Medium	High	Very high	Very high
SBP >180 and / or DBP >110	High	Very high	Very high	Very high

Risk level	Risk of Major CV Event in 10 years	Management
Low	<10%	Lifestyle changes
Medium	10 - 20%	Drug treatment and lifestyle changes
High	20 - 30%	Drug treatment and lifestyle changes
Very high	>30%	Drug treatment and lifestyle changes

TOD = Target organ damage (Left Ventricular Hypertrophy [LVH], retinopathy, proteinuria).

TOC = Target organ complications (heart failure, renal failure).

RF = additional risk factors (smoking, TC >6.5 mmol/L, family history of premature vascular disease).

Clinical atherosclerosis (CHD, carotid stenosis, peripheral vascular disease, transient ischaemic attack, stroke).

(Adapted: Clinical Practice Guidelines Management of Hypertension, Malaysia Ministry of Health, 2013).

Appendix 4: Action Plan for Blood Pressure and Lipid Control

1. Lifestyle Intervention

1.1 Weight management: reduce weight, for individual with BMI > 23.0 kg/m^2.

1.2 Diet management

- Emphasizes vegetables, fruits, and fat-free or low-fat dairy products.
- Includes wholegrains, fish, poultry, beans, seeds, nuts, and vegetable oils.
- Limits sodium, sweets, sugary beverages, and red meats.
- Reduction of saturated fat, trans fat, and cholesterol intake.
- Increase intake of omega-3 fatty acids, viscous fiber, and plant stanols/sterols (such as in oats, legumes, and citrus).

2. Exercise

- 150 mins/week of moderate-intensity aerobic physical activity (50-70% of maximum heart rate).
- Spread over at least 3 days/week with no more than 2 consecutive days without exercise.
- Refer to Section 4: Physical Activity and Exercise chapter for more details on exercise in diabetes management.

3. Pharmacotherapy for People with Diabetes, Hypertension and Dyslipidaemia

- If the targeted blood pressure and lipid profile not achieved or in the presence of microalbuminuria or overt proteinuria, refers to doctor for pharmacology intervention.
- Statin therapy should be added to lifestyle therapy for people with diabetes of all ages and overt CVD (Refer Section 5: Medications for Type 2 diabetes).

4. Monitoring

- Monitor blood pressure at each visit.
- Report of any uncontrolled hypertension B/P > 130/80 mmHg.
- Review home monitoring blood pressure record if available.

Appendix 5

Diabetes Education using Two Way Dialogue and Decisional Balance Box

1. Approach to People with Diabetes: Two way dialogue

(Diabetes education is recommended as a two way dialogue. ***Always ask before offering your recommendation or suggestion, using the sequence 'Ask – Give – Ask'****) e.g.*

(ASK☺)*: "What ways do you know of getting your BP down?'*

(GIVE☺)*: "Some of my patients have found not putting soya sauce/salt on the dining table useful."*
"Have you thought about going back to your doctor to tell him/her that you are getting these side effects from the medication?"

2. Decisional Balance Box

DECISIONAL BALANCE BOX

	**Pros (Good things about doing this)**	**Cons (Less good things about doing this)**
Continue as before (No Change)		
New or Changed Behaviour		

– Look at the pros of the current behaviour before examining the cons
– Go on to ask questions about the pros about change and then the cons
– Summarise both sides of the change position and ask the patient

Appendix 6

Evaluation Check List for Hypertension

No	Items	Yes / No
1	**Knowledge on antihypertensive medications. People with diabetes are able to identify:**	
	• Name and indications	
	• Dosage	
	• Frequency	
	• Timing (e.g. take morning dose with small amount of water despite fasting for blood test)	
2	**Lifestyle intervention**	
	• able to list current issues for improvement	
	• able to make informed decision	
	• Plan and agree on action plan	
3	**Blood pressure control**	
	• Understand and verbalize target	
	• Achieved blood pressure target	

Appendix 7

Lipid Management of Persons with CVD or CHD Risk Equivalents

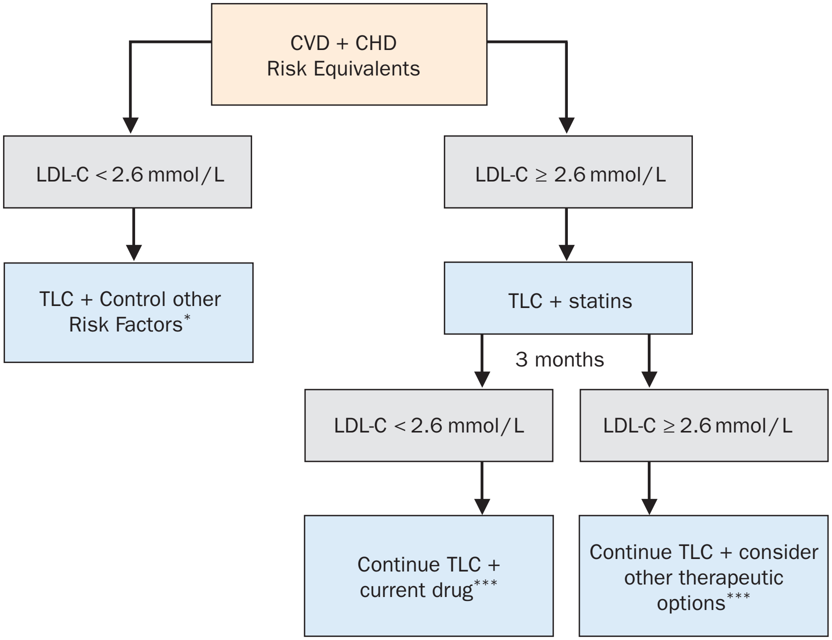

TLC – Therapeutic Lifestyle changes

* Start statins to achieve LDL - C target goal < 2.0 mmol/L

** Consider LDL-C target goal < 2.0 mmol/L in very high risk individuals eg individuals with ACS, recurrent cardiac events, CHD with T2DM and those with multiple poorly controlled risk factors

*** Other therapeutic options include increasing dose of statin, changing to high intensity Statin or combination therapy intensifying diet therapies, weight reduction, exercise oradding drugs to lower TG and/or increase HDL-C

(Adapted and modified from ATP III in Guidelines in the Management of Dyslipidaemia, Malaysia Ministry of Health, 2011)

Appendix 8

Frequently Asked Questions (FAQ) Regarding Management of Dyslipidaemia (Committee of the Diabetes Education Manual, 2016).

Q: What is the benefit of lowering my cholesterol especially LDL-C levels?

A: Having diabetes is considered a coronary risk equivalent, i.e. about 20% or more chances of getting CVD in the next 10 years. For people with diabetes, taking a statin will significantly reduce the risk of getting CVD, i.e. for every 6 to 11 people with diabetes taking a statin, one will be prevented from getting CVD.

Q: I feel body ache after taking statins, once I stop my body ache is gone.

A: The most common side effect of statin is muscle pain. People with diabetes may feel this pain as a soreness, ache or weakness in their muscles. Most of the time, they are mild and do not limit their activities. Rarely, statins can cause clinically important myositis and rhabdomyolysis (life-threatening muscle and liver damage, kidney failure). Note: If people with diabetes experience severe muscle pain, they should stop the statin and see their doctor immediately. If they experience mild muscle pain, they should discuss with their doctor as they may be able to reduce the dose, review their medications or switch to a different statin.

Q: Will statins bring harm to my body?

A: There is a balance between risks and benefits. The doctor will monitor the liver function test (for signs of inflammation) and signs of muscle pain. For people with diabetes the benefits of statin far outweigh the risks.

Q: Can I stop my cholesterol medications when my cholesterol level is back to normal?

A: Unless people with diabetes have modified their diet and lifestyle dramatically, for most people, the cholesterol level will go up again once they stop the statins.

Q: I don't want medications, could I take supplement like omega fish oil/krill oil instead?

A: Supplements like omega fish oil can lower the triglyceride, but not the LDL cholesterol. Beta-Glucan, plant stenols, and high fiber diet can lower cholesterol moderately in conjunction with a healthy diet includes reduced saturated fat, trans-fat and animal fat. People with diabetes could discuss this further with the dietician.

Appendix 9

Evaluation Check List on Dyslipidaemia

No	Items	Yes / No
1	**Knowledge on dyslipidaemia. People with diabetes are able to:**	
	• List individual targets for LDL-C, HDL-C and Triglycerides	
	• Understand dyslipidaemia as one of the risk for CVD	
	• Identify name, dosage, timing of dyslipidaemia medication	
2	**Lifestyle interventions**	
	• List current lifestyle behaviour for improvement	
	• Plan and agree on action plan for change	
	• Identify and discuss barriers for change	
3	**Monitoring**	
	• Repeat lipid profile at least annually or more often if needed to achieve targets	

Appendix 10

Item and Scoring for Fagerstrom Test for Nicotine Dependence (FTND) (Fagerstrom K.O. et al, 1990).

No	Questions	Answers	Points
1	How soon after you wake up do you smoke your first cigarette?	Within 5 minutes<br>6-30 minutes	3<br>2
2	Do you find it difficult to refrain from smoking in places where it is forbidden, e.g. in places of worship, at the library, in cinema etc.?	Yes<br>No	1<br>0
3	Which cigarette would you hate most to give up?	The first one in the morning<br>All others	1<br>0
4	How many cigarettes/day do you smoke?	10 or less<br>11-20<br>21-30<br>31 or more	0<br>1<br>2<br>3
5	Do you smoke frequently during the first hours after waking than during the rest of the day?	Yes<br>No	1<br>0
6	Do you smoke if you are so ill that you are in bed most of the day?	Yes<br>No	1<br>0

Score ranges from very low dependence to very high dependence, (0-2) very low, (3-4) low, (5) medium, (6-7) high and (8-10) very high.

Appendix 11: Screening and Investigations of CKD in Patients with Diabetes

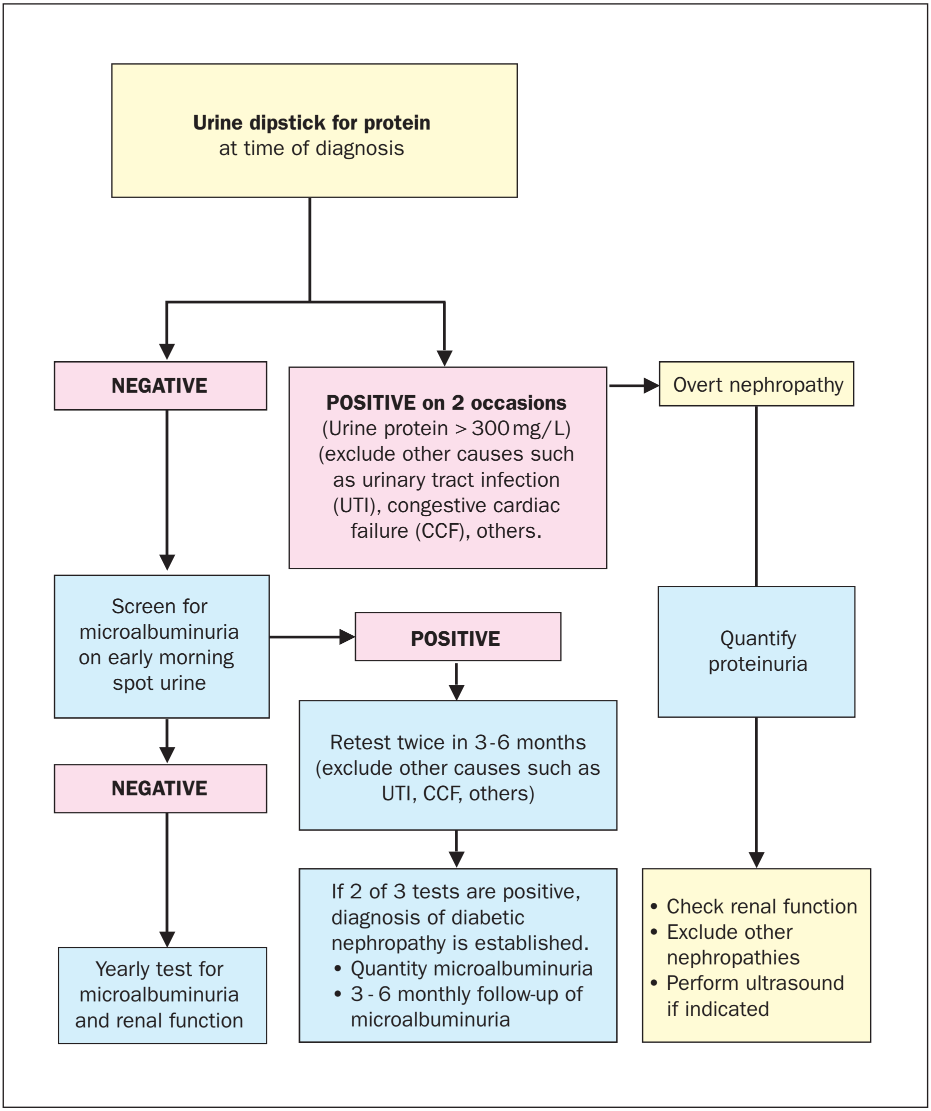

(Adapted and modified from Clinical Practice Guideline in Management of CKD in adults Ministry of Health Malaysia, 2011)

Appendix 12

Stages of Chronic Kidney Disease

Stages of Chronic Kidney Disease		
**Stage**	**GFR (ml/min/$1.73m^2$)**	**Description**
1	≥90	Normal or increased GFR, with other evidence of kidney damage
2	60-89	Slight decrease in GFR, with other evidence of kidney damage
3A	45-59	Moderate decrease in GFR, with or without other evidence of kidney damage
3B	30-44	
4	15-29	Severe decrease in GFR, with or without other evidence of kidney damage
5	<15	End stage renal failure/ dialysis

(Adapted from Clinical Practice Guideline in Management of CKD in adults Ministry of Health Malaysia, 2011)

Appendix 13

Follow-up Schedule Based on Stages of Retinopathy

Stage of Retinopathy	Follow-up
No Diabetic Retinopathy (DR)	12-24 months
Mild Non-proliferative Diabetic Retinopathy (NPDR)	9-12 months
Moderate NPDR without maculopathy	6 months
Severe NPDR without maculopathy	Refer ophthalmologist
Any maculopathy	
Proliferative DR	Refer urgently to ophthalmologist
Advanced Diabetic Eye Disease (ADED)	
No DR or Mild NPDR in pregnant women	Every 3 months
Moderate or worse NPDR in pregnant women	Refer ophthalmologist

(Adapted from Clinical Practice for Screening of Diabetes Retinopathy, Ministry of Health Malaysia, 2011)

Appendix 14

Comprehensive Foot Examination and Risk Assessment

Risk category	Definition	Treatment	Suggested Follow-up
0	No loss of protective sensations (LOPS)<br><br>No Peripheral Artery Disease (PAD)<br><br>No deformity	Patient education including advice on footwear	Annually by generalist/ foot specialist
1	LOPS ± deformity	Consider prescriptive or accommodative footwear<br><br>Consider prophylactic surgery if deformity is not able to be safely accommodated in shoes<br><br>Continue patient education	Every 3-6 months by generalist or foot specialist
2	PAD and LOPS	Consider prescriptive or accommodative foot wear<br><br>Consider vascular consultation and combined follow-up	Every 2-3 months by foot specialist
3	History of ulcer or amputation	Same as category 1<br><br>Consider vascular consultation for combined follow-up if PAD present	Every 1-2 months by foot specialist

(Adapted from Risk Classification based on the comprehensive foot examination Boulton et al, 2008)

Appendix 15

Diabetes Foot: Health Education

1. Daily Self-inspection

- On both feet, pay attention to space between your toes (web space).
- Check the plantar aspect of both feet, use mirror if unable to bend down.
- Ask for assistance if poor vision.
- Ensure sufficient lighting while doing self-inspection.

2. Foot wears

- Shoes should be wide and deep at the toe, the shoe should be 1 cm to 1.5 cm longer than the foot.
- Shoes should have a thick rubber sole; a leather sole is not advisable.
- Do not use tong slipper, high heel shoes.
- Covered shoes are advisable.
- The back of the shoe, the ‘heel counter’, should be firm enough to provide support for the foot.
- To cope with swelling, shoes should have a lace-up or velcro fastening.
- When choosing shoes, feel inside to check for rough seams.
- Safety boots must be fitted carefully.
- Elastic around the leg should not be too tight as this cut in to the leg and reduces circulation.
- Use cotton socks of the correct size (Socks absorb sweat and prevent friction so that blisters are less likely to occur).
- Socks should be washed and changed every day.

3. General care of the feet

- Wash and dry feet daily.
- All inter-digital web-space must be kept dry at all time.
- Apply non-perfume based moisturizer daily to prevent dryness, but not in between toes.
- Never use corn cures plaster/salicylate acid plaster.
- Callous/corns should be trim by trained personnel.
- Nail trimmings
  - Wash and clean feet before nail trimming.
  - Cut straight across, file sharp edges.
  - Seek assistance when eyesight is poor, thickened or abnormal toe nails i.e. involuted nails.
- NOT to walk barefooted, wash/soak feet with hot/warm water, hot water bottle or infrared lights.

Appendix 16

Evaluation Check List for Diabetic Foot

No	Items	Date	Date
1	**Client able to take action to reduce risk of diabetic foot ulcer**		
	• Check for foreign objects in shoes before wearing them.		
	• Wear appropriate foot wears at outdoor and or indoors.		
	• Identify and report foot problems to health care provider.		
	• Maintain feet hygiene, free from fungal infection.		
	• Understand risk for diabetic foot injury i.e. foot reflexology, heat therapy, poor fitting foot wears.		
2	**Monitoring**		
	• Performing daily foot inspection.		
	• Repeat foot assessment at least annually or more often if needed to prevent diabetic foot ulcer.		

Appendix 17

Questionnaire on Hypoglycaemia for People with Diabetes

Name: ____

First Middle Last

Today's date:______________________________

1. To what extent can you tell by your symptoms that your blood glucose is LOW?

   Never _______Rarely _______ Sometimes _______Often _______ Always _______

2. In a typical week, how many times will your blood glucose go below 70 mg/dL (3.9 mmol/L)?

   _________ a week.

3. When your blood glucose goes below 70 mg/dL (3.9 mmol/L) what is the usual reason for this?

   ____

4. How many times have you had a severe hypoglycaemic episode (where you needed someone's help and were unable to treat yourself)?

   Since the last visit _________ times

   In the last year _________ times

5. How many times have you had a moderate hypoglycaemic episode (where you could not think clearly, properly controlled your body, had to stop what you were doing, but you were still able to treat yourself)?

   Since the last visit _________ times

   In the last year _________ times

6. How often do you carry a snack or glucose tablets (or gel) with you to treat low blood glucose?

   Check one of the following:

   Never ______ Rarely______ Sometimes ______ Often ______ Almost always _______

7. How Low does your blood glucose need to go before you think you should treat it?

   Less than _________ mg/dL (Less than _________ mmol/L)

8. What and how much food or drink do you usually treat low blood glucose with?

   ____

9. Do you check your blood glucose before driving? Check one of the following:

   Yes, always _________ Yes, sometimes _________ No _________

10. How Low does your blood glucose need to go before you think you should not drive?

    _________ mg/dL (_________ mmol/L)

11. How many times have you had your blood glucose below 70 mg/dL (3.9 mmol/L) while driving?

    Since the last visit _________ times

    In the last year _________ times

12. If you take insulin, do you have a glucagon emergency kit?

    Yes _________ No _________

13. Does a spouse, relative, or other person close to you know how to administer glucagon?

    Yes _________ No _________

(Adapted from Seaquist et al. 2013)

Appendix 18

Treatment of Hypoglycaemia

1. Assess the cause and severity of hypoglycaemia.

2. Treat hypoglycaemia according to blood glucose (BG) level.

    - **Mild (BG 3.3 – 3.9 mmol/L):** Give 15 g carbohydrate
      4 ounces (120 mls) orange juice or other fruit juices **OR**
      2-3 pieces of soft candy

    - **Moderate (BG 2.5 – 3.2 mmol/L):** Give 20 g carbohydrate
      6 ounces (180 mls) orange juice or other fruit juices **OR**
      4 glucose tablets **OR** Dextrose 50% 25 ml intravenous

    - **Severe (BG <2.5 mmol/L):** Give 30 g carbohydrate
      8 ounces (240 mls) orange juice or other fruit juices **OR**
      6 glucose tablets **OR** Dextrose 50% 25 ml intravenous

    - **Unconscious with severe hypoglycaemia (BG < 2.5 mmol/L):**
      Nil by mouth
      For vomiting and aspiration risk, roll patients onto their side
      Dextrose 50% 25 ml intravenous **OR** Glucagon 1 mg subcutaneous or intramuscular

3. Monitor BG level every 15 minutes and repeat the above until blood glucose reach 5.6 mmol/L.

4. Educate for prevention of hypoglycemia.

*Note: Glucose tablet and or Glucagon may not be available locally.

(Adapted from Ministry of Health Malaysia, 2010)

SECTION 8

BEHAVIOURAL INTERVENTION

No.	Content	Page
8.1	Introduction	125
8.2	Definition	125
8.3	Aims	125
8.4	Theoretical Approaches to Behaviour Change	125
8.5	Transtheoretical Model of Change	125
8.6	General Recommendations	127
8.7	Specific Considerations	127
8.8	Assessment	127
8.9	Goal Setting	129
8.10	Planning	130
8.11	Implementation	130
8.12	Follow up Session	130
8.13	Evaluation and Monitoring	132
8.14	Referral to Other Healthcare Professionals	132
8.15	References	133
8.16	Appendix	134

8.1 Introduction

Diabetes management is complex. It requires lifestyle modification needing considerable effort to change and maintain. Behaviour intervention is vital in adhering to this change.

8.2 Definition

The aim of behaviour intervention is to identify which behaviours are targeted for change and how change will be managed. It may include ways of providing positive reinforcement, changes in the environment and necessary support so that people with diabetes will not react negatively due to frustration or fatigue.

8.3 Aims

Behaviour intervention is introduced to improve the quality of life, to detect challenges early, and to manage symptoms through less costly approaches. It must be integrated into the person's treatment regimen to maximise its intended / beneficial effects.

8.4 Theoretical Approaches to Behaviour Change

Not everyone with diabetes will benefit from traditional advice and education; physicians need to tailor intervention individually by understanding and identifying the person's position in the change process (Zinmerman et al, 2000). There are five well developed change theories in diabetes self-management namely Health Belief Model, Social Cognitive Theory, Theory of Reasoned Action and Theory of Planned Behaviour, Empowerment Theory and Transtheoretical Model (TTM) (Funnell and Anderson, 2004; Prochaska and DiClemente, 1983). In this manual, TTM is the method used to describe behaviour through the five stages of change over a period of time. TTM is also a widely used tool in many areas of health changing behaviour (Funnel et al, 2014).

8.5 Transtheoretical Model of Change (TTM)

TTM was developed by Prochaska and DiClemente (1983). It facilitates health practitioners in providing help to people with diabetes by identifying their current stage of change as well as assisting and motivating the person to progress to the next stage which is shown in Figure 1 and Table 1 below.

Figure 1: Stages of Transtheoretical Model of Change

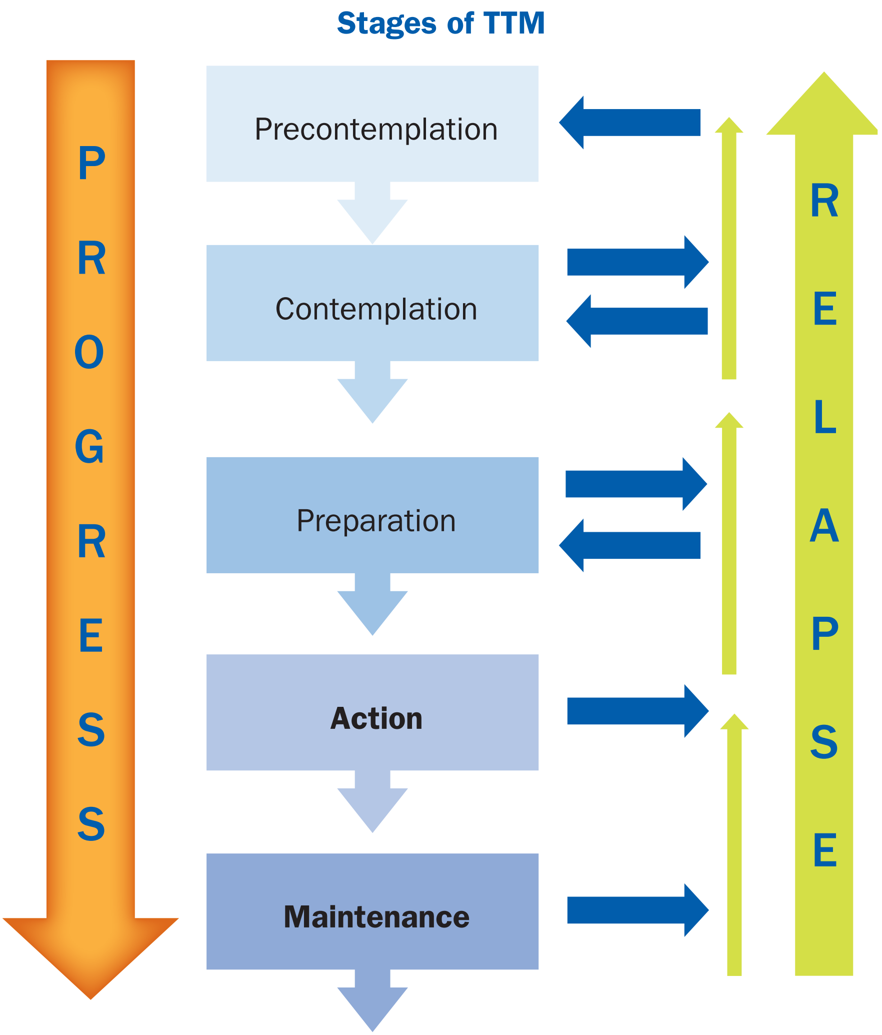

Table 1: Prochaska and DiClemente's Stages of Change Model

Stage of Change	Description
Pre contemplation	A person is not currently thinking of changing or adopting a new behaviour in the next six months.
Contemplation	A person is considering to change or adopt a new behaviour within the next six months.
Preparation	A person is ready to change and set goals. It involves planning thoughts and action and making specific plans about change within 2 to 3 months.
Action	A person has made specific overt modifications in his/her life-style within the past six months.
Maintenance	A person is working to prevent relapse but he/she does not apply change processes as frequently as people in action.
Relapse	A person may fall or slip back into a former stage.

8.6 General Recommendations

Behaviour intervention is recommended to empower and facilitate people with diabetes to self-manage daily living such as adherence to prescribed diet, medication, physical activity and self-monitoring including self-monitoring of blood glucose, body weight and blood pressure.

8.7 Specific Considerations

People with diabetes with the following conditions need to be referred for specialized care:

- Prior psychological and psychiatric conditions e.g. eating disorders, depression and anxiety

8.8 Assessment

Evaluating socio-demographic and economic background (Refer to the Initial Assessment in Section 2).

Explore behaviour issues by:

1. Assessing their perception of the condition
    - What do you know about diabetes?
    - Can you share with me your understanding/perception about diabetes?
2. Assessing their perception on the importance to change
    - What brings you here today?
    - What do you want in your life?

3. Assessing their readiness to change in the following areas:
   - Physical Activity
   - Nutrition and Diet
   - Self-monitoring of blood glucose
   - Medication adherence
   - Managing emotional stress
   - Follow up for Diabetes management
   - Foot care
   - Assessment for complications

4. Assessment of Stages of Change

   You may use the statements below to assess the person's stage of change. Refer to Appendix 1

   a. No, I do NOT intend to in the next 6 months.

   b. No, I intend to in the next 6 months.

   c. No, but I intend to in the next 30 days.

   d. Yes, I have been, but for less than 6 months.

   e. Yes, I have been for more than 6 months.

5. Assessing their confidence to change
   - How confident are they in achieving their goal(s)?
   - Ask them to rate themselves on a scale of 0 (not able to achieve) -10 (definitely able to achieve)

6. Explore issues that prevent them from achieving a higher score (i.e. assess their challenges to change)
   - What are the challenges faced while trying to implement change?
   - What stopped/prevented them from changing?
   - What support/assistance do they require to change?
   - Assessment of person's expectations from their healthcare providers (HCP)

7. Assess their expectations from their HCPs
   - What do they hope to get from HCPs?
   - How can HCPs help them?

(Website: www.projectstay.com/pdf/BehaviourInterventionPlan.pdf)

8.9 Goal Setting

Goal setting should be set in collaboration with the person with diabetes. Goal setting should be:

S – Specific

M – Measurable

A – Achievable and affordable

R – Relevant

T – Time specific

Example of goal setting Using SMART:

To start an exercise programme

**S**	Specific	Walking 30 min per day
**M**	Measurable	5 days per week
**A**	Achievable	Gradually increase the duration of walking
**R**	Relevant	To lose 5% of current weight and thus improve blood glucose
**T**	Time specific	1 month

8.10 Planning

Develop a plan with the person with diabetes and the caregiver according to the person's expectations. During development of the plan, healthcare professionals may want to discuss with the person by asking the following questions:

- What would you want to achieve in
  a) short term (e.g.: 1 - 3 months)
  b) long term (e.g.: within 12 months)
- How can you help yourself?
- What are the steps that you can take to change / help yourself?
- How would you like to start?
- What are the possible challenges that you may face?
- What would happen if you do not make any changes now? (i.e. the consequences of not making any changes)
- Where can you seek help? (e.g. organizations, support groups)
- From whom can you seek help? (e.g. family members, friends, social workers)
- How else can I help you? (i.e. issues that are not addressed previously)

8.11 Implementation

- Discuss and agree on mutual goals.
- Discuss and decide on a plan to achieve the goal.
- Keep a logbook to record changes towards the mutually agreed goals.
- Discuss sources of social support with people with diabetes (e.g. family / friends / social workers / support groups / colleagues) if necessary.
- Apply behavioural techniques at different stages of change as shown in Table 2.

8.12 Follow-up Session

In follow-up sessions:

- Explore their feelings during the implementation of their plans.
- Discuss any issues raised.
- Provide appropriate support.
- Make changes to the previous plan according to their needs.

Table 2: Behavioural Techniques at Different Stages of Change

Stage of Change	Techniques
Pre contemplation	• Listen and validate their situation.<br>• Encourage self-exploration of current situation.<br>• Explain and personalize person's risk of not making the change.<br>• Provide information and pamphlets.
Contemplation	• Validate their efforts in considering to change.<br>• Clarify their perception of change.<br>• Encourage self-exploration.<br>• Evaluate pros and cons of behaviour change.<br>• Identify and promote new, positive outcome expectations.
Preparation	• Praise their efforts.<br>• Prioritize behaviour change.<br>• Identify and assist in problem solving.<br>• Help identify social support.<br>• Verify that the person has skills for behaviour change.<br>• Encourage small initial steps.
Action	• Continue support.<br>• Reinforce their efforts.
Maintenance	• Plan for follow-up support.<br>• Reinforce internal rewards.<br>• Prevent relapse.
Relapse	• Evaluate trigger for relapse.<br>• Discuss coping with relapse.<br>• Reassess motivation and barriers.<br>• Plan stronger coping strategies.

8.13 Evaluation and Monitoring

- Diabetes logbook – healthcare professionals monitor the logbooks e.g. SMBG results through SMS, food record with pictures taken through smartphone.
- Ask the person with diabetes to rate their level of achievement on a scale of 0-10 in achieving their goal? (0 means no change at all, 10 means has achieved the goal).
- If they are not achieving their set goals, identify and discuss the challenges and refer to the appropriate healthcare professional if necessary.

8.14 Referral to Other Healthcare Professionals:

- Emotional problems affecting their daily car - refer to clinical psychologist/counsellor.
- Knowledge deficit refer to educators (dietician, nurse educator, pharmacist, physiotherapist).
- Medical problems refer to physician.
- Social problems refer to social worker.

8.15 References

1. Funnell, M.M. and Anderson, R.M. (2004) Empowerment and Self- Management of Diabetes. *Clinical Diabetes* Vol. 22, 123-127.

2. Funnell, M.M., Piatt, G.A. and Anderson, B. (2014) Theoretical and behavioral approaches to the self-management of health. In Mensing, C., Cornell, S., and Halstenson, C. (3rd ed.), *The art & science of diabetic self-management education desk reference* (pp. 89-116). Chicago: American Association of Diabetes Educators.

3. Prochaska, J.O. and DiClemente, C.C. (1983) Stages and processes of self-change of smoking: Toward an integrative model of change. *Journal of Consulting and Clinical Psychology* Vol. 51, 390-395.

4. What is Behavioural Intervention Plan? (2015). Website: www.projectstay.com/pdf/BehaviourInterventionPlan.pdf. Accessed on 19 May 2015.

5. Zimmerman, G.L., Olsen, C.G. and Bosworth, M.F. (2000) A 'stage of change' approach to helping patients change behaviour. *American Family Physician* Vol. 61, 1409-1416.

8.16 Appendix 1: Algorithm for Behavioural Intervention

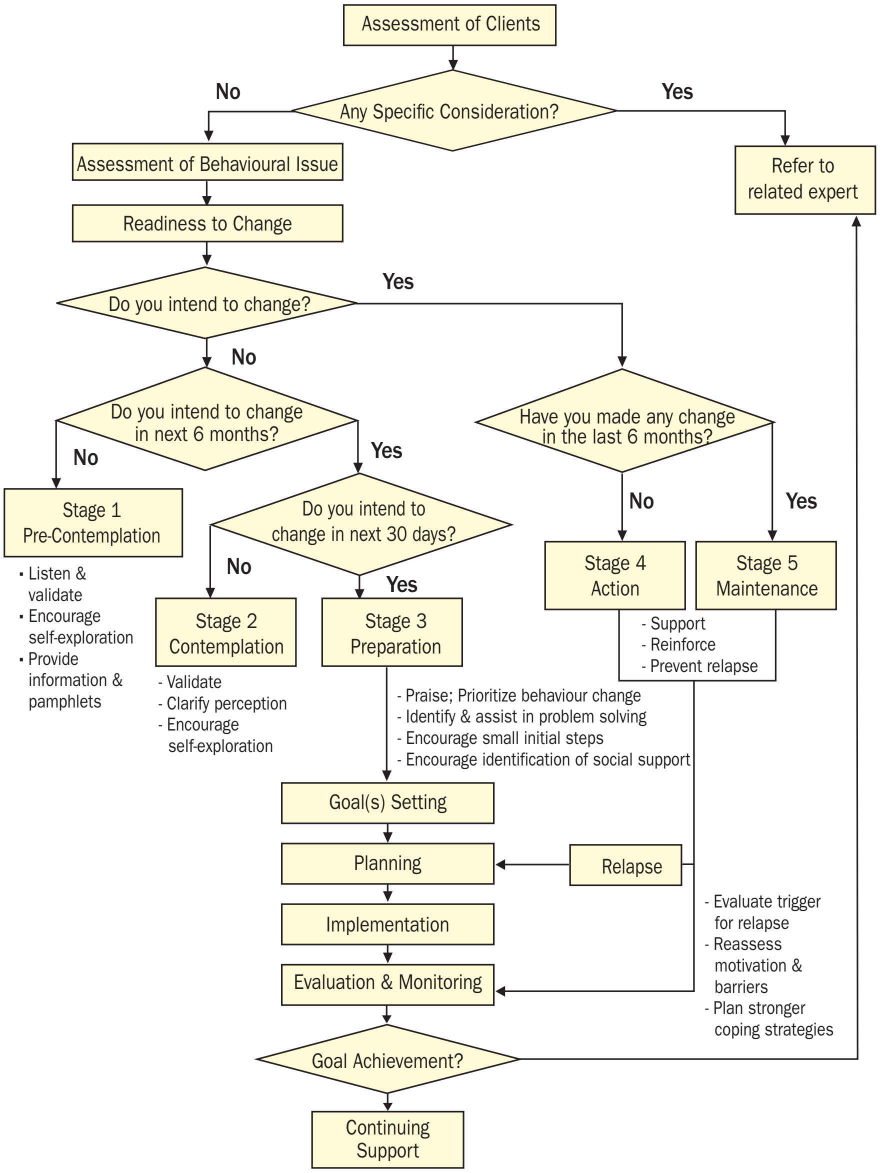

GLOSSARY OF TERMS

Abbreviation	Terminology
ADA	American Diabetes Association
AACE	American Association Clinical Endocrinologists
AGI	α-glucosidase Inhibitor
ACE	Angiotensin converting enzyme
ACEI	Angiotensin converting enzyme inhibitor
ARB	Angiotensin receptor blocker
ACR	Albumin Creatinine ratio
ADED	Advanced Diabetic Eye Disease
BMI	Body Mass Index
BD	Twice daily
BG	Blood Glucose
BP	Blood Pressure
CHO	Carbohydrate
CV	Cardiovascular
CVD	Cardiovascular disease
CKD	Chronic kidney disease
CCF	Congestive cardiac failure
CCB	Calcium channel blocker
CPG	Clinical Practice Guideline
DPP-4	Dipeptidyl peptidase 4
Dr	Doctor
DM	Diabetes Mellitus
DSME	Diabetes self-management education
D.N.	Diabetic nephropathy
D.R.	Diabetic retinopathy
eGFR	Estimated Glomerular Filtration Rate
FBS	Fasting blood sugar
GI	Gastrointestinal
GIT	Gastrointestinal tract
GLP-1	Glucagon like peptide-1
GP	General Practitioner
HDL	High-density Lipoprotein
HDL-C	High-density Lipoprotein Cholesterol

HbA1c	Glycosylated haemoglobin
HF	Heart failure
HCP	Healthcare providers
IDF	International Diabetes Federation
IHD	Ischaemic heart disease
LDL	Low Density Lipoprotein
LDL-C	Low Density Lipoprotein-Cholesterol
LOPS	Loss of protective sensation
MNT	Medical Nutrition Therapy
MOA	Mechanism of action
MOH	Ministry of Health
NPH	Neutral Protamine Hagedorn
NRT	Nicotine replacement therapy
NPDR	Non-proliferative diabetic retinopathy
OAD	Oral Anti-Diabetic
OD	Once daily
OM	Every morning
ON	Every night
PAR-Q	Physical Activity Readiness Questionnaire
SGLT 2	Sodium-glucose cotransporter 2
SR	Slow release
SMBG	Self-Monitoring Blood Glucose
SMART	Specific, Measurable, Achievable, Relevant, Time specific
SMS	Short Message Service
SBP	Systolic blood pressure
TDS	Three times a day
TC	Total cholesterol
TG	Triglyceride
T2DM	Type 2 Diabetes Mellitus
TTM	Transtheoretical Model of Change
UTI	Urinary tract infection
2HPP	2 hour post prandial

ACKNOWLEDGEMENTS

We would like to extend our gratitude and appreciation to the following for their contributions:

- Ministry of Health Malaysia for their support in the development of the manual
- Panel of internal (Dr. Arlene Ngan, Ms Lee Lai Fun) and external reviewers for their time and professional expertise
- Johnson and Johnson Sdn. Bhd. for the use of the meeting premises
- Dr. Feisul Idzwan Mustapha for the support and advice throughout the development of the manual
- All those who have contributed directly or indirectly to the development of this manual

Source of Funding

This manual is funded by the Malaysian Diabetes Educators Society.

PERSATUAN PENDIDIK DIABETES MALAYSIA
(Malaysian Diabetes Educators Society)
