# Supplementary material for: The effect of USM-IAM-based counselling vs standard counselling on insulin adherence, FBS and HbA1c among patients with uncontrolled type 2 diabetes mellitus (T2DM): a randomised controlled trial
Source: BMC Endocr Disord. 2024 Jul 18;24:118. doi: 10.1186/s12902-024-01577-6 (PMC11256455; doi:10.1186/s12902-024-01577-6)
Supplement: Supplementary file 4 — Supplementary Material 4. [file 12902_2024_1577_MOESM4_ESM.pdf]

UNIVERSITI SAINS MALAYSIA
APEX

MODUL PENDIDIKAN PATUH INSULIN UNTUK PESAKIT DIABETES JENIS 2

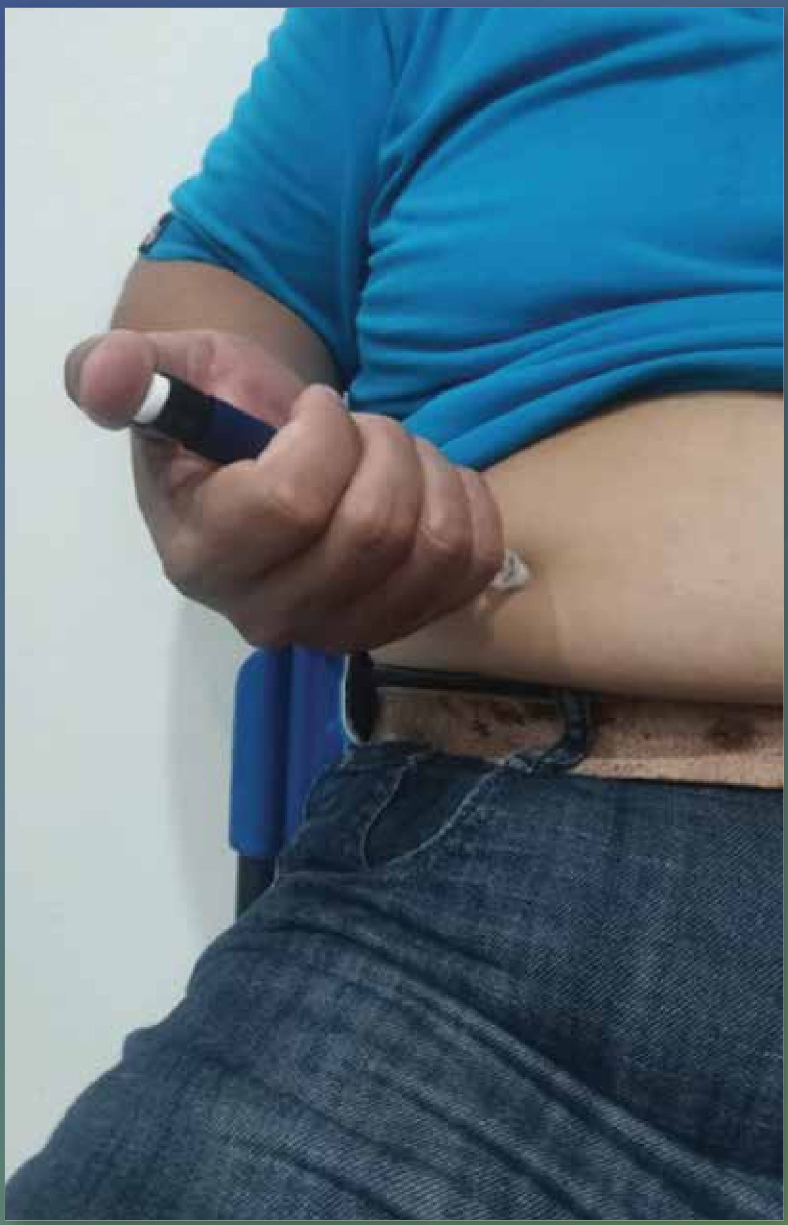

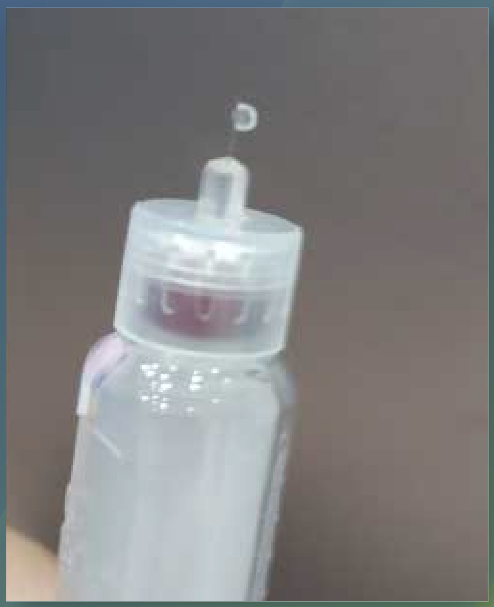

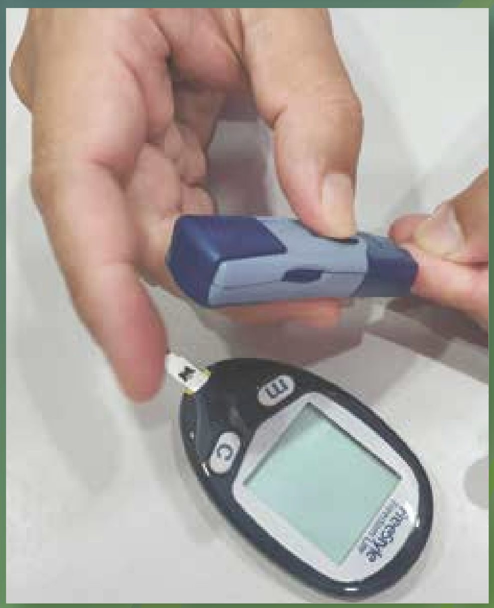

Aida Maziha Zainudin
Wan Mohd Izani Wan Mohamed
Rosediani Muhamad
Aida Hanum Ghulam Rasool
Mohd Zarawi Mat Nor
Norul Badriah Hassan

Kandungan

Pengenalan ii

Penghargaan iii

BAHAGIAN 1 Diabetes dan insulin...1

1.1 Definisi diabetes 1

1.2 Jenis-jenis diabetes...1

1.3 Apakah hubungan antara diabetes dan insulin?......................2

1.4 Jenis- jenis insulin 3

1.5 Mengapa pesakit diabetes menyuntik insulin yang berbeza? .5

1.6 Apakah rejim insulin yang ada? 5

BAHAGIAN 2 Ketidakpatuhan terhadap rawatan insulin dan kesannya. ...6

2.1 Apakah maksud ketidakpatuhan terhadap rawatan insulin? ..6

2.2 Apakah kesan ketidakpatuhan terhadap rawatan insulin?......6

BAHAGIAN 3 Punca ketidakpatuhan terhadap rawatan insulin dan cara mengatasinya. 7

3.1 Kesan sampingan insulin 7

3.1.1 Hipoglisemia 7

3.1.2 Peningkatan berat badan 8

3.1.3 Alahan pada insulin 9

3.2 Masalah dengan suntikan insulin 9

3.2.1 Sakit di tempat suntikan 9

3.2.2 Insulin kurang berkesan apabila disuntik pada kawasan sel lemak tidak normal (Lipodystrophy) 9

3.2.3 Pendarahan/lebam di tempat suntikan 10

3.2.4 Malu menyuntik di khalayak ramai 10

3.3 Sikap negatif terhadap insulin 10

3.3.1 Suntikan insulin menyusahkan 10

3.3.2 Suntikan insulin mengganggu waktu kerja 10
3.3.3 Terlupa menyuntik insulin 10
3.3.4 Terlupa membawa insulin ke tempat kerja 10
3.4 Kos rawatan yang tinggi 11
3.5 Persepsi yang salah terhadap insulin 11
3.5.1 Insulin tidak berkesan 11
3.5.2 Paras gula tetap tinggi walaupun menyuntik insulin 12
3.5.3 Tidak perlu suntik insulin semasa berpuasa 12
3.5.4 Tidak boleh membawa insulin menaiki kapal terbang ke luar negara 12
3.6 Mitos tentang insulin 13
BAHAGIAN 4 Memperkasakan penjagaan kendiri diabetes. 14
4.1 Berdisiplin mengawal diabetes 14
4.2 Melakukan pemantauan sendiri paras gula 15
4.3 Melakukan perubahan dos insulin berdasarkan paras gula .. 16
BAHAGIAN 5 Berpuasa dengan selamat walaupun menyuntik insulin. .. 20
5.1 Bagaimanakah mengubahsuai dos insulin ketika berpuasa? 20
5.2 Bilakah saya perlu memantau paras gula ketika berpuasa? . 21
5.3 Bilakah saya perlu berbuka puasa? 21
Rujukan 22
LAMPIRAN A: Jenis pen insulin yang terdapat di pasaran. 23
LAMPIRAN B: Kadar tukaran karbohidrat 24
LAMPIRAN C: Contoh senaman aerobik kadar sederhana 26
LAMPIRAN D: Kawasan tubuh yang sesuai untuk menyuntik insulin 27
LAMPIRAN E: Teknik penggunaan insulin 28
LAMPIRAN F: Pengubahsuaian insulin ketika sakit 34
LAMPIRAN G: Kaedah memeriksa aras gula 35

Pengenalan

Objektif

Tujuan modul ini ialah untuk meningkatkan kefahaman, kesedaran dan kepatuhan pesakit kencing manis terhadap rawatan insulin.
Modul ini mengandungi 5 bahagian iaitu:
Bahagian 1: Diabetes dan kaitannya dengan insulin
Bahagian 2: Ketidakpatuhan terhadap rawatan insulin dan kesannya
Bahagian 3: Punca ketidakpatuhan terhadap rawatan insulin dan cara mengatasi
Bahagian 4: Memperkasakan penjagaan kendiri diabetes.
Bahagian 5: Berpuasa dengan selamat walaupun menyuntik insulin.

Aktiviti

Anda diminta membaca dan memahami modul ini dalam masa 50 minit. Pembahagian masa untuk bahgian-bahagian adalah seperti berikut:
Bahagian 1: 10 minit
Bahagian 2: 5 minit
Bahagian 3: 15 minit
Bahagian 4: 15 minit
Bahagian 5: 5 minit
Selepas 50 minit, anda akan berjumpa doktor selama 10 minit untuk sesi soal jawab.

Penghargaan

Pertamanya, saya ingin mengucapkan ucapan terima kasih yang tak terhingga kepada semua penyelia yang telah membimbing, menyumbangkan idea dan membantu saya menyiapkan modul ini.

Ucapan penghargaan kepada semua panel penilai yang terdiri dari pakar dalam bidang masing-masing yang telah menilai dan memberi komen untuk penambahbaikan kandungan modul.

Terima kasih kepada pesakit yang telah menilai dan memberi komen untuk menjadikan modul ini mudah dibaca dan difahami.

Terima kasih juga kepada jurugrafik, En Khairul Zahari yang telah mengedit gambar, carta, ilustrasi dan kulit modul.

Ucapan penghargaan kepada Universiti Sains Malaysia atas geran penyelidikan 6315140 yang menyumbang kepada penghasilan modul ini.

Terima kasih kepada semua yang terlibat secara tidak lansung dalam penghasilan modul ini.

BAHAGIAN 1 Diabetes dan insulin.

Objektif: Memberi maklumat kepada pesakit diabetes mengenai penyakit diabetes dan kaitannya dengan insulin.
Aktiviti: Membaca dan memahami bahagian 1
Masa: 10 minit

1.1 Definisi diabetes

Diabetes ialah keadaan di mana fungsi pankreas untuk menghasilkan insulin telah rosak/berkurangan atau tisu badan tidak sensitif terhadap insulin. Ini menyebabkan paras gula berlebihan dalam darah. Pesakit mungkin mengalami simptom atau tidak[1]:

- ≥7 mmol/L selepas 8 hingga 10 jam berpuasa dan/atau
- ≥ 11.1 mmol/L selepas 2 jam minum air gula atau
- HbA1c* ≥ 6.3 %

*HbA1c ialah purata gula dalam darah untuk 3 bulan yang melekat pada sel darah merah.

1.2 Jenis-jenis diabetes

Secara umumnya diabetes dikategorikan kepada tiga jenis utama.

**Jenis 1**	**Jenis 2**	**Diabetes ibu mengandung**
• Tiada penghasilan insulin oleh pankreas kerana sel pankreas rosak<br>• Biasanya berlaku dalam kalangan kanak-kanak atau remaja.	• Insulin dihasilkan tidak mencukupi<br>• Sel tubuh tidak sensitif terhadap insulin<br>• Biasanya berlaku dalam kalangan mereka yang berlebihan berat badan	• Diabetes ketika mengandung yang sembuh selepas ibu melahirkan bayi<br>• Ibu berisiko tinggi mendapat diabetes jenis 2 jika tidak mengawal pemakanan

Gambar 1: Jenis-jenis diabetes

1.3 Apakah hubungan antara diabetes dan insulin?

Insulin berfungsi sebagai kunci untuk membuka salur gula di permukaan sel untuk membolehkan gula masuk ke dalam sel untuk menghasilkan tenaga.

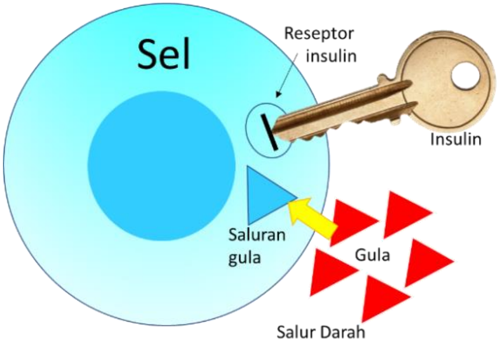

Gambar 2: Insulin sebagai kunci

Jika tiada insulin, gula tidak dapat masuk ke dalam sel dan menyebabkan paras gula tinggi di dalam salur darah.

Pankreas yang normal merembeskan insulin basal sepanjang masa termasuk waktu anda tidur untuk mengekalkan paras gula normal dalam darah. Pankreas yang normal juga bertindak balas mengeluarkan insulin semasa makan (insulin prandial). Jadi paras gula akan menurun dalam tempoh 2 jam selepas makan.

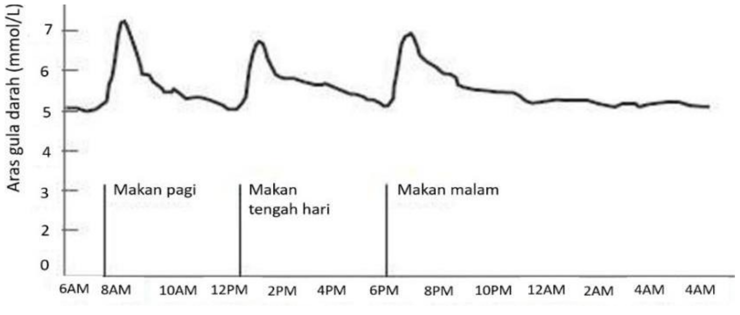

Carta 1: Paras gula normal

Manakala, bagi anda, pankreas anda tidak lagi mengeluarkan insulin yang mencukupi atau sel tubuh tidak sensitif terhadap insulin.

- Kekurangan insulin **basal** menyebabkan paras gula dalam darah anda tinggi **sejurus** anda **bangun tidur**.
- Kekurangan insulin **prandial** menyebabkan paras gula dalam darah anda berterusan tinggi (garisan merah) **selepas makan.**

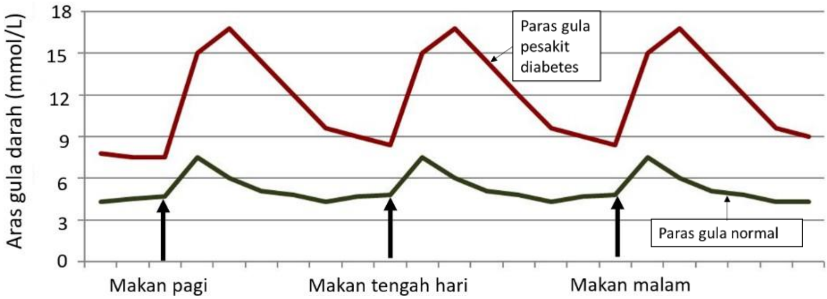

Carta 2: Paras gula normal dan paras gula pesakit diabetes

1.4 Jenis- jenis insulin

Secara umumnya, insulin terbahagi kepada 3 jenis iaitu;

- **Basal:** menurunkan gula untuk jangka panjang (16-24 jam)
- **Prandial:** menurunkan gula sejurus selepas makan
- **Kombinasi**: campuran basal dan prandial atau insulin pra-campuran

Lampiran A menunjukkan jenis-jenis pen insulin yang terdapat di pasaran.

Selepas disuntik ke dalam badan, insulin memerlukan selang waktu yang berbeza untuk mula bertindak menurunkan gula. Tempoh tindakannya juga berbeza mengikut jenis insulin. Oleh itu, anda perlu merancang waktu suntikan sebelum makan berdasarkan jenis insulin yang digunakan seperti diringkaskan dalam jadual di bawah:

Jadual 1: Jenis-jenis insulin

Jenis insulin	Subjenis	Nama insulin	Mula bertindak	Tempoh bertindak (jam)	Masa suntikan sebelum makan (minit)
**Insulin Basal**	Sederhana	Insulatard®	1.5 jam	18-23	Sebelum tidur/ pada waktu yang sama setiap hari
		Humulin N®	1 jam	16-18	
	Panjang	Lantus® (Glargine)	2-4 jam	20-24	
		Levemir® (Determir)	1 jam	17-23	
**Insulin Prandial**	Sangat pantas (Rapid)	Novorapid® (Aspart)	10-20 min	3-5	0-15 / sejurus selepas makan
		Humalog® (Lispro)	0- 15 min	3.5- 4.5	
		Apidra® (Glulisine)	5- 15 min	3.5	
	Pantas (Fast)	Actrapid®	30 min	8	30
		Humulin R®	30 min	6-8	
**Pra-campuran**	Sangat pantas (Rapid)	NovoMix® 30	10-20 min	16-18	0-15
		Humalog Max® 25/75	0-15 min	16-18	
	Pantas (Fast)	Mixtard® 30	30 min	18-23	30
		Humulin® 30/70	30 min	18-23	

Carta di bawah menunjukkan waktu mula dan tempoh masa insulin bertindak.

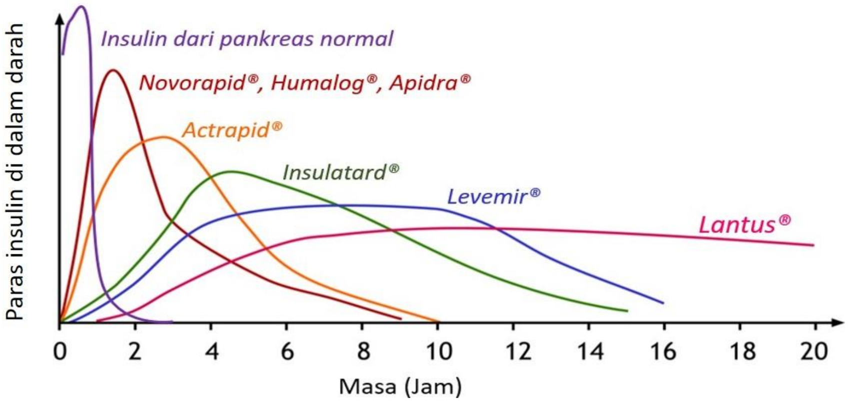

Carta 3: Waktu mula dan tempoh tindakan insulin insulin

1.5 Mengapa pesakit diabetes menyuntik insulin yang berbeza?

Pesakit diabetes memerlukan insulin yang berbeza berdasarkan paras gula yang tidak mencapai sasaran. Ada pesakit yang memerlukan insulin basal sahaja. Ada pesakit yang memerlukan insulin basal dan prandial. Anda perlu mendapatkan nasihat doktor untuk rawatan insulin kerana anda mungkin memerlukan jenis insulin dan **rejim*** insulin yang berbeza dengan pesakit diabetes lain.

* Rejim bermaksud jenis dan bilangan suntikan insulin yang digunakan

1.6 Apakah rejim insulin yang ada?

- Suntikan basal sahaja
- Suntikan basal bersama 1 suntikan prandial
- Suntikan basal bersama 2 suntikan prandial
- Suntikan basal bersama 3 suntikan prandial (basal bolus)
- Suntikan insulin pra-campuran 2 kali
- Suntikan insulin pra-campuran 3 kali

BAHAGIAN 2 Ketidakpatuhan terhadap rawatan insulin dan kesannya.

Objektif: Meningkatkan pengetahuan dan kefahaman pesakit diabetes tentang ketidakpatuhan terhadap rawatan insulin dan kesannya.
Aktiviti: Membaca dan memahami bahagian 2.
Masa: 5 minit.

2.1 Apakah maksud ketidakpatuhan terhadap rawatan insulin?

Ketidakpatuhan terhadap rawatan insulin bermaksud anda tidak mengambil suntikan, atau mengubah bilangan suntikan atau mengubah dos insulin yang digunakan tanpa nasihat atau persetujuan dengan doktor [2].

2.2 Apakah kesan ketidakpatuhan terhadap rawatan insulin?

Apabila anda tidak patuh menyuntik insulin, gula yang terhasil daripada makanan yang anda makan tidak dapat masuk ke dalam sel. Gula akan kekal di dalam salur darah menyebabkan paras gula di dalam darah tinggi. Kesannya, anda akan kerap kencing, dahaga dan susut berat badan. Gula yang berlebihan akan merosakkan salur darah, sel buah pinggang dan sel saraf menyebabkan komplikasi jangka panjang seperti:

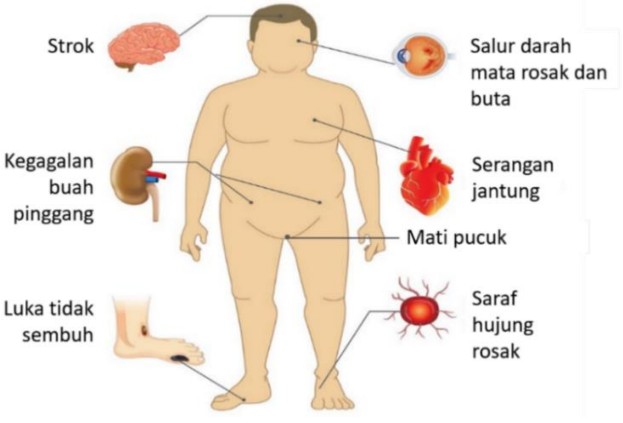

Gambar 3: Kesan ketidakpatuhan terhadap rawatan insulin

BAHAGIAN 3 Punca ketidakpatuhan terhadap rawatan insulin dan cara mengatasinya.

Objektif:
Meningkatkan pengetahuan dan kefahaman pesakit diabetes tentang punca ketidakpatuhan menyuntik insulin dan cara mengatasinya.
Aktiviti: Membaca dan memahami bahagian 3.
Masa: 15 minit.

Terdapat banyak sebab pesakit diabetes tidak patuh terhadap rawatan insulin. Antara punca ketidakpatuhan terhadap rawatan insulin ialah:

3.1 Kesan sampingan insulin

3.1.1 Hipoglisemia

Hipoglisemia ialah keadaan paras gula dalam darah terlalu rendah[3] iaitu kurang dari 4.0 mmol/L dan pesakit mungkin mengalami tanda seperti lapar, letih, berdebar, berpeluh, pening, sakit kepala, loya, menggigil, cemas, mudah marah, mimpi buruk, pitam/ pengsan.[4] Ada segelintir pesakit pernah mengalami hipoglisemia dan takut untuk meneruskan suntikan/ mengurangkan dos insulin tanpa melakukan pemantauan paras gula.

Jadual 2: Punca berlaku hipoglisemia dan cara mengatasi

Punca berlaku hipoglisemia	Cara mengatasinya
Makan terlalu sedikit/ tidak makan dan menyuntik insulin	Makan ikut jadual dan makan dalam kuantiti yang sama mengikut waktu makan tertentu. Minuman Nutrisi seperti Nutren Diabetik/ Glucerna boleh diambil untuk menggantikan makanan ketika kesuntukan masa/ kurang selera makan (kandungan karbohidrat yang tetap)
Lewat makan selepas menyuntik insulin prandial	Segera makan apabila mencapai selang waktu insulin prandial bertindak.

Dos insulin berlebihan	Pantau paras gula dan kurangkan dos insulin jika berlaku hipoglisemia.
Senaman yang berlebihan	Ambil 15-30g karbohidrat jika paras gula kurang dari 5.6 mmol/L sebelum bersenam[5]
Pengambilan alkohol	Berhenti mengambil minuman beralkohol

Jika berlaku **hipoglisemia**, anda perlu ambil makanan/ minuman manis dalam kadar tertentu berdasarkan paras gula[3].

- Paras gula 3.3 -3.9 mmol/L: 3 sudu besar gula putih **atau** 2 biji gula-gula **atau** setengah cawan (120 ml) jus buah-buahan bergula.
- Paras gula 2.5 – 3.2 mmol/L: 4 sudu besar gula putih/3 biji gula-gula **atau** ¾ cawan (180ml) jus buah-buahan bergula.
- Paras gula <2.5 mmol/L: 6 sudu besar gula putih **atau** 5 biji gula-gula **atau** 1 cawan (240ml) jus buah-buahan bergula.
- **Pesakit tidak sedarkan diri**: Segera bawa pesakit ke klinik kesihatan/ hospital untuk rawatan air gula ke dalam salur darah.

3.1.2 Peningkatan berat badan

Suntikan insulin menyebabkan paras gula turun, menyebabkan berasa lapar dan mengambil makanan antara waktu makan. Maka, pesakit mengambil kalori melebihi keperluan tubuh.

Punca lain peningkatan berat badan adalah disebabkan oleh:

- Kawalan gula yang baik mengurangkan kehilangan gula di dalam air kencing. Maka lebih banyak kalori tersimpan di dalam tubuh.
- Insulin meningkatkan jisim badan tanpa lemak melalui sifat anaboliknya.
- Insulin boleh menyebabkan air dan garam tersimpan di dalam tubuh.
- Kurang bersenam.

Elakkan kenaikan berat badan dengan cara:

- Mengenal pasti punca berlaku hipoglisemia dan mengambil

langkah pencegahan.

- Menghadkan pengambilan karbohidrat sebanyak 11-12 tukaran sehari (iaitu 2 tukaran buah, 1 tukaran susu, 1 tukaran gula dan 7-8 tukaran bijirin). (Rujuk **Lampiran B**)
- Melakukan senaman aerobik intensiti sederhana selama 150 minit seminggu. (Rujuk **Lampiran C**).

3.1.3 Alahan pada insulin

Alahan kepada insulin jarang berlaku. Selepas insulin yang dimurnikan telah diperkenalkan, hanya 1 dalam 1000 orang mengalami alahan. Tanda-tanda alahan ialah kemerahan dan gatal pada kawasan suntikan. Jika kemerahan dan gatal merebak ke seluruh tubuh, atau bibir bengkak dan sukar bernafas, anda perlu menghentikan suntikan dan mendapatkan rawatan di hospital.

3.2 Masalah dengan suntikan insulin

3.2.1 Sakit di tempat suntikan

Jadual 3: Punca sakit di tempat suntikan dan cara mengatasi

Punca sakit di tempat suntikan	Cara mengatasinya
Jarum yang tumpul kerana digunakan berulang kali	Setiap jarum digunakan sekali sahaja*
Menyuntik di kawasan yang sama berulang kali	Menyuntik insulin di tempat berbeza dengan teknik yang betul (Rujuk **Lampiran D & E**)
Menyuntik pada kawasan yang berparut	Elakkan menyuntik insulin di tempat berparut

*Jika anda mengalami kekangan kewangan, maksimum penggunaan jarum tiga (3) kali sahaja.

3.2.2 Insulin kurang berkesan apabila disuntik pada kawasan sel lemak tidak normal (Lipodystrophy)

Berlebihan sel lemak (lipohypertrophy) atau berkurangan sel lemak

(lipoatrophy) di bawah kulit boleh berlaku jika menyuntik di tempat yang sama berulang kali. Hal ini menyebabkan insulin kurang berkesan. Suntik insulin di tempat berbeza dengan teknik yang betul.

3.2.3 Pendarahan/lebam di tempat suntikan

Pendarahan/ lebam berlaku jika anda tersuntik kapilari darah di bawah kulit. Untuk mengelakkannya, suntik insulin dengan teknik dan saiz jarum yang betul.

3.2.4 Malu menyuntik di khalayak ramai

Kawasan suntikan adalah aurat bagi pesakit wanita Muslim.
Lelaki yang sopan juga tidak akan mendedahkan kawasan tubuh di hadapan khalayak.
Anda boleh menyuntik insulin di dalam kenderaan atau cari tempat tertutup yang sesuai.

3.3 Sikap negatif terhadap insulin

3.3.1 Suntikan insulin menyusahkan

Ada pesakit menganggap menyuntik insulin menyusahkan. Jika anda faham suntikan insulin itu perlu untuk anda sihat, anda akan berusaha supaya ia jadi mudah.

3.3.2 Suntikan insulin mengganggu waktu kerja

Suntikan insulin boleh menyebabkan gangguan pada waktu kerja. Sekiranya insulin mengganggu waktu kerja, anda perlu berjumpa doktor untuk menukar rejim insulin yang sesuai.

3.3.3 Terlupa menyuntik insulin

- Letakkan peringatan di telefon, atau
- Kunci jam, atau

Minta ahli keluarga untuk mengingatkan anda, dan

- Ingatkan diri sendiri yang anda perlukan sesuatu sebelum makan untuk memastikan anda sihat.

3.3.4 Terlupa membawa insulin ke tempat kerja

Untuk mengelakkan anda terlupa membawa pen insulin ke tempat

kerja:

- Letakkan pen insulin di dalam beg kerja sejurus selepas suntikan terakhir di rumah, atau
- Khaskan satu pen insulin yang digunakan di tempat kerja (jika anda mampu).

3.4 Kos rawatan yang tinggi

Anda perlu memperuntukkan sejumlah wang untuk membeli jarum suntikan insulin, jarum dan strip pemantauan paras gula. Sertailah kumpulan sokongan pesakit diabetes. Kebanyakan kumpulan sokongan pesakit diabetes menjual jarum dan strip pada harga diskaun sebagai ahli. Contoh kumpulan sokongan yang boleh anda sertai dan maklumat persatuan adalah seperti berikut:

Jadual 4: Kumpulan sokongan pesakit diabetes

	Nama Persatuan	Alamat	No telefon
1	Persatuan Diabetes Malaysia cawangan USM	Klinik Pakar Perubatan, Hospital USM	097673564
2	Persatuan Diabetes Malaysia cawangan HRPZ II	Kompleks rawatan harian HRPZ II	09- 745 2000 dan minta operator sambungkan ke Pusat Sumber Diabetes
3	Persatuan diabetes di hospital-hospital daerah dan klinik-klinik kesihatan	Sila berhubung dengan jururawat atau staf yang terlibat dengan penjagaan diabetes di fasiliti kesihatan tersebut	

3.5 Persepsi yang salah terhadap insulin

3.5.1 Insulin tidak berkesan

Ada segelintir pesakit yang menganggap insulin tidak berkesan untuk menurunkan paras gula. Anda perlu memantau paras gula dan **menambah** dos insulin sehingga paras gula anda mencapai sasaran.

3.5.2 Paras gula tetap tinggi walaupun menyuntik insulin

Jadual 5: Punca gula tetap tinggi walaupun menyuntik insulin dan cara mengatasi

Punca gula tetap tinggi	Cara mengatasinya
Tubuh anda tidak sensitif terhadap insulin	Tingkatkan aktiviti fizikal untuk menambahkan sensitiviti insulin
Dos insulin tidak mencukupi	Pantau paras gula dan tambahkan dos insulin sehingga paras gula mencapai sasaran
Anda sakit/ stress dan tubuh anda mengeluarkan hormon yang meningkatkan paras gula	Pantau paras gula semasa sakit dan ubah dos insulin berdasarkan paras gula anda (Rujuk **Lampiran F**)
Insulin telah rosak kerana terdedah kepada panas melampau	Simpan pen insulin yang belum dibuka pada suhu 2-8°C* dan pen yang telah dibuka pada suhu 15-27°C. Jangan biarkan pen insulin terdedah pada suhu melampau.
Insulin ternyahaktif kerana telah beku dan cair semula	Jangan simpan pen insulin di tempat beku
Melepasi tarikh luput	Periksa tarikh luput sebelum menyuntik insulin

* Simpan di bahagian dalam peti sejuk berdekatan penyejuk. Jangan simpan di pintu peti sejuk kerana suhunya tidak tetap.

3.5.3 Tidak perlu suntik insulin semasa berpuasa

Hanya insulin yang disuntik sebelum makan tengahari sahaja yang tidak perlu disuntik ketika berpuasa. Suntikan lain perlu diteruskan dengan pengubahsuaian dos yang sesuai (Sila rujuk bahagian 5).

3.5.4 Tidak boleh membawa insulin menaiki kapal terbang ke luar negara

Sebenarnya, anda boleh membawa insulin semasa ke luar negara.

Untuk memastikan insulin anda melepasi tapisan keselamatan di lapangan terbang, bawa bekalan insulin yang mencukupi untuk tempoh lawatan di dalam bungkusan asal dan dapatkan surat doktor yang mengesahkan anda membawa insulin untuk rawatan diabetes.

3.6 Mitos tentang insulin

Jadual 6: Mitos tentang insulin

Mitos	Fakta sebenar
**Insulin diperbuat dari sumber khinzir**	Kini, tiada lagi insulin diperbuat dari sumber khinzir. DNA* insulin manusia disuntik ke dalam DNA bakteria dan dibiakkan di dalam tangki fermentasi. Apabila bakteria telah membiak, insulin dituai dan dimurnikan dan disediakan untuk kegunaan perubatan[6].
**Insulin akan merosakkan buah pinggang**	Insulin tidak merosakkan buah pinggang. Punca utama kerosakan buah pinggang disebabkan oleh paras gula yang tinggi dan berterusan dalam jangka masa panjang.
**Bila mula menggunakan insulin, diabetes saya tidak boleh diubati lagi.**	Menyuntik insulin bukan bererti anda telah sampai ke peringkat diabetes paling teruk dan tidak boleh diubati lagi.<br>Ketika doktor mendiagnos anda menghidap diabetes, 50% dari sel pankreas anda telah rosak dan tidak menghasilkan insulin[7]. Fungsi pankreas akan terus berkurangan dari masa ke masa. Selepas 5-10 tahun menghidap diabetes, kebanyakan pesakit tidak lagi menghasilkan insulin dan perlu menyuntik insulin untuk mengawal paras gula.<br>Ramai pesakit yang dapat mencapai paras gula sasaran dengan menyuntik insulin. Ini dapat menghindari komplikasi diabetes berbanding pesakit yang gagal mengawal paras gula dengan ubat makan dan enggan menyuntik insulin.

*DNA - deoxyribonucleic acid (struktur utama dalam kromosom yang membawa maklumat genetik)

BAHAGIAN 4 Memperkasakan penjagaan kendiri diabetes.

Objektif:
1. Meningkatkan pengetahuan dan kefahaman tentang sasaran kawalan gula, tekanan darah, kolesterol dan berat badan ideal.
2. Meningkatkan motivasi pesakit diabetes untuk memantau paras gula dan mengubah dos insulin.
Aktiviti: Membaca dan memahami bahagian 4.
Masa: 10 minit.

4.1 Berdisiplin mengawal diabetes

Anda tidak boleh mengharapkan doktor untuk mengawal diabetes anda. Anda bertanggungjawab menjaga diri anda sendiri!
Jika anda tidak mahu menghabiskan hujung usia dengan komplikasi diabetes seperti dialisis, strok, buta, cacat anggota dan sebagainya, anda sendirilah yang perlu melakukannya.
Bagaimana melakukannya?

- Berdisiplin dengan waktu makan anda
- Pilih makanan anda
- Bersenam 150 minit seminggu
- Memantau sendiri paras gula dan mengubah dos insulin sehingga mencapai paras gula sasaran
- Mengambil tahu sasaran kawalan dan berusaha mencapainya

Jadual 7: Sasaran kawalan[1]

	Parameter	Bacaan sasaran
A	HbA1c (purata gula 3 bulan)	
	Baru disahkan mengidap diabetes, tiada komplikasi, jangka hayat panjang, kurang risiko hipoglisemia	6.0- 6.5%
	Yang lain-lain	6.6 -7.0 %
	Ada komplikasi jantung, buah pinggang/ hati, jangka hayat pendek, berisiko hipoglisemia	7.1 – 8.0 %

**B**	Blood pressure (tekanan darah)	<135/75 mmHg
**C**	Kolesterol (dalam unit mmol/L)	HDL-C > 1.0 bagi lelaki<br>> 1.2 bagi wanita<br>LDL-C < 2.6<br>Triglyceride <1.7
	Berat badan	Jika berat berlebihan, sasarkan penurunan berat badan sebanyak 5-10% dalam masa 6 bulan

4.2 Melakukan pemantauan sendiri paras gula

Pemantauan sendiri paras gula bermaksud anda melakukan pemeriksaan paras gula sendiri di rumah dengan menggunakan "glucometer" (alat pengukur paras gula) tanpa kehadiran doktor atau jururawat. Ia dilakukan untuk mengetahui perubahan paras gula dalam darah berdasarkan pemakanan, aktiviti yang dilakukan dan dos insulin yang digunakan. Kajian menunjukkan, pesakit yang melakukannya dapat mengawal paras gula dengan lebih baik berbanding pesakit yang tidak melakukannya[8].

Secara ideal, anda digalakkan memantau sendiri paras gula setiap kali sebelum menyuntik insulin. Paras gula sasaran adalah seperti berikut:

Jadual 8: Paras gula sasaran

Paras gula berpuasa	4.4 – 7.0 mmol/L
Paras gula 2 jam selepas makan	4.4 – 8.5 mmol/L

Sila rujuk **Lampiran G** untuk kaedah memeriksa paras gula.

4.3 Melakukan perubahan dos insulin berdasarkan paras gula

Secara ideal, anda digalakkan memantau sendiri paras gula setiap kali sebelum menyuntik insulin.

- Sekiranya paras gula anda melebihi sasaran sebanyak tiga kali berturut-turut, anda perlu menambah dos insulin sebanyak 2 unit. Tambah 2 unit **HANYA** jika 3 bacaan melebihi sasaran.
- Jika paras gula kurang dari paras sasaran atau mengalami hipoglisemia, anda perlu mengurangkan dos insulin sebanyak 2 unit.
- Kekalkan dos insulin jika paras gula anda dalam julat sasaran.

Sebagai contoh, jika anda menyuntik insulin **basal** sebelum tidur sebanyak 12 unit, anda perlu memantau paras gula sebelum sarapan pagi. Jika bacaannya melebihi 7.0 mmol/L sebanyak 3 kali, anda perlu menaikkan dos insulin kepada 14 unit.

Jadual 9: Contoh kaedah mengubah dos insulin untuk rejim basal

Tarikh*	Paras gula sebelum sarapan pagi (mmol/L)	Dos insulin sebelum tidur (unit)
1.6.20		12
2.6.20	7.2	12
3.6.20	7.8	12
4.6.20	8.5	**14** (+2 unit)

**Anda tidak semestinya memeriksa paras gula setiap hari. Contohnya, anda boleh melakukannya 3 kali seminggu. Jika ketiga-tiga bacaan melebihi paras normal, tambah dos insulin setiap minggu sehingga paras gula mencapai paras sasaran.*

Jika anda menyuntik insulin **pra-campuran 2 kali sehari** (sebelum sarapan pagi dan sebelum makan malam), anda perlu memantau paras gula dua kali:

- Jika paras gula melebihi 7.0 mmol/L sebelum sarapan pagi sebanyak 3 kali, tambahkan 2 unit dos insulin sebelum makan malam
- Jika paras gula melebihi 7.0 mmol/L sebelum makan malam sebanyak 3 kali, tambahkan 2 unit dos insulin sebelum makan pagi.

Jadual 10: Contoh kaedah mengubah dos insulin untuk rejim pra-campuran 2 kali sehari

Tarikh	Paras gula sebelum sarapan pagi (mmol/L)	Dos insulin sebelum sarapan pagi (unit)	Paras gula sebelum makan malam (mmol/L)	Dos insulin sebelum makan malam (unit)
1.6.20				12
2.6.20	7.2			12
3.6.20	7.8			12
4.6.20	8.5			**14** (+2 unit)
5.6.20		12	8.7	
6.6.20		12	7.5	
7.6.20		12	7.2	
8.6.20		**14** (+2 unit)		

Jika anda menyuntik insulin **basal bolus (4 kali sehari)**, anda perlu memantau paras gula 4 kali sehari:

- Jika paras gula melebihi 7.0 mmol/L sebelum sarapan pagi sebanyak 3 kali, tambahkan 2 unit dos insulin sebelum tidur.
- Jika paras gula melebihi 7.0 mmol/L sebelum makan tengah hari sebanyak 3 kali, tambahkan 2 unit dos insulin sebelum sarapan pagi.
- Jika paras gula melebihi 7.0 mmol/L sebelum makan malam sebanyak 3 kali, tambahkan 2 unit dos insulin sebelum makan tengah hari.
- Jika paras gula melebihi 7.0 mmol/L sebelum tidur sebanyak 3 kali, tambahkan 2 unit dos insulin sebelum makan malam.

Jadual 11: Contoh kaedah mengubah dos insulin bagi regim basal bolus

Tarikh	Paras gula sebelum sarapan pagi (mmol/L)	Dos insulin sebelum sarapan pagi	Paras gula sebelum makan tengahri (mmol/L)	Dos insulin sebelum makan tengah hari (unit)	Paras gula sebelum makan malam (mmol/L)	Dos insulin sebelum makan malam (unit)	Paras gula sebelum tidur (mmol/L)	Dos insulin sebelum tidur
1.6.20								12
2.6.20	7.2							12
3.6.20	7.8							12
4.6.20	8.5							**14** (+2 unit)
5.6.20		12	8.7					
6.6.20		12	7.5					
7.6.20		12	7.2					
8.6.20		**14** (+2 unit)		12	7.2			
9.6.20				12	7.8			
10.6.20				12	8.5			
11.6.20				**14** (+2 unit)		12	8.7	
12.6.20						12	7.5	
13.6.20						12	7.2	
13.6.20						**14** (+2 unit		

Jika anda menyuntik insulin prandial sangat pantas seperti Novorapid®, Humalog® atau Apidra®, anda boleh memeriksa paras gula 2 jam selepas makan. Jika paras gula melebihi 8.5 mmol/L 2 jam selepas makan pagi sebanyak 3 kali bacaan, tambah 2 unit dos insulin sebelum sarapan pagi atau tengah hari atau makan malam pada keesokan hari.

Jadual 12: Contoh kaedah mengubah dos insulin bagi insulin prandial sangat pantas

Tarikh	Dos insulin sebelum makan pagi (unit)	Paras gula 2 jam selepas makan pagi (mmol/L)	Dos insulin sebelum makan tengah hari (unit)	Paras gula 2 jam selepas makan tengahari (mmol/L)	Dos insulin sebelum makan malam (unit)	Paras gula 2 jam selepas makan malam (mmol/L)
1.6.20	12	8.7				
2.6.20	12	8.9				
3.6.20	12	8.6				
4.6.20	**14**		12	8.7		
5.6.20			12	8.9		
6.6.20	(+2 unit)		12	8.6		
7.6.20			**14**		12	8.9
8.6.20		(+2 unit)			12	8.5
9.6.20					12	8.5
10.6.20					**14**	

(+2 unit)

BAHAGIAN 5 Berpuasa dengan selamat walaupun menyuntik insulin.

Objektif: Meningkatkan pengetahuan pesakit diabetes tentang cara-cara untuk berpuasa secara selamat.
Aktiviti: Membaca dan memahami bahagian 5.
Masa: 5 minit.
Semasa berpuasa, pesakit diabetes mempunyai risiko yang tinggi untuk mendapat[9]:

- Hipoglisemia (gula rendah dalam darah),
- Hiperglisemia (gula berlebihan dalam darah),
- Dehidrasi (air berkurangan dalam badan),
- ketoacidosis (asid berlebihan dalam darah), dan
- trombosis (darah beku dalam salur darah).

Kesemua risiko ini dapat dikurangkan dengan pengetahuan, pemantauan paras gula semasa berpuasa dan perubahan dos insulin.

5.1 Bagaimanakah mengubahsuai dos insulin ketika berpuasa?

Jika anda menyuntik insulin **basal** sebelum tidur, teruskan menyuntik seperti biasa. Anda mungkin perlu mengurangkan dos jika berlaku hipoglisemia semasa berpuasa.
Jika anda menggunakan insulin **pra-campuran dua kali sehari**:

- Dos makan pagi digunakan sebelum berbuka.
- Dos makan malam digunakan sebelum sahur. Kurangkan dos sebelum sahur sebanyak 20-50%. Contohnya, jika anda menyuntik insulin sebanyak 20 unit, kurangkan insulin sebanyak 4 ke 10 unit.

Jika anda menggunakan insulin **pra-campuran tiga kali** sehari:

- Dos sarapan pagi digunakan sebelum berbuka
- Meninggalkan suntikan insulin untuk waktu tengah hari.
- Dos makan malam digunakan sebelum sahur. Kurangkan dos sebelum sahur sebanyak 20-50%.

Jika anda menyuntik insulin **basal bolus** (4 kali) sehari:

- Sebelum sahur, suntik insulin mengikut dos sebelum makan malam, kurangkan sebanyak 20-50%. Kurangkan dos jika berlaku hipoglisemia.
- Meninggalkan suntikan insulin untuk waktu tengah hari.
- Sebelum berbuka, suntik insulin mengikut dos insulin sebelum sarapan pagi.
- Dos basal insulin diambil sebelum tidur atau selepas berbuka.

5.2 Bilakah saya perlu memantau paras gula ketika berpuasa?

Anda digalakkan memantau paras gula pada waktu berikut, terutamanya pada hari-hari awal berpuasa. Anda boleh memilih untuk memantau 2 atau 3 bacaan paras gula seperti di bawah:

- Sebelum sahur dan 2 jam selepas sahur
- Sebelum berbuka dan 2 jam selepas berbuka
- Ketika mengalami sebarang simptom hipoglisemia
- Sekiranya paras gula anda telah stabil, anda boleh mengurangkan kekerapan pemantauan paras gula mengikut regim insulin anda.

5.3 Bilakah saya perlu berbuka puasa?

- Paras gula < 3.3 mmol/L semasa berpuasa
- Paras gula <3.9 mmol/L dalam beberapa jam pertama berpuasa
- Paras gula > 16.7 mmol.L
- Mengalami tanda-tanda hipoglisemia (dan tiada alat pengukur paras gula)
- Gejala kurang air yang teruk seperti keliru atau koma

Rujukan

1. Ministry of Health Malaysia. *Clinical Practice Guidelines Management of Type 2 Diabetes Mellitus*.; 2015. doi:10.1088/1751-8113/44/8/085201
2. Blackburn DF, Swidrovich J, Lemstra M. Non-adherence in type 2 diabetes: Practical considerations for interpreting the literature. *Patient Prefer Adherence*. 2013;7:183-189. doi:10.2147/PPA.S30613
3. Zanariah H, Nurain MN, Md M. Practical Guide to Insulin Therapy in Type 2 Diabetes. *Minist Heal Malaysia*. 2011:22-30. https://www.researchgate.net/publication/278406704_Practical_Guide_To_Insulin_Therapy_in_Type_2_Diabetes.
4. diabetes.co.uk. Diabetes and Hypoglycemia. https://www.diabetes.co.uk/Diabetes-and-Hypoglycaemia.html. Published 2019. Accessed May 19, 2020.
5. Blood Sugar and Exercise. https://www.diabetes.org/fitness/get-and-stay-fit/getting-started-safely/blood-glucose-and-exercise. Accessed May 19, 2020.
6. Keen H, Pickup JC, Bilous RW, et al. Human Insulin Produced By Recombinant Dna Technology: Safety and Hypoglycæmic Potency in Healthy Men. *Lancet*. 1980;316(8191):398-401. doi:10.1016/S0140-6736(80)90443-2
7. Fonseca VA. Defining and characterizing the progression of type 2 diabetes. *Diabetes Care*. 2009;32 Suppl 2(suppl_2):S151-6. doi:10.2337/dc09-S301
8. McAndrew L, Schneider SH, Burns E, Leventhal H. Does patient blood glucose monitoring improve diabetes control?: A systematic review of the literature. *Diabetes Educ*. 2007;33(6):991-1011. doi:10.1177/0145721707309807
9. Hussein Z, Hallaj Rahmatullah I, Mohamad M, Aziz NA, Yusoff Azmi Merican NS. Practical Guide to Diabetes Management in Ramadan. 2015:10.

LAMPIRAN A: Jenis pen insulin yang terdapat di pasaran.

Jenis Pen Insulin	Nama Insulin
KwikPen® 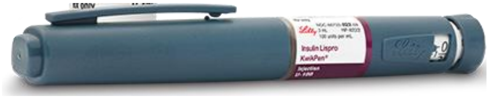	Lispro®
Humalog **HumaPen® (Ergo)** 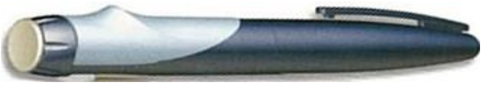	Humalog mix 25<br>Humulin R®<br>Humulin N®<br>Humulin® 30/70<br>Humulin ® 50/50
**Novopen® 3**<br>**Novopen® 4** 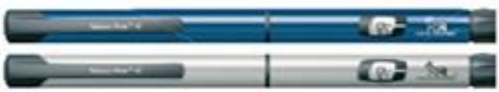	Actrapid®<br>Insulatard®<br>Mixtard® 30/70<br>Novorapid®
**Novolet®** 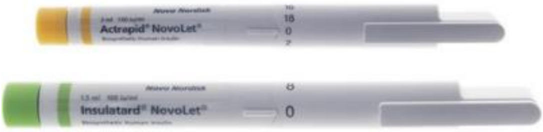	Actrapid®<br>Insulatard®<br>Mixtard® 30/70
**Flexpen®** 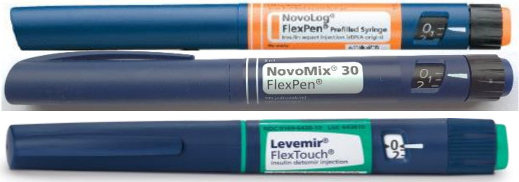	Novorapid®<br>Novomix® 30<br>Levermir®
**SoloSTAR®** 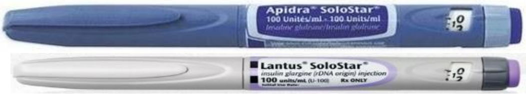	Apidra®<br>Lantus®
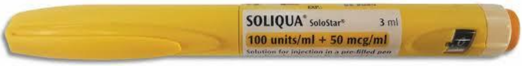	Soliqua®<br>Glargine+<br>lixisenatide

LAMPIRAN B: Kadar tukaran karbohidrat

Hadkan makan 11-12 tukaran karbohidrat dalam sehari, iaitu:

- 2 tukaran buah
- 1 tukaran gula
- 1 tukaran susu, dan
- 7 tukaran bijirin.

Setiap gambar menunjukkan 1 tukaran karbohidrat.

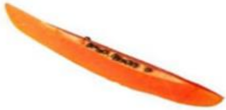
1 hiris betik/ nenas/ tembikai

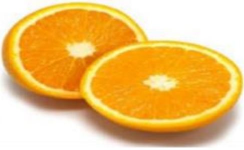
1 biji oren/ pir/ lai/ kiwi

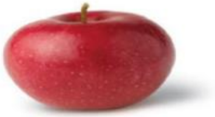
1 biji epal

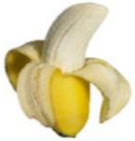
1 biji pisang kecil

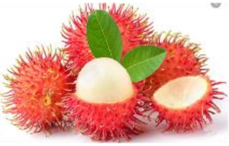
5 biji rambutan

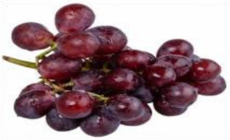
8 biji anggur

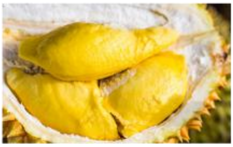
3 ulas durian

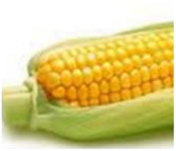
½ tongkol jagung

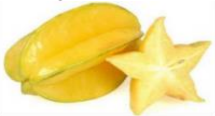
1 biji belimbing

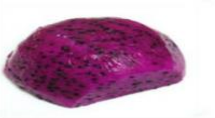
½ biji buah naga

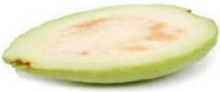
½ biji jambu batu

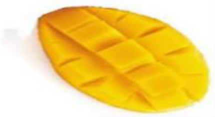
½ biji pauh

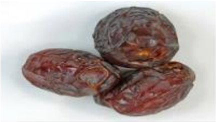
3 biji kurma/ prun

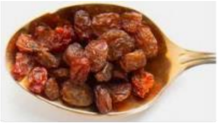
1 sudu makan kismis

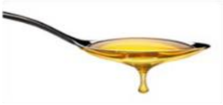
1 sudu makan madu

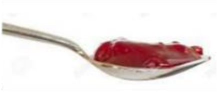
1 sudu makan jem

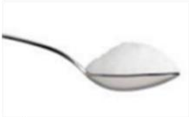
½ sudu makan gula

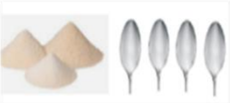
4 sudu susu tepung

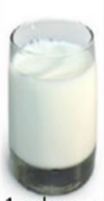
1 gelas susu

Hadkan makan 7-8 tukaran bijirin dalam sehari.

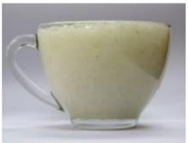
1 cawan bubur

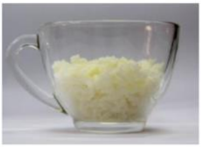
½ cawan nasi

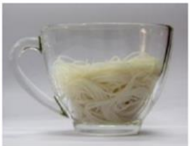
½ cawan bihun

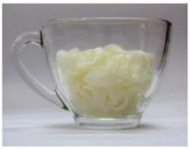
½ cawan kuey teow

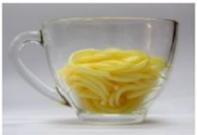
½ cawan mee kuning

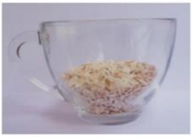
½ cawan oat

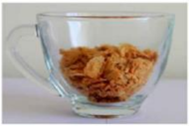
½ cawan bijirin

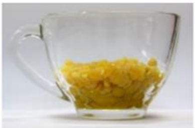
½ cawan pasta

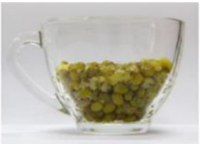
½ cawan kacang hijau

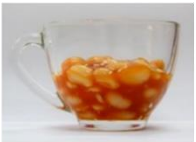
½ cawan kacang panggang

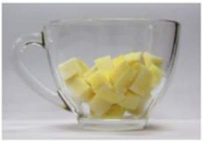
½ cawan ubi kayu

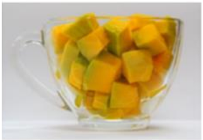
1 cawan labu

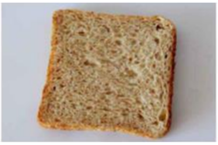
1 keping roti

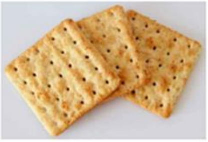
3 keping biskut

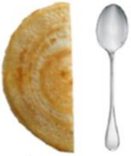
½ keping tosai

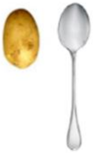
1 ubi kentang kecil

LAMPIRAN C: Contoh senaman aerobik kadar sederhana

Anda digalakkan melakukan senaman aerobik kadar sederhana selama 150 minit seminggu.

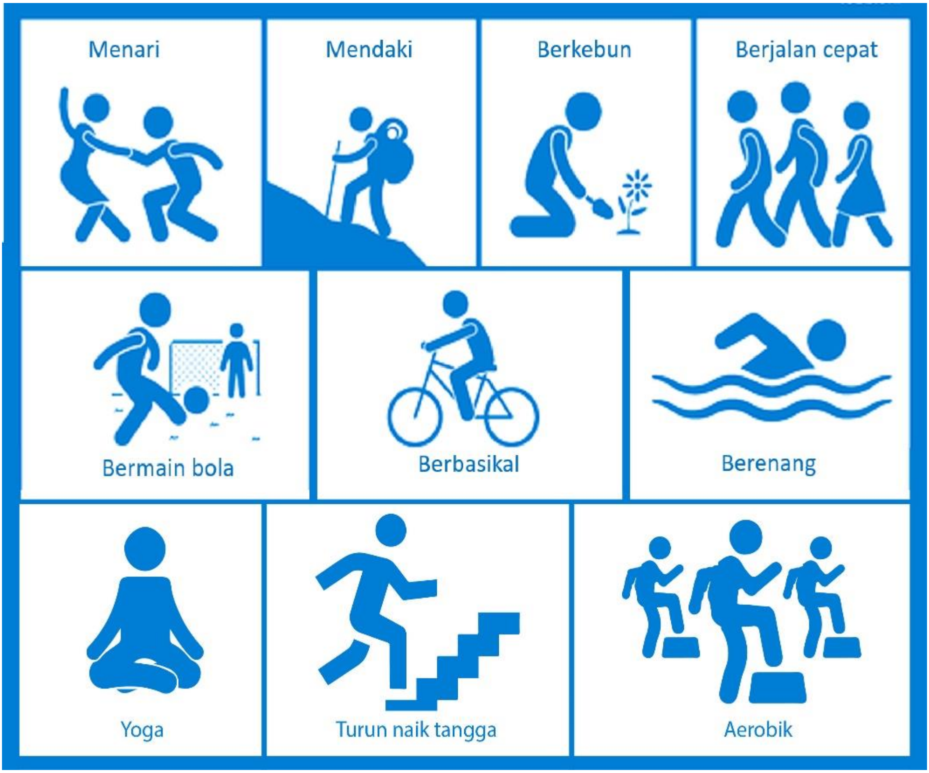

LAMPIRAN D: Kawasan tubuh yang sesuai untuk menyuntik insulin

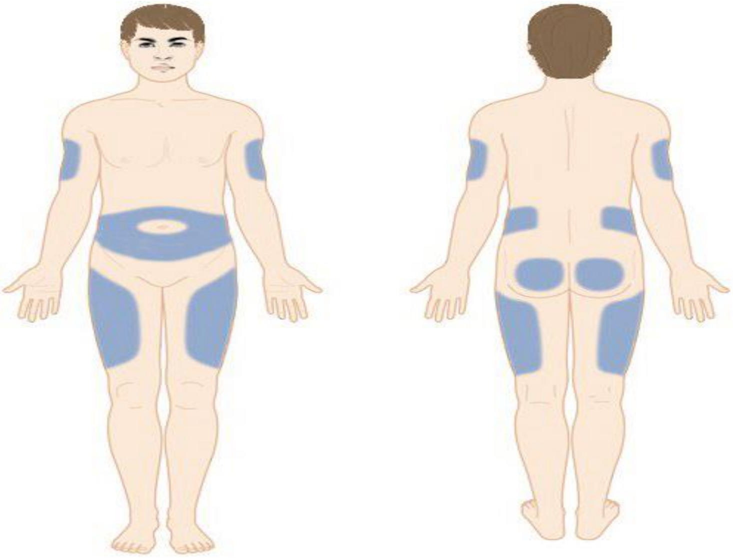

Kawasan yang boleh disuntik insulin

Suntik insulin pada bahu, paha, perut dan punggung di keseluruhan kawasan secara bergilir-gilir untuk mengelakkan terjadinya parut. Sebagai contoh, di kawasan perut adalah seperti dalam gambar:

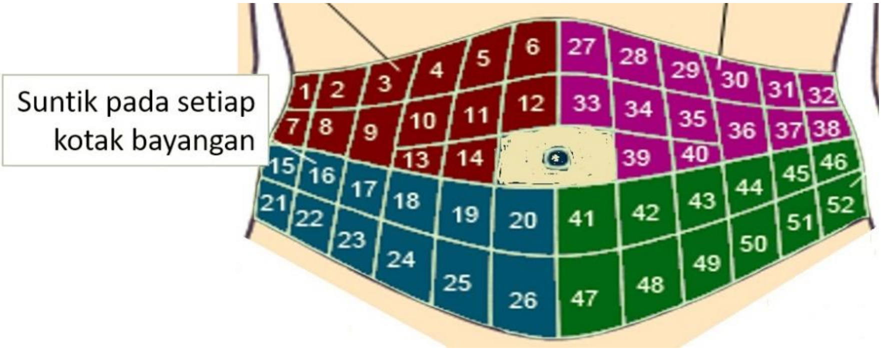

LAMPIRAN E: Teknik penggunaan insulin

A: Persediaan sebelum menyuntik insulin

1. Memastikan insulin dalam keadaan baik.

Periksa tarikh luput insulin. Pastikan insulin belum luput tarikh.

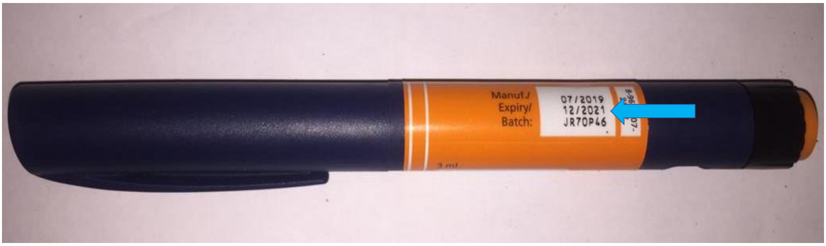

Periksa warna insulin- jernih (prandial) atau keruh (basal/pra-campuran). Jika bertukar warna, itu menandakan insulin telah rosak.

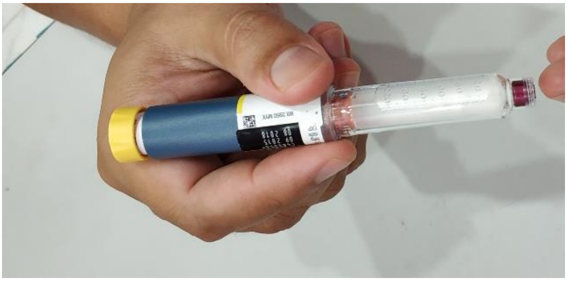

Jika terdapat mendakan, gerakkan pen insulin ke atas dan ke bawah seperti dalam gambar sebanyak 10 kali atau golekkan pen insulin di antara dua tangan selama 10 saat (untuk pen yang baru dibuka).

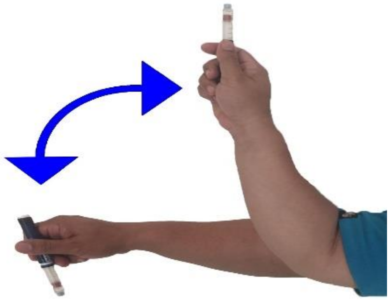

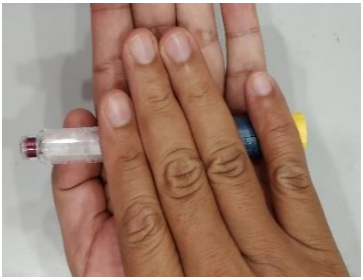

2. Memilih jarum yang berseuaian
    - Jarum 4/5 mm sesuai untuk semua.

3. Memasang jarum pada pen insulin
    a. Cabut kertas pembungkus jarum.
    b. Pasangkan jarum ke pen dengan memutar jarum ikut arah jam.
    c. Buka penutup luar jarum (lutsinar).
    d. Buka penutup dalam jarum.

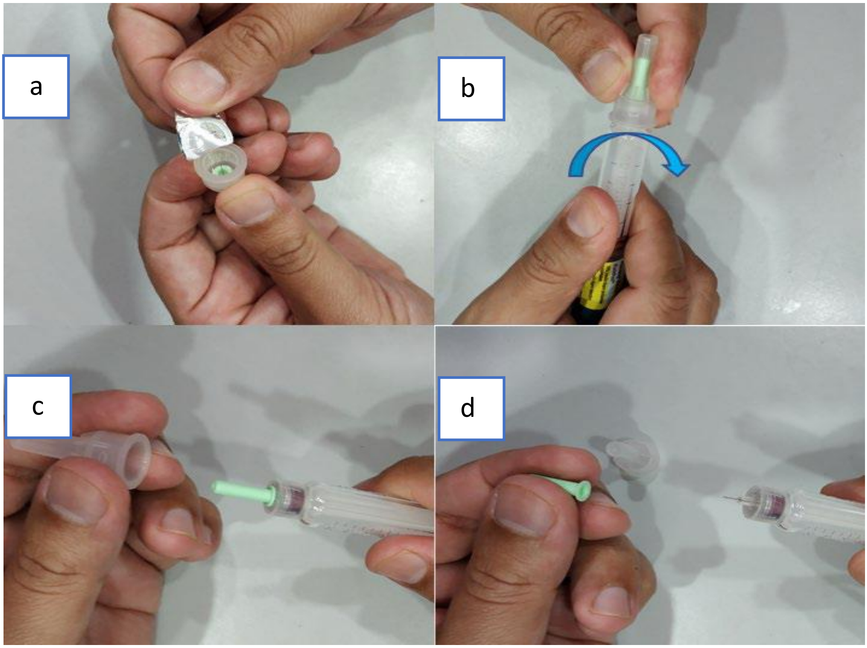

4. Menyediakan pen (*priming the pen*).
Pusingkan tombol dos ke 1 atau 2 unit

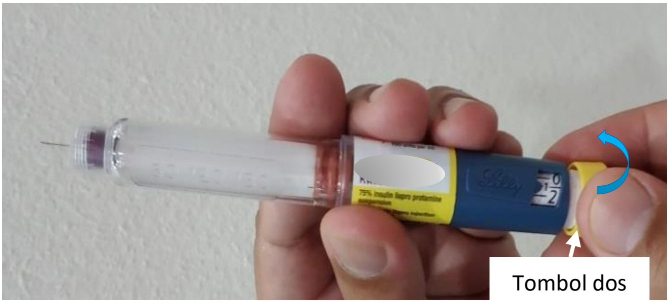

Pegang pen dalam keadaan menegak. Tekan tombol dos ke atas menggunakan ibu jari sehingga tombol dos menunjukkan angka 0.

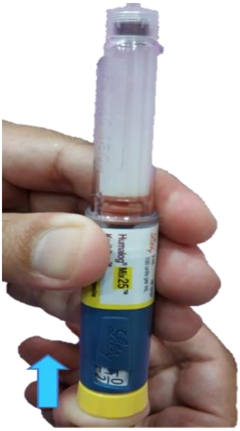

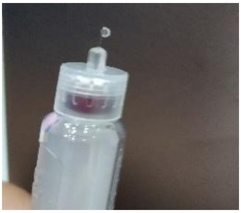

Perhatikan titisan insulin keluar. (Ulangi langkah a-c hingga anda dapat lihat titisan insulin keluar)

5. Menentukan dos insulin yang betul
Pusingkan tombol dos ke unit suntikan yang dikehendaki.
Contoh dalam gambar: 12 unit

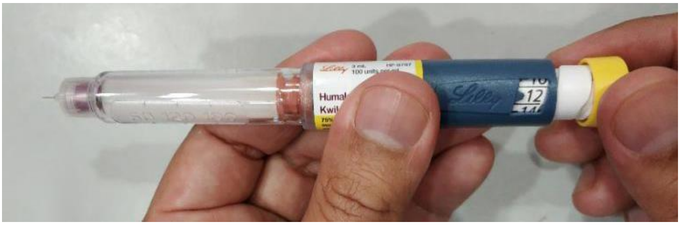

6. Pemilihan dan penyediaan tempat suntikan

a) Pilih lokasi tubuh yang akan disuntik seperti di lampiran C. Elakkan menyuntik di tempat yang sama atau berparut.

b) Dalam keadaan duduk, bersihkan tempat yang akan disuntik beberapa kali dengan kapas beralkohol.

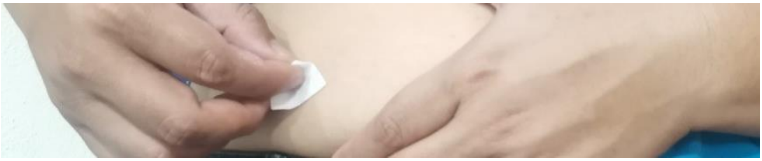

B: Kaedah menyuntik insulin

a. Genggam pen dengan empat jari. Letakkan ibu jari pada tombol dos.
b. Cubit bahagian kulit yang akan disuntik.
c. Suntikkan jarum pada sudut 90° dan lepaskan cubitan.
d. Menggunakan ibu jari, tekan tombol dos sehingga semua insulin masuk ke bawah kulit (tombol dos kembali ke 0). Tunggu 10 saat untuk mengelakkan insulin keluar dari tempat suntikan. Kemudian, keluarkan jarum.

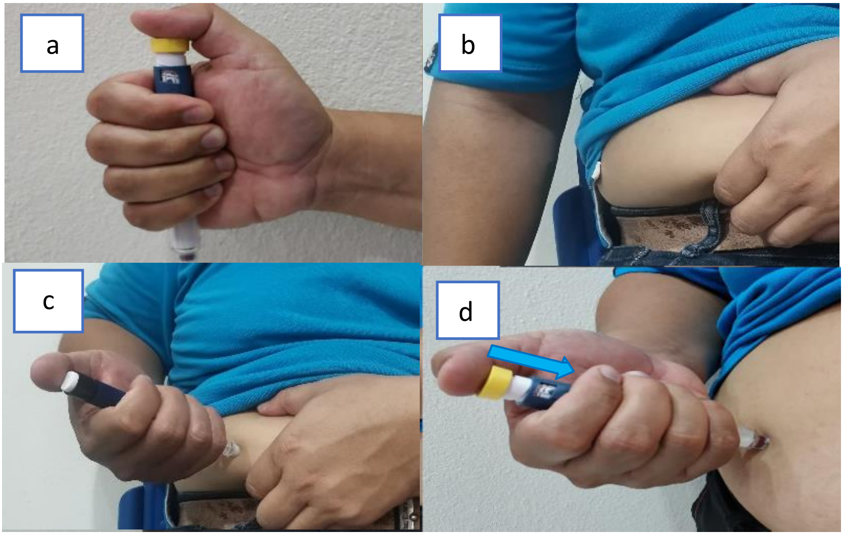

C: Menyimpan jarum dan pen insulin selepas suntikan.

a. Tutup jarum dengan penutup luar jarum (lutsinar).
b. Pusing jarum arah lawan jam hingga jarum terbuka dari pen.
c. Tutup pen dengan penutup pen.
d. Simpan kedua-duanya di dalam bekas yang sesuai.

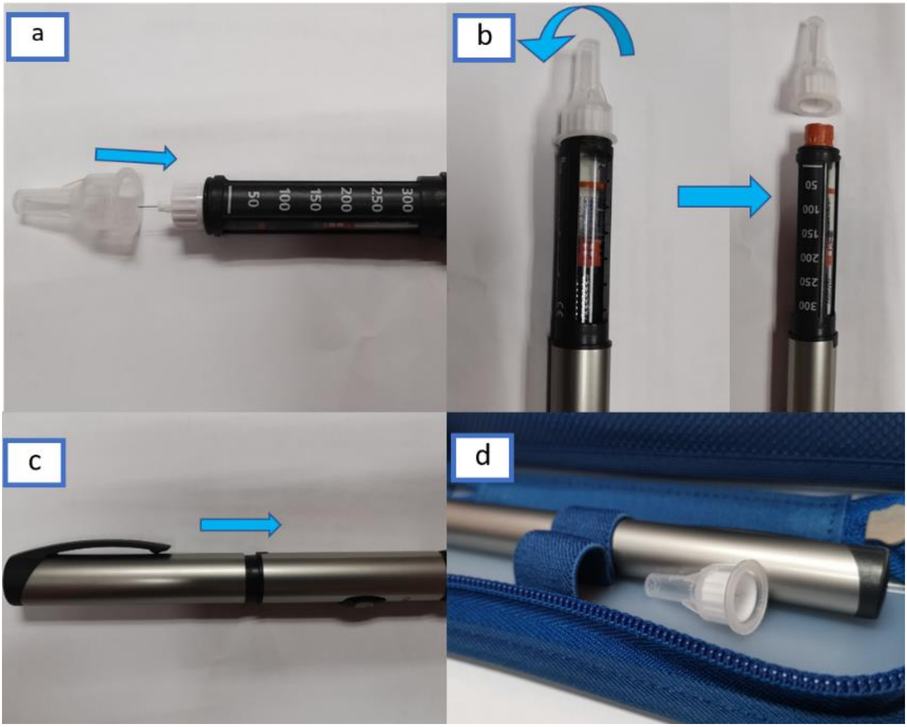

D: Kaedah melupuskan jarum suntikan yang telah digunakan tiga kali

Jangan tutup semula jarum dengan penutup jarum. Buang jarum ke dalam bekas khas yang bertutup dan selamat.

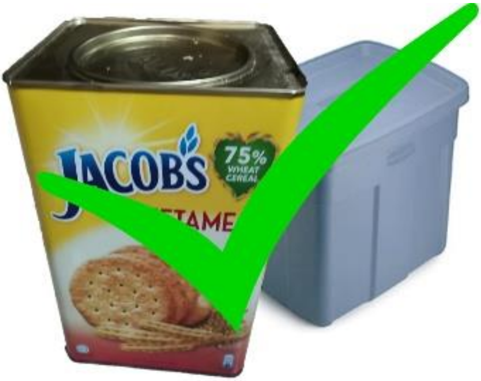

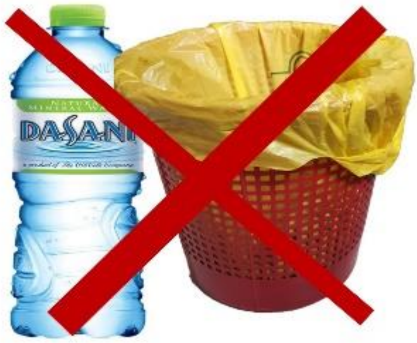

Apabila bekas telah penuh, tanam bekas tersebut.

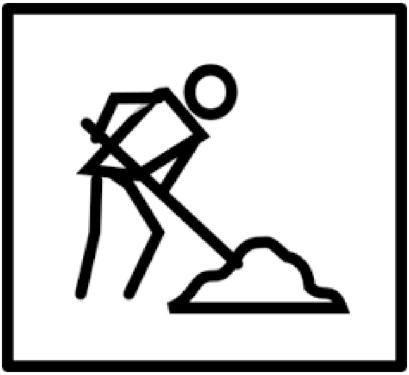

Jika anda tidak mempunyai kawasan yang sesuai untuk menanam bekas tersebut, berbincanglah dengan jururawat/ doktor anda.

LAMPIRAN F: Pengubahsuaian insulin ketika sakit

PENGUBAHSUAIAN INSULIN KETIKA HARI SAKIT

Semasa demam dan sakit, tubuh anda mengeluarkan hormon stres; adrenalin dan kortisol. Walaupun anda tidak makan, hormon ini menyebabkan **paras gula dalam darah meningkat.**

Anda digalakkan memeriksa paras gula darah empat kali sehari dan mengubah dos insulin berdasarkan paras gula yang diperiksa.

Paras gula darah	Pengubahsuaian dos insulin
< 4 mmol/L	Kurangkan 4 unit dari dos asal
11.1 - 17 mmol/L	Tambahkan 2 unit ke atas dos asal
17 - 22 mmol/L	Tambahkan 4 unit ke atas dos asal
> 22 mmol/L	Tambahkan 6 unit ke atas dos asal

Dapatkan rawatan **SEGERA** di hospital jika anda:

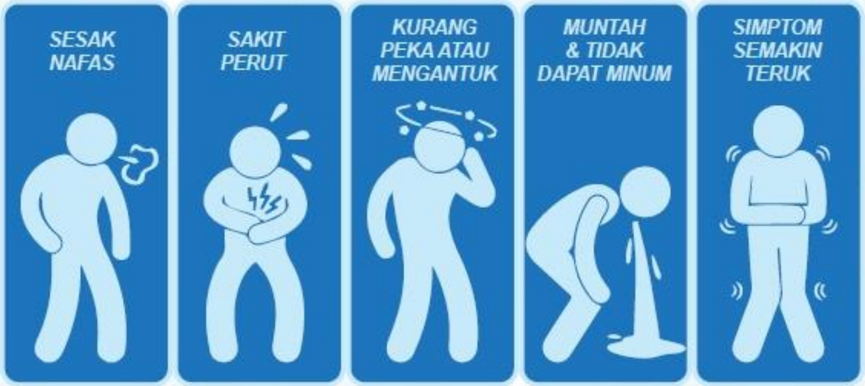

Sumber: Type 2 diabetes: What to Do When You Are Ill (Diabetes UK,2014)

LAMPIRAN G: Kaedah memeriksa aras gula

1. Basuh tangan dengan sabun dan keringkan.
2. Menyediakan alat pencucuk:
   a. Sediakan alat pencucuk dan jarum yang belum digunakan
   b. Buka penutup alat tersebut. Masukkan jarum yang baru ke tempatnya. Buka penutup jarum.
   c. Tutup alat penyucuk dan tarik ke belakang.

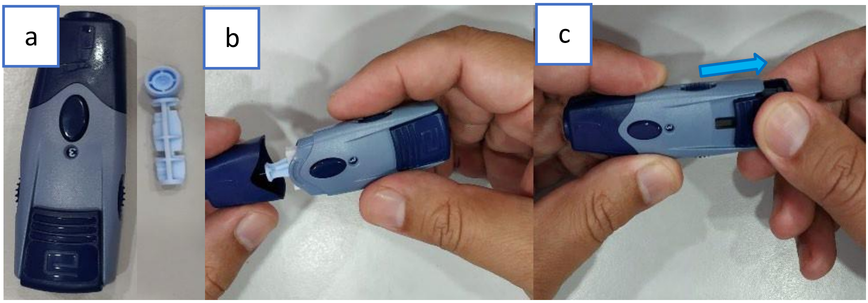

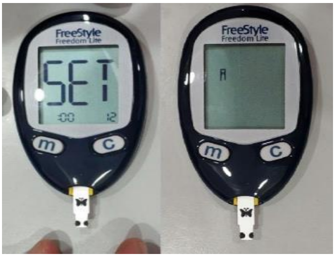

3. Ambil satu strip dari botol, dan masukkan strip ke dalam "glucometer". "Glucometer" akan menyala. Tunggu sehingga Label titisan darah timbul. Ini menandakan alat pengukur telah bersedia*.

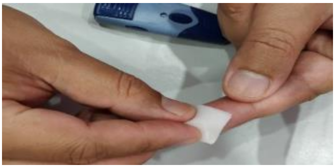

4. Gunakan kapas beralkohol untuk membersihkan jari yang akan disuntik.

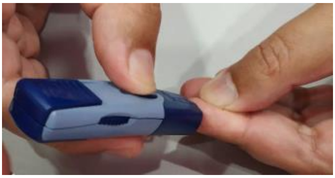

5. Suntik bahagian tepi hujung jari.

** Ikut arahan seperti dalam manual "glucometer" masing-masing. Ia mungkin berbeza antara jenama yang berbeza.*

6. Picit untuk mengeluarkan sedikit darah.

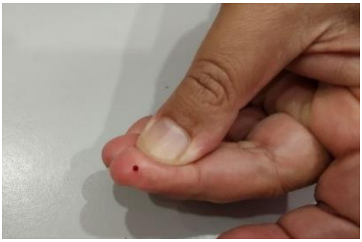

7. Letakkan titisan darah pada hujung strip sehingga terdengar bunyi “bip” atau skrin memaparkan isyarat tunggu. Tekan hujung jari dengan kapas untuk meghentikan pendarahan.

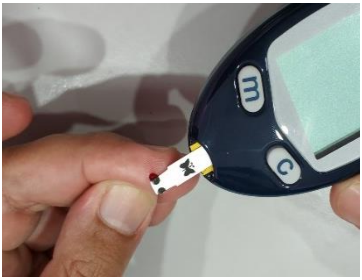

8. Baca aras gula pada “glucometer” dan rekodkan di dalam diari.

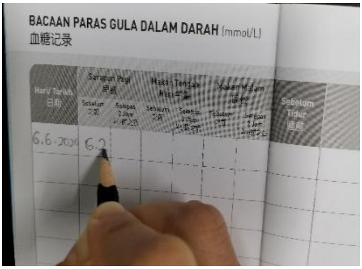

9. Dengan berhati-hati, pegang jarum di sisi dan tarik keluar dari alat pencucuk. Buang ke dalam tong alatan tajam.

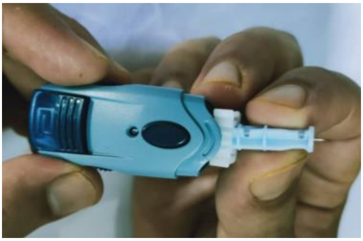

2
